# Supplementary material for: PopART-IBM, a highly efficient stochastic individual-based simulation model of generalised HIV epidemics developed in the context of the HPTN 071 (PopART) trial
Source: PLoS Comput Biol. 2021 Sep 2;17(9):e1009301. doi: 10.1371/journal.pcbi.1009301 (PMC8478209; doi:10.1371/journal.pcbi.1009301)
Supplement: S1 Appendix — Fig A. Annual mortality for men in Zambia by age group. Fig B. Annual mortality for women in Zambia by age group. Fig C. Annual mortality for men in South Africa by age group. Fig D. Annual mortality for women in South Africa by age group. Fig E. Definition of sexual activity levels in PopART-IBM. Fig F. HIV prevalence by sex, sexual activity level and age group for Zambia and South Africa in PC0 data. Fig G. Rate of partnership formation inside and outside the community, by age, activity class and sex for Zambia. Fig H. Rate of partnership formation inside and outside the community, by age, activity class and sex for South Africa. Fig I. Observed and fitted duration of partnerships so far for Zambia. Fig J. Observed and fitted duration of partnerships so far for South Africa. Fig K. Schematic of the HIV care cascade in the model. Fig L. Age distribution by sex for 4 randomly chosen calibrated IBM runs in Zambia. Fig M. Population size over time for 4 randomly chosen calibrated IBM runs in Zambia. Fig N. Percentage of the population who are in the low, medium and high sexual activity level groups over time, for the 4 randomly chosen calibrated runs. Fig O. Histogram showing the distribution of time from infection to AIDS death for individuals dying by 2004. Fig P. Histogram showing the distribution of time from infection to AIDS death for individuals dying in one calibrated run, by set-point viral load category. Fig Q. Distribution of PLHIV in the ART cascade over time for the four randomly chosen calibrated runs. Fig R. Changes in transitions between ART cascade states of PLHIV over time for one randomly chosen calibrated run. Fig S. Mean number of lifetime, current, and new partners of individuals currently alive in the simulation, plotted over time by sexual activity level for the 4 randomly chosen calibrated runs. Fig T. HIV prevalence for 200 uncalibrated runs. Fig U. Marginal posterior distributions for the 17 calibrated parameters. Table A. List of epidemiolo [file pcbi.1009301.s001.pdf]

Supplementary material for: PopART-IBM, a highly efficient stochastic individual-based simulation model of generalised HIV epidemics developed in the context of the HPTN 071 (PopART) trial

Michael Pickles, Anne Cori*, William JM Probert*, Rafael Sauter*,
Robert Hinch, Sarah Fidler, Helen Ayles, Peter Bock,
Deborah Donnell, Ethan Wilson, Estelle Piwowar-Manning,
Sian Floyd, Richard J Hayes, and Christophe Fraser

on behalf of the HPTN 071 (PopART) Study Team

*denotes equal contribution

Contents

1 Model structure overview **4**
1.1 Time evolution of the simulation 4
1.2 Stochasticity 5
1.3 Overview of the modelled population 5
1.4 Patches, mixing and migration 6
1.5 Terminology: characteristics, events and processes 6
1.5.1 Events and processes 6
1.6 Individuals and characteristics 7
1.6.1 Characteristics of individuals 7
1.7 Calibration 8
1.8 Model representation of the HPTN 071 (PopART) trial 9
1.8.1 Simulated counterfactual and predicted trial impact 10

2 Demographics **11**
2.1 Demographics at initialization of the model 11
2.2 New births and children 14
2.3 Entry into the adult population 17
2.4 Death due to natural causes (not AIDS-related) 17
2.4.1 Deriving mortality rates 18
2.5 Ageing 19

2.6 Sex ratio 24

3 Partnerships 25
3.1 Overview 25
3.1.1 Parameter estimation using the PC0 data 25
3.2 Sexual activity level 27
3.2.1 Defining activity levels using PC0 data 27
3.2.2 Maximum number of simultaneous partners 28
3.3 New partnership formation in PopART-IBM 29
3.3.1 Age Assortativity 29
3.3.2 Mathematical approach to balancing the number of partnerships formed between age groups 30
3.3.3 Mathematical approach to balancing the number of partnerships formed between sexual activity levels 32
3.3.4 Partners inside and outside the community 33
3.3.5 Estimating the rate of partnership formation from PC0 by sex, age and sexual activity level 34
3.4 Allowing flexibility around reported behaviours 34
3.5 The mechanics of partnership formation in PopART-IBM 36
3.6 Modelling partnership duration 38
3.6.1 Estimating partnership duration from PC0 38

4 HIV transmission, HIV testing and the ART cascade 42
4.1 Initializing the HIV epidemic 42
4.2 HIV transmission 43
4.2.1 HIV transmission, condom use and frequency of sex acts 44
4.3 Immediately post-infection 46
4.4 HIV progression when not on ART 46
4.5 Set-point viral load 47
4.5.1 Set-point viral load and HIV transmission 47
4.5.2 Set-point viral load and HIV progression in the absence of ART . . 48
4.6 HIV testing 48
4.6.1 Background HIV testing scheduling 48
4.6.2 CHiPs HIV testing scheduling 50
4.6.3 HIV testing from the individual's point of view 51
4.7 The care cascade 51
4.7.1 Time between an HIV-positive test result by CHiPs and initiation of ART 56
4.8 Emergency ART 57

5 Male circumcision 60
5.1 Types of circumcision 60
5.2 Traditional circumcision 60
5.3 VMMC 60

5.4 Circumcision coverage over time 61

6 Model validation **62**
6.1 Checking demographic processes 63
6.1.1 Cross-validation of population size counts and age list 63
6.2 Age distribution 64
6.3 Population growth 64
6.4 Sexual activity level 67
6.5 Checking HIV transmission and progression 67
6.6 Checking the ART cascade 71
6.7 Checking partnership formation and dissolution 71
6.7.1 Lists of individuals available for partnership formation 71
6.7.2 Number of partners outside the community 71
6.7.3 Rates of partnership formation 74
6.7.4 Assortativity of partnerships 74
6.7.5 Partnership duration by activity class and within vs between communities, as drawn at partnership formation. 75
6.8 Validating partnership outputs 75

7 Supplementary Results **77**
7.1 Uncalibrated runs and epidemic diversity 77
7.2 Posterior parameter plots 78

1 Model structure overview

In this document we describe in detail the different components of the PopART individual-based model (*PopART-IBM*). PopART-IBM is an individual-based mathematical model of HIV transmission in a heterosexual population. The model is designed to be granular enough to capture details that are reasonably thought to affect HIV epidemic dynamics, such as patterns of mixing and HIV testing by age and sex, and which can be supported by data. The model is coded in a modular structure, with components that each affect the predicted epidemic dynamics. Fig 1 in the main text shows the key components.

The model is primarily built in the C programming language, with data analysis carried out in R version 3.2.2 and Python 2.7.10. The full model code is available under the GNU General Public License version 3 at *https://github.com/BDI-pathogens/POPART-IBM.*

While PopART-IBM has been specifically built for the HPTN 071 (PopART) trial, it is envisaged to have many potential applications. Thus patches simply represent geographically separate regions in the model (and the number of patches can be one or more than one), in this document we use an example with n=2 patches, and 'patch 0' and 'patch 1' can be taken to be equivalent to 'trial community' and 'outside the trial community' in the context of the trial. It should be noted that the description of the 'intervention' provided here is specific to HPTN 071 (PopART), but that the coding reflects a multi-channel intervention improving HIV testing uptake, linkage to care, and voluntary male medical circumcision. Other interventions such as pre-exposure prophylaxis or condom use can be added with small modifications to the code.

1.1 Time evolution of the simulation

PopART-IBM is a discrete-time simulation, with one timestep is equal to 1/48 of a year (so that 1 month is equal to 4 timesteps). Throughout this document, time (and the variable t) refer to calendar time unless explicitly stated otherwise. The model is initially run for an extended period without HIV, to allow the demographics (in particular the age distribution) and sexual partner network to stabilize. We assess whether the sexual partner network has stabilized by examining whether diagnostic measures (the mean number of sexual partners over different periods) have reached equilibrium, while we assess convergence of the demographics by comparing the age distribution for each sex with that from United Nations Population Division (*UNPD*) estimates. Fig S in Section 6.8 shows that the mean number of sexual partners reaches after the model has run for approximately 30 years, suggesting that the sexual network has stabilized by this time. Fig L in Section 6.2 shows that a long period is indeed necessary prior to introducing HIV, approximately enough time so that no individuals remain who were alive at the start of the simulation. However, the age distribution in the simulation is indeed comparable to national estimates by the start of the HIV epidemic.

The time at which HIV is introduced is an input parameter that is calibrated, and once HIV is introduced the model can be run for extended periods, for example we have run the model with 2 patches and a population of 50,000 in each patch the model until

2100.

At each timestep each process is carried out sequentially for the adult population, before the next process (see section 1.5 for a description of what a process is, but broadly speaking it is something that can change one or more state variables for an individual). For instance the first process is deaths from natural causes, followed by entry of new individuals ageing into the adult population just before they turn 14 years old. Certain processes - those related to HIV testing and ART initiation, voluntary male medical circumcision, and other intervention activities - only occur after a certain time t in the simulation corresponding to when they began in real life in the given community.

1.2 Stochasticity

In PopART-IBM, events are drawn randomly, using the GNU Scientific Library (GSL) random number functions, as described below. At the beginning of each run, the GSL random number generator is seeded with a known integer that is recorded (this seed is treated as an input parameter for the model, so that it varies between runs, but the seed used for each run is kept along with the other model parameters for that run). In addition, at the time in the simulation when the PopART trial begins, the model can re-seed the random number generator; runs with the same initial seed but a different seed at the beginning of the trial are identical until the beginning of the trial but differ thereafter due to stochasticity. This allows us to assess how stochastic variation during the trial influences results.

Within the simulation, random numbers are drawn using the GSL random number functions: gsl_rng_uniform, for sampling uniformly from $[0, 1)$; gsl_rng_uniform_pos, for sampling uniformly from $(0, 1)$; gsl_rng_uniform_int(n), for sampling a random integer from 0 to n-1 inclusive; gsl_ran_bernoulli; gsl_ran_binomial; gsl_ran_exponential; gsl_ran_gamma; as well as gsl_ran_shuffle and gsl_ran_choose.

1.3 Overview of the modelled population

The modelled population is divided into children and adults. The adult population is the primary population in the model: this is the population which has characteristics and to which processes occur. In other words, detailed individual information is kept for each adult in the simulation, and this is updated over time as processes change individual characteristics. This assumes that children as defined in the model are not sexually active, or at risk of HIV infection (perinatal transmission is not implemented in this version of the model). For children the model therefore just keeps track of the number born at each timestep who will survive to adulthood. Note that for simplicity, in the main text we state that only adults aged 14+ are modelled, since we do not actually model anything happening to children until they reach 14. A full description of births and the transition to adulthood in the model is given in Section 2.

1.4 Patches, mixing and migration

PopART-IBM allows for one or more of *patches* (geographically separate regions). In this paper we consider two patches: patch 0 consists of a trial community ('inside patch'), and patch 1 consists of the neighbouring area, where individuals will have similar behaviour and may form sexual partnerships with individuals in patch 0, but where the trial is not carried out ('outside patch'). Thus both patches are assumed identical in demographics and HIV prevalence until the start of the trial. Note that the patch structure has been implemented so that each patch has its own parameters, so that the assumption of identical parameters is not built into the model itself.

By default, in patch 1, unlike patch 0, there is no PopART intervention increasing the rate of HIV testing, linkage to care and voluntary male medical circumcision (VMMC) once the trial starts, while the guidelines for initiating antiretroviral therapy (ART) in patch 1 change as local implementation occurred in response to changes in national guidelines. Using different settings in the model, the PopART intervention can be included in both patches, or in neither.

Individuals may have sexual partners either in their own patch (community) or outside (i.e. with individuals in the other patch). The interaction between patches is through sexual partnerships: individuals in two different patches may form sexual partnerships together, reflecting the reality that people often have sexual partners outside a given community. Migration, either between patches or into/away from a patch, is not currently modelled.

1.5 Terminology: characteristics, events and processes

We now establish the terminology which will be used to describe PopART-IBM. In each patch the model has *individuals* with *characteristics*, and *events* occur to some of these individuals at each timestep. Characteristics and events are intertwined. The events that happen to individuals depend on their own characteristics and those of other individuals (such as sexual partners), as well as other factors such as time t (note that throughout t refers to discrete time, with timesteps of 1/48 year). Characteristics are changed by events, for example the event of HIV infection changes an individual's HIV status.

1.5.1 Events and processes

Each individual experiences different events over the course of their life, such as forming new sexual partnerships, getting older, testing for HIV, and dying. The events that occur to someone may depend on their characteristics, the characteristics of the population as a whole, and time t. For example HIV infection can occur to an individual if they are HIV-negative and they have a partner who is HIV-positive. A person may initiate ART if they are HIV-positive and aware of their serostatus, and meet the eligibility criteria for ART at the given time t. We group these events into different *processes*. A process groups together related events such that at most one event happens to any given person at a single timestep. However, two events can happen to the same person in the same

timestep provided that the events belong to different processes (for example someone can acquire an extra sexual partner and become HIV infected at the same timestep, either by the new partner or an existing one). For each timestep, processes always occur in the same fixed order. The list of processes are:

- Demographic processes (births, ageing, death apart from AIDS-related death);
- Sexual partnership breakup;
- Sexual partnership formation;
- HIV acquisition;
- HIV-related events when not on ART (disease progression, initiation of ART once CD4 count drops below 200 cells/mm^3, AIDS-related death);
- Background (i.e. non-PopART) HIV testing and the ART cascade: includes the processes of HIV and CD4 testing, entering care or initiating ART, dropping out of care and re-entering the cascade;
- HPTN 071 annual CHiPs visits (HIV testing);
- Voluntary male medical circumcision (VMMC).

For computational efficiency most processes are scheduled, so that the next event of a given process for each individual is determined in advance. For each scheduled process we sweep through the list of individuals who have an event from that process scheduled to occur at that timestep before moving on to the next process. For a non-scheduled process, we sweep through the population of people who are affected by that process (for example HIV infection occurs only to HIV-negative individuals with at least one serodiscordant partner) before moving on to the next one.

1.6 Individuals and characteristics

1.6.1 Characteristics of individuals

Each individual has a large number of characteristics stored at any given time, including both validation characteristics and epidemiological characteristics, as well as other characteristics required by the computational algorithms used. Validation characteristics are used to validate the model by checking for self-consistency within the model and for comparison with data such as national-level DHS surveys in Zambia or HSRC surveys in South Africa. Epidemiological characteristics are those relevant to HIV transmission in some way. The epidemiological characteristics of the adult population are listed in Table A. Some epidemiological characteristics of an individual are immutable once the individual enters the adult population (ID number, sex, date of birth, sexual activity level, maximum number of simultaneous sexual partners), some are set by a process during the simulation (having previously been assigned a placeholder value of -1) but then

Characteristic	Description
ID number	Unique identifier for each person.
Sex	Whether the person is male or female.
Date of birth	Used to derive age and age group.
Sexual activity level	Low, medium or high (determines preferred number of partners).
HIV status	Divided into uninfected, acute/early infection and chronic infection.
Time of HIV seroconversion	
Current CD4 category	Categories are: CD4>500, 350-500, 200-350, and CD4≤200. Determines eligibility for ART, infectivity and AIDS-related death rate.
Set-point viral load	Measured in $\log_{10}$ copies/ml. Determines HIV progression rate and infectivity.
ART status	Divided into never tested positive, positive but not yet on ART (or dropped out), early ART (on ART for <2 months), on ART for ≥2 months and virally suppressed, on ART for ≥2 months and not virally suppressed.
Circumcision status	For men stores current circumcision status, including whether the circumcision was voluntary male medical circumcision (VMMC) or traditional circumcision.
Number and IDs of current partners who live in the community	This is a list of the current sexual partners of the individual who are in the same patch.
Current partners outside the community	A list of the current sexual partners of the individual who are in a different patch.
Number and IDs of HIV+ partners	List of those sexual partners (regardless of patch) who are HIV positive.
Maximum number of partners	Maximum number of concurrent partnerships the individual can have at any time.

Table A: List of epidemiological characteristics of each adult individual stored by PopART-IBM.

remain fixed thereafter (time of HIV seroconversion, set-point viral load), and the remaining characteristics (such as HIV status) can change over time in response to specific processes.

Other quantities in the model are derived using the stored characteristics, for example the age of the individual is calculated as necessary based on the date of birth and current time, with the choices driven by memory and computational efficiency considerations.

1.7 Calibration

The PopART-IBM has over 300 parameters spanning the natural history of HIV, sexual partnerships, demographics, HIV-related prevention and treatment.

For the HPTN 071 (PopART) trial a number of parameters are estimated directly

from PopART data, and the remainder are drawn from the literature, including country-specific statistics collected from UNPD, and Demographic and Health Surveys (*DHS*) and the South African National HIV Prevalence, Incidence and Behaviour Surveys run by the Human Sciences Research Council (referred to here as *HSRC surveys*). We specify the parameter value/range used, and describe the data sources as well as any additional analyses used, in the relevant section for each parameter.

Most parameters are fixed, but a subset of parameters is allowed to vary, and these parameters are fitted during the calibration process (parameters are listed in later sections). While any subset of parameters can be chosen to vary, we chose the varied parameters because they were either unknown but thought to be potentially influential (e.g. sexual mixing assortativity by sexual activity level), or could be directly informed by calibration (for example time to initiate ART is a parameter which can be informed during calibration by data on ART uptake).

PopART-IBM is parameterised using a Bayesian framework called Approximate Bayesian Computation (ABC). ABC is a suite of algorithms that approximate a conditional probability density function as described in Beaumont et al. [1]. In some situations a likelihood function is not available or is intractable, but a simulation from a corresponding distribution, here by using the PopART-IBM, is possible. The simulated projections therefore replace the intractable likelihood. A distance measure compares simulated and observed summary statistics. A simulated projection, for a given IBM input parameter combination, is accepted if the distance measure is below a predefined threshold. The resulting distribution of the model projections is proportional to the posterior distribution given the data and the IBM. As summary statistics, the sex and age-group stratified HIV prevalence from historical data as well as from the CHiPs data are used[1]. These are complemented with the proportion of people living with HIV (PLHIV), by age group and sex, who are aware of their status and on ART, and the proportion of all PLHIV who are virally suppressed (again stratified by sex and age group). We use the algorithm proposed by Lenormand et al. [2], the adaptive population Monte Carlo-ABC (APMC-ABC) algorithm (see appendix in [2] for full details of the algorithm), implemented using the R package EasyABC version 1.5. ABC inference for the PopART simulations is based on 2,000 simulation in each step, with an acceptance rate of 50%. The APMC-ABC algorithm stop if the proportion of simulations with decreased distance measure is below a threshold, here chosen as 9%. There are 1,000 accepted simulations produced in the final APMC-ABC step. Unless otherwise specified, all model outputs are based on quantiles of this posterior distribution.

1.8 Model representation of the HPTN 071 (PopART) trial

In the HPTN 071 (PopART) trial, household-based testing is carried out by by community health workers, known as *CHiPs* (community HIV care providers) in trial Arms A

[1]'CHiPs data' refers to data collected by Community HIV-care Providers, CHiPs, who go from household to household offering HIV testing and prevention services; the age and sex of individuals who consent to receive services from CHiPs is recorded, along with HIV status - either self-reported positive, or rapid HIV test result, and whether they are on ART if they self-report as HIV positive.

and B (Arm C is the control arm of the trial). In all arms (A,B,C) individuals may test through other routes outside the trial, such as in clinics or as part of antenatal care, which we collectively term *'background testing'*. Similarly men may take up VMMC through background channels, as well as (in Arms A and B) through referral by CHiPs. Thus HIV testing, linkage to care and VMMC all occur in Arm C communities, but at lower rates of uptake and linkage (than Arms A and B) and with different eligibility criteria for initiating ART (than Arm A).

The trial intervention package consists of the following:

- Additional HIV testing through CHiPs teams;
- Enhanced linkage to care;
- Immediate ART regardless of CD4 count in Arm A communities (Arm B communities follow national guidelines, including any changes to guidelines over time);
- Increased uptake of voluntary male medical circumcision (VMMC).

For changes that occurred during the trial the date when the change happened locally in the community was documented, and so is used in the model: for example for changes in ART guidelines, the date used reflects when the clinics in the community began using the new guidelines rather than when the official change in national policy was made, reflecting the fact that implementation does not coincide exactly with policy. For earlier changes, the date of the change in national policy is used.

1.8.1 Simulated counterfactual and predicted trial impact

The ideal estimate of impact would be a comparison of a population to itself with the exposure removed[3]. Mathematical modelling allows us to approach this by generating a *simulated counterfactual*, namely a simulation using exactly the same parameter values except for those relating to the intervention. For PopART-IBM in the simulated counterfactual there are no CHiPs visits (and consequent improved linkage to care), and guidelines for initiating ART follow national guidelines as for an Arm C (control) community. The predict impact is computed for each of the 1,000 parameter sets in the posterior.

2 Demographics

Within PopART-IBM the demographic component consists of the following processes:

- New births
- Entry into the adult population
- Deaths due to natural causes (not HIV-related)
- Ageing

Each patch contains an open population where individuals are born, age, and die. The size of the population in each patch changes over time, driven by differences in fertility and mortality rates. These rates are based on the country-specific United Nations Population Division (*UNPD*) World Population Prospects (*WPP*) estimates [4]. The initial adult population size in the model is adjusted to produce a population size of around 50,000 individuals aged 14 and above by 2015, similar to that of a HPTN 071 (PopART) community. The model is initialised at time t_0, chosen here to be 1900, well before the introduction of HIV, to allow the model time for the diagnostic measures for demographics and sexual partnerships to stabilise (see Figs S and L).

The model treats adults and children separately. Within the model **adult** means anyone aged 14 years old or above. New adults can, but do not need to, acquire sexual partners (i.e. become sexually active). Adults form the primary population: they have full characteristics (HIV status, sexual activity level, etc), and processes occur to them, including forming sexual partnerships, getting infected with HIV, initiating treatment and death. The model only keeps track of the number of children in each patch born at each timestep, and nothing further happens to them until they reach adulthood. As described in section 2.2, rather than model explicitly death during childhood, perinatal and childhood mortality is taken into account by discounting the fertility rate so that only children who will survive to adulthood are included. The threshold of 14 years and above is intended to included to capture the majority of time of exposure to HIV risk through sexual activity.

2.1 Demographics at initialization of the model

At the start of each simulation ($t = t_0$) the adult population is set up. This is done by first choosing the number of individuals of a given sex g, sexual activity level r and age group a at that time $N^g_{a,r}(t_0)$, and then generating that many individuals with those characteristics (i.e. sex and sexual activity level, with date of birth chosen so that age is in the given age group) in the model. Here we use the age groups 13-17, 18-22, 23-29, 30-39, 40-49, 50-59, 60-79, 80+ (so that the number of age groups $N_{age} = 8$) and the sexual activity levels low, medium, high (so that $N_{risk} = 3$). Note that we use C-style indexing so that e.g. $a = 0$ is the first age group, and summation over age groups means summing from $a = 0$ to $a = N_{age} - 1$. Summation over g means summing over sex (male/female).

The simulation is started well prior to the introduction of HIV, so all the initialised individuals are HIV-negative. The total number of individuals in a patch at the start of the simulation is specified as an input parameter N_0. N_0 satisfies the constraint that it is equal to the sum of the number of men and women in every age and sexual activity level at t_0:

$$N_0 = \sum_{g} \sum_{a=0}^{N_{age}-1} \sum_{r=0}^{N_{risk}-1} N^g_{a,r}(t_0)$$

We calculate the $N^g_{a,r}$ at time t_0 from N_0 as follows: we assume that the initial proportion in each age group a is equal to $f_0^{by\ age}[a]$, using the UNPD estimates from 1950, the earliest time available (and assume that this is independent of sex). Note that we adjust the UNPD numbers since PopART-IBM uses different age groups to UNPD (UNPD age groups are all 5 year age groups 0-4, 5-9, 10-14 etc), using the crude assumption that within each UNPD age group the number of people in each single year age group is the same. Since demographic processes are given a long time for the age distribution to converge prior to the introduction of HIV, the exact initial conditions will not matter and hence the crudeness of this approximation is unimportant. Within each age group the fraction who are male is set by the parameter $f^{sex=M}$, assuming age and sex are independent, and the fraction of individuals of a given sex in each sexual activity level is $f_0^r[g]$.

Hence

$$N^g_{a,r}(t_0) = \begin{cases} f^{sex=M} f_0^{by\ age}[a] f_0^r[g] N_0, & (g = M, Male) \\ (1.0 - f^{sex=M}) f_0^{by\ age}[a] f_0^r[g] N_0, & (g = F, Female) \end{cases}$$

Once we know $N^g_{a,r}(t_0)$, we generate that corresponding number of individuals in the model of sex g and sexual activity group r, who are in age group a, rounding down to the nearest integer using the $floor()$ function. Their exact age is calculated afterwards, along with other characteristics, using the same process as that of individuals reaching adulthood later in the simulation (described in section 2.3), apart from age which is predefined for individuals reaching adulthood. Here, for initialising the population, individuals are assigned a date of birth based on their exact age and the current time as follows: their exact age is drawn uniformly between the minimum and maximum of that age group a excluding the the boundary values - e.g. if $a = 1$ their age is drawn from the open interval (18,23)[2] - to avoid potential issues with boundaries. In the youngest age group $a = 0$ the age is drawn from the open interval (14,18). For the oldest age group the age is drawn from (60,80) so that no individual is aged > 80 at the start of the simulation. Each individual is assigned a patch-specific ID, and is set to be uninfected with HIV, as the simulation starts before the introduction of HIV. At the start of the simulation individuals have no sexual partners.

At initialisation a proportion $p_{\text{child circ}}$ of men are taken to be circumcised traditionally. If the number of men in a given age group a and sexual activity level r is $N^m_{a,r}(t_0)$

[2]They can thus be aged 18.001 or 22.999 years but not 18.000 or 23.000 years.

then the first $floor(N^m_{a,r}(t_0) * p_{\text{child circ}})$ of the men in that age group are taken to be circumcised traditionally. As described later, traditional circumcision may have a different effectiveness to voluntary male medical circumcision for reducing HIV susceptibility within PopART-IBM [5]. Voluntary male medical circumcision is taken to not exist at that time.

Other characteristics related to the technical running of the model - scheduling and validation characteristics - are given appropriate default values, the same as is described in detail in section 2.3.

The list of children is populated at the start of the simulation by assuming that the fertility rate, population size, and age distribution were all constant prior to the start of the simulation. The number of children who had been born in each timestep prior to the start of the simulation is therefore drawn from a binomial distribution using the fertility rate and adult female population size at the start of the simulation (the exact details are described in more detail in 2.2). While the simulation has a long time prior to the introduction of HIV so that the exact choice of initial conditions should not affect the demographics by the time the HIV epidemic begins, the age distribution by sex is checked against UNPD estimates at several time points to ensure that there are no issues (see Fig L in Section 6.2).

**Parameter**	**Value**		**Notes**
	**Zambia**	**South Africa**	
t_0 , start of simulation	1900	1900	Assumption to give demographics and partnerships time to stabilize
N_0, initial population size aged 14+	1600	2800	Gives a population size of approximately 50,000 adults by start of trial.
Proportion in each age group at start of simulation ($f_0^{by\ age}[a]$)			
14-17	0.1583	0.1339	All based on UNPD WPP [4]
18-22	0.1613	0.1420	
23-29	0.1885	0.1724	
30-39	0.1983	0.2046	
40-49	0.1353	0.1498	
50-59	0.0828	0.1045	
60-79	0.0755	0.0928	
80+	0	0	
Proportion by activity group when entering population ($f_0^r[g]$)			
Low-activity men	30-60%		Based on variability between communities from PC0
Low-activity women	30-60%		
Medium-activity men (of those not in low activity group)	50-99%		
Medium-activity women (of those not in low activity group)	50-99%		
Proportion of new adults who are male $f^{sex=M}$	0.507	0.505	CIA world factbook[6]

Table B: Parameters related to initialization of the population in PopART-IBM. PC0 = HPTN 071 (PopART) baseline population cohort survey

2.2 New births and children

At each timestep the model calculates the number of new children born in that timestep who will survive to reach adulthood. This is drawn as the sum over a of binomial random variables $Binomial(N_f^a(t), p_{child}^a(t))$, where $N_f^a(t)$ is the female population size aged a years at time t and $p_{child}^a(t)$ is the estimated probability that a woman aged a years has a child in that timestep that will survive to adulthood.

The probability $p_{child}^a(t)$ is derived from UNPD WPP age-specific fertility rates for the given country, during the periods 1950-55, 1955-60,... 2095-2100, using the UNPD medium fertility variant for future projections as described in [7] (see Tables C and D), and discounting for child mortality, as described next. UNPD uses age groups 15-19, 20-24, ..., 45-49. In PopART-IBM the fertility rate is assumed for simplicity to be constant within each age group (for example a woman age 24 will have the fertility rate of 20-24 olds at that time; in the following year she will have the fertility rate of 25-29 year olds). Fertility rates are interpolated between different calendar time periods, taking the UNPD estimate for 1950-55 to correspond to 1950, etc. Women aged outside the ages 15-49 are assumed to be negligibly fertile. Prior to the first time point available from the UNPD data, we use the fertility rate of that time (so that prior to 1950 the 1950-55 fertility rates are used). Note that as we run the model for an extended period, starting well before 1950, prior to the introduction of HIV, matching the exact demographics prior to 1950 is unnecessary (and in Section 6.2 we show that demographics have reached convergence after the introduction of HIV). Similarly if the model is run after 2100, the 2095-2100 fertility rate is used.

To calculate $p_{child}^a(t)$ this fertility rate is then discounted by the probability $p_{childmortality}(t)$ of the child dying before it reaches adulthood, which is calculated as follows:

$$p_{childmortality}(t) = 1-(1-\text{mortality\_rate}_{under5}(t))^5*(1-\text{mortality\_rate}_{age5-10}(t+7.5))^5$$

where $\text{mortality\_rate}_{under5}(t)$ and $\text{mortality\_rate}_{age5-10}(t+7.5)$ are based on the UNPD WPP estimates of annual mortality rates aged 0-5 and 5-9 at time t and t+7.5 respectively (as described in Section 2.4.1 we use a regression model fitted to UNPD data, to enable us to remove the effects of HIV-related mortality, and then estimate the mortality rate at the given time in PopART-IBM using the regression model). The different times t and $t+7.5$ are to account for changes in mortality rates between the time of birth and when the child reaches the given age range: for the former it is assumed that the majority of mortality aged 0-4 occurs around the time of birth (i.e. at time t) while mortality in children 5-9 is assumed to occur uniformly during that age, so on average 7.5 years after the time of birth t. Mortality in children aged 11-13.99 is assumed negligible in comparison and is ignored. The regression coefficients used in estimating child mortality are given in Table F. Given this mortality rate $p_{childmortality}(t)$ and the time-interpolated UNPD fertility rate $\text{Age\_specific\_fertility}(a, t)$, the probability that a woman of age group a has a child at time t that survives to adulthood is then:

$$p_{child}^a(t) = \text{dt} * (1 - p_{childmortality}(t)) * \text{Age\_specific\_fertility}(a, t)$$

For technical reasons, because there is continuous introduction of new adults throughout the year, but yearly age cohorts are stored (of individuals who are the same age at the start of the year) for algorithmic efficiency, children become adults in the model one timestep before they turn 14. When a child reaches this age they are removed from the list of children, and a corresponding adult individual is created, who is assigned detailed individual information at that point. This is described in detail in section 2.3.

	**Fertility rate per 1000 women by age group Zambia**						
Period	15-19	20-24	25-29	30-34	35-39	40-44	45-49
1950-1955	171.9	282.0	261.3	240.7	211.5	140.4	42.1
1955-1960	175.8	288.3	267.2	246.1	216.2	143.5	43.0
1960-1965	182.1	298.7	276.8	255.0	224.0	148.7	44.6
1965-1970	188.5	309.1	286.5	263.9	231.9	153.9	46.2
1970-1975	189.1	310.2	287.5	264.8	232.6	154.5	46.3
1975-1980	187.9	308.1	285.5	263.0	231.1	153.4	46.0
1980-1985	178.3	292.4	271.0	249.6	219.3	145.6	43.7
1985-1990	171.0	312.4	295.9	253.2	190.7	83.3	28.5
1990-1995	162.7	297.2	281.5	240.9	181.4	79.2	27.1
1995-2000	143.7	282.3	274.1	236.4	178.3	84.6	30.6
2000-2005	143.3	268.9	258.1	235.5	187.4	88.3	28.5
2005-2010	122.0	273.3	267.3	229.0	176.9	86.8	24.6
2010-2015	103.3	267.3	265.0	210.3	152.4	71.8	19.9
2015-2020	77.5	272.4	278.7	195.2	129.2	60.7	14.6
2020-2025	65.5	264.8	275.4	182.9	115.9	54.3	12.1
2025-2030	55.5	256.4	271.0	171.8	104.4	48.6	10.1
2030-2035	47.3	247.2	265.7	161.7	94.5	43.6	8.5
2035-2040	40.5	237.9	260.0	152.8	86.0	39.3	7.1
2040-2045	35.0	228.5	254.1	145.0	78.7	35.5	6.0
2045-2050	30.4	219.3	248.1	138.4	72.6	32.3	5.1
2050-2055	26.6	210.0	242.0	132.6	67.4	29.5	4.4
2055-2060	23.5	201.1	236.1	127.9	63.0	27.1	3.8
2060-2065	20.8	192.1	230.0	123.8	59.3	25.0	3.3
2065-2070	18.7	183.7	224.3	120.6	56.3	23.2	2.9
2070-2075	16.8	175.1	218.4	117.8	53.7	21.5	2.5
2075-2080	15.3	167.1	212.9	115.9	51.7	20.2	2.2
2080-2085	14.0	159.0	207.2	114.4	50.0	19.0	2.0
2085-2090	12.9	151.2	201.9	113.4	48.7	17.9	1.7
2090-2095	12.0	143.5	196.5	112.9	47.8	17.0	1.6
2095-2100	11.3	135.9	191.1	112.8	47.1	16.1	1.4

Table C: *Fertility rate for Zambia over time (per 1000 women), using UN Population Division World Population Prospects estimates[4]*

	Fertility rate per 1000 women by age group South Africa						
Period	15-19	20-24	25-29	30-34	35-39	40-44	45-49
1950-1955	66.8	265.0	291.9	242.2	189.8	132.0	72.3
1955-1960	65.7	260.8	287.3	238.3	186.7	130.0	71.2
1960-1965	64.7	256.6	282.7	234.5	183.7	127.9	70.0
1965-1970	60.4	239.7	264.1	219.1	171.7	119.5	65.4
1970-1975	76.1	233.9	253.6	211.0	160.2	105.1	54.0
1975-1980	86.1	217.3	231.8	193.6	142.3	87.4	41.5
1980-1985	93.6	201.1	211.3	177.0	125.9	71.7	30.5
1985-1990	95.4	179.4	185.7	155.9	107.3	56.0	20.4
1990-1995	90.8	152.2	155.2	130.8	86.9	41.0	11.7
1995-2000	80.6	140.5	142.5	111.5	74.4	31.0	10.3
2000-2005	70.7	139.0	141.8	105.6	67.4	27.1	8.8
2005-2010	59.2	131.7	135.1	95.9	58.4	22.6	7.1
2010-2015	50.9	129.0	133.1	90.2	52.3	19.4	5.9
2015-2020	40.2	129.4	136.0	85.6	45.4	15.5	4.3
2020-2025	35.0	125.3	133.8	82.3	42.0	13.8	3.6
2025-2030	30.6	121.1	132.0	80.1	39.4	12.4	3.1
2030-2035	26.9	116.6	130.0	78.7	37.4	11.3	2.6
2035-2040	23.8	112.0	128.5	78.1	36.1	10.4	2.2
2040-2045	21.3	107.4	127.3	78.4	35.3	9.8	1.9
2045-2050	19.1	102.7	126.5	79.5	35.0	9.3	1.7
2050-2055	17.2	97.8	125.7	81.5	35.3	8.9	1.4
2055-2060	15.6	92.8	125.2	84.3	36.0	8.7	1.3
2060-2065	14.2	87.5	124.7	87.8	37.1	8.6	1.1
2065-2070	12.9	82.0	124.1	92.1	38.7	8.6	1.0
2070-2075	11.8	76.2	123.3	97.0	40.8	8.7	0.9
2075-2080	10.8	70.1	122.0	102.5	43.2	8.8	0.8
2080-2085	9.9	63.8	120.3	108.5	46.2	9.0	0.7
2085-2090	9.0	57.3	118.1	114.9	49.5	9.3	0.6
2090-2095	8.2	50.8	115.1	121.4	53.3	9.6	0.5
2095-2100	7.4	44.4	111.7	128.2	57.6	10.0	0.5

Table D: *Fertility rate for South Africa over time (per 1000 women), using UN Population Division World Population Prospects estimates[4]*

2.3 Entry into the adult population

For each individual reaching adulthood in a given patch, a number of characteristics are assigned, mirroring the assignment that occurs when the population is initialised at the start of the simulation. Firstly they are assigned an ID number and a date of birth, with the latter calculated as the time at which they would have been born given that they are now 13.99 years old. Their sex and sexual activity level are drawn randomly, with the probability that the individual is male given by the parameter $f^{sex=M}$, while the probability that an individual is in sexual activity level r is $f_0^r[g]$, and depends on the chosen sex g. Mother-to-child transmission is not modelled, and all individuals are assumed to be HIV-negative when entering the adult population.

On entry to the adult population individuals are able to form sexual partnerships; they are not assigned any sexual partners at entry, but they are added to the pool of potential sexual partners, so that they may acquire sexual partners thereafter (see Section 3).

Amongst male individuals reaching adulthood a proportion $p_{\text{child circ}}$ undergo traditional male circumcision. In the HPTN 071 (PopART) trial $p_{\text{child circ}}$ comes from data from the baseline Population Cohort survey (PC0) on the proportion of men in each community who self-report being circumcised by a traditional practitioner. PC0 data shows that traditional circumcision in Zambia generally occurs during childhood [5], before the onset of sexual activity, and thus individuals are not circumcised in PopART-IBM after reaching adulthood. Voluntary male medical circumcision is generally carried out among adults, so within the model it only occurs to individuals through the VMMC process (see Section 5.3).

Immediately after reaching adulthood, individuals are not scheduled to undergo any events - they have no partners (so cannot break up partnerships), and are HIV-negative (so will not undergo either CD4 progression or any steps in the ART cascade). They are also assumed to not have scheduled any HIV testing at that point, although they may be tested in future.

Similarly other characteristics relevant to the technical details of the model such as validation characteristics and cumulative counters are initialised to appropriate values (generally 0 for cumulative counters such as the number of sexual partners the individual has had, or null values for validation characteristics). An example of a validation characteristic is how CD4 is used in HIV-negative individuals: as the individual is HIV-negative their CD4 is set to -1 at that time; this is used as a validation to test for example that someone initiating ART is HIV-positive, by checking that their CD4 is not -1.

2.4 Death due to natural causes (not AIDS-related)

Individuals get older over time and, if they do not die from AIDS-related illness, they have a chance of dying at each timestep from natural causes at age-specific rates as follows. For each sex and yearly age group we have a natural (non-HIV) mortality rate at that time, derived from UNPD estimates as described in Section 2.4.1 below, and the number of individuals in that group. We draw the number of people in that

group who will die in that timestep $n^a_{death}(t)$ as a binomial random variable. If there are any deaths ($n^a_{death}(t) > 0$) we choose the individuals who will die randomly from that yearly age group. For each individual chosen to die we firstly check that they are in the correct age group and are currently alive, as part of the model error-checking. These individuals are then systematically removed from every relevant scheduling list - for example an HIV-positive individual is removed from the list HIV-related events when not on ART - as no future events can now happen to them. Similarly any scheduled background HIV testing/ART cascade, or scheduled VMMC events are removed for the dead individual. The sexual partners of the dead individual are also updated. The dead individual is removed from the list of potential partners, and the list of susceptibles in serodiscordant partnerships, if they were on those lists. Any individuals who were partners of the deceased individual at time of death have their partnership information updated: we decrease the number of current partners they have by one, update the list of their partners, and add an extra potential partnership from that partner to the list of available partnerships. If the dead individual was HIV-positive and the partner was HIV-negative we update the list of the partner's serodiscordant partners and check if the partner has any other current HIV-positive partners; if they do not then we remove the partner from the list of individuals who are in a serodiscordant partnership.

2.4.1 Deriving mortality rates

The natural (non-HIV) mortality rates used in the model are derived from UNPD WPP estimates in the given country [4]. UNPD provides estimates of the number of deaths that occur in a given 5 year age group (0-4, 5-9,...) during the five year time periods 1950-55, 1955-60, 1960-65,...2010-2015 for each sex. UNPD also publishes the estimated size of each 5 year age group during that period. Projections of future number of deaths and population sizes are also published, and we use the 'medium fertility' variant as described in [7].

UNPD mortality rate estimates include deaths from AIDS-related illness as well as non-HIV mortality. We therefore carry out several steps to derive the natural (non-HIV) mortality rate which is used in PopART-IBM. Firstly we obtain the UNPD annual mortality rate for a given sex g and age group a. We merge all UNPD data from ages 80+ into a single age group for simplicity. As UNPD publishes estimates over 5 year time periods we take this to correspond to mortality at the mid-point of the period, (e.g. at times $t = 1952.5, 1957.5$, etc.). We also divide the UNPD estimate by 5, since we want the annual mortality rate.

For each sex g and age group a we fit a linear regression to the natural log of this mortality rate. HIV-related mortality only affects mortality over a certain time period, which from visual inspection of data is after 1980, and varies by age group. HIV-related mortality is projected to have a more substantial effect for longer in South Africa than in Zambia, while in Zambia future mortality is overestimated if data is restricted to 1950-1980 only. We therefore fit the regression model to the UNPD estimates restricted to the years shown in Table E. Figs A-D show the UNPD values and the estimates from the regression models

Country	Population	Data
Zambia	Men <80	1950-1980 and 2050-2100
Zambia	Men 80+	1950-2100
Zambia	Women <80	1950-1980 and 2050-2100
Zambia	Women 80+	1950-2100
South Africa	Men <80	1950-1980 only
South Africa	Men 80+	1950-2100
South Africa	Women <80	1950-1980 only
South Africa	Women 80+	1950-2100

Table E: Years of UNPD estimates used when fitting regression model to remove effects of HIV-related mortality

The parameters of the regression model are shown in Table F. These parameters are used directly by PopART-IBM to calculate mortality for a given age group of a person a (age group in the 5 year UNPD categories 10-14, 15-19, 20-24 etc.) as:

$$\mu^{a,g} = \exp\left(\zeta^{a,g} + t * \eta^{a,g}\right)$$

where t is the current calendar time, $\zeta^{a,g}$ and $\eta^{a,g}$ are the intercept and slope from the regression model for sex g and age group a in the country. If the calendar time is before 1950, or after 2100, when UNPD estimates are not available, we take $t = 1950$ or $t = 2100$ respectively.

2.5 Ageing

Each individual has a date of birth, stored as part of their characteristics, from which their age can be derived at any given time t. However, for data and algorithmic reasons there are several lists which contain individuals in one year age cohorts (age 13, 14, 15,...79, 80+), or in the 'partnership' age groups: 13-17, 18-22, 23-29, 30-39, 40-49, 50-59, 60-79, 80+. The cohorts are defined by the age at the start of a calendar year. Note that individuals only enter the adult population aged 13.99 years, and therefore the one year age group for 13 year olds only contains individuals who have entered the adult population at this time; the remaining 13 year olds are stored in the list of children. Similarly the 13-17 year old age group only includes those 13 years and above who are 13.99 years or older at present.

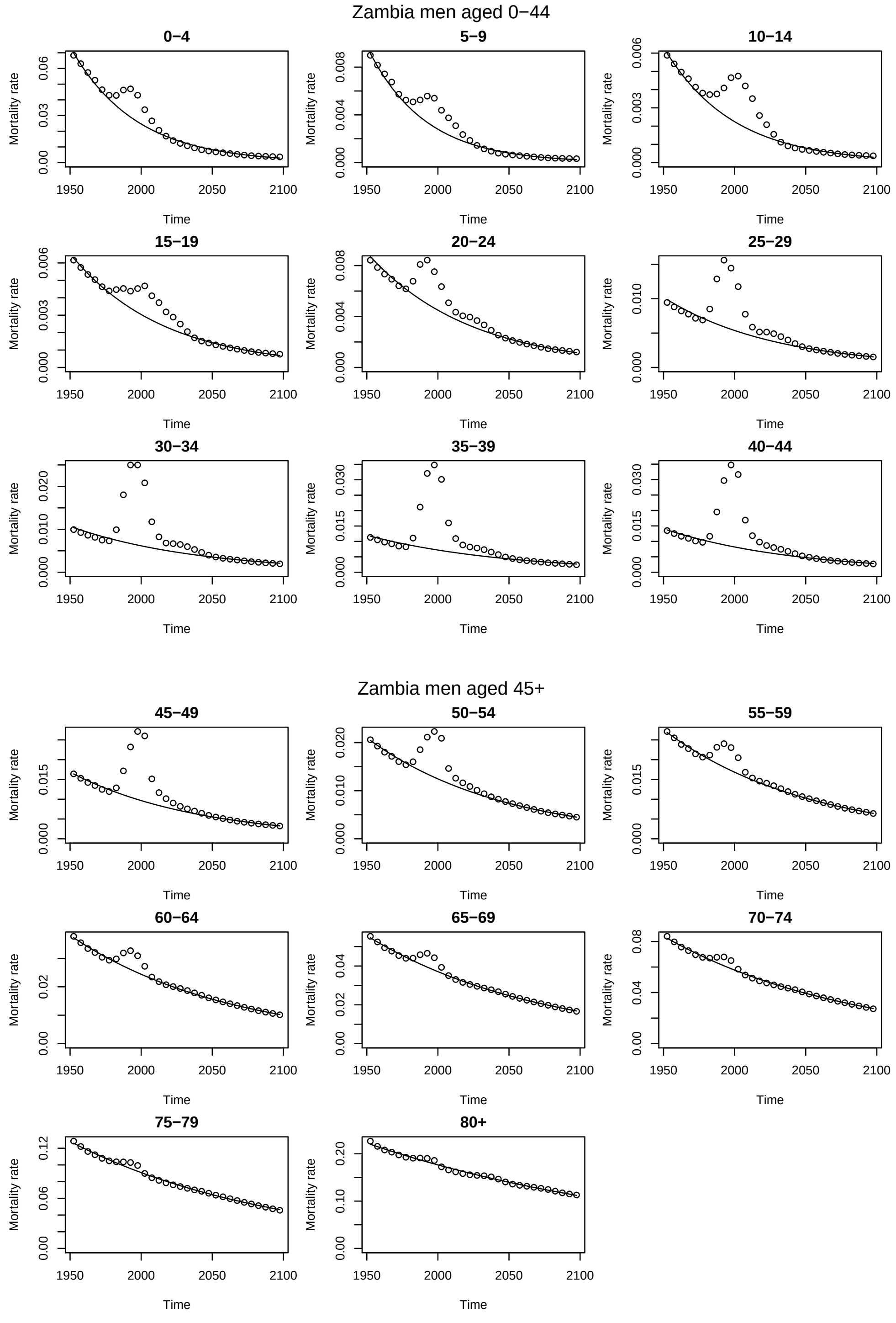

Fig A: Annual mortality for men in Zambia by age group. Circles show UNPD estimates of mortality including HIV-related mortality. Lines show fitted regression estimates of non-HIV mortality as used in PopART-IBM.

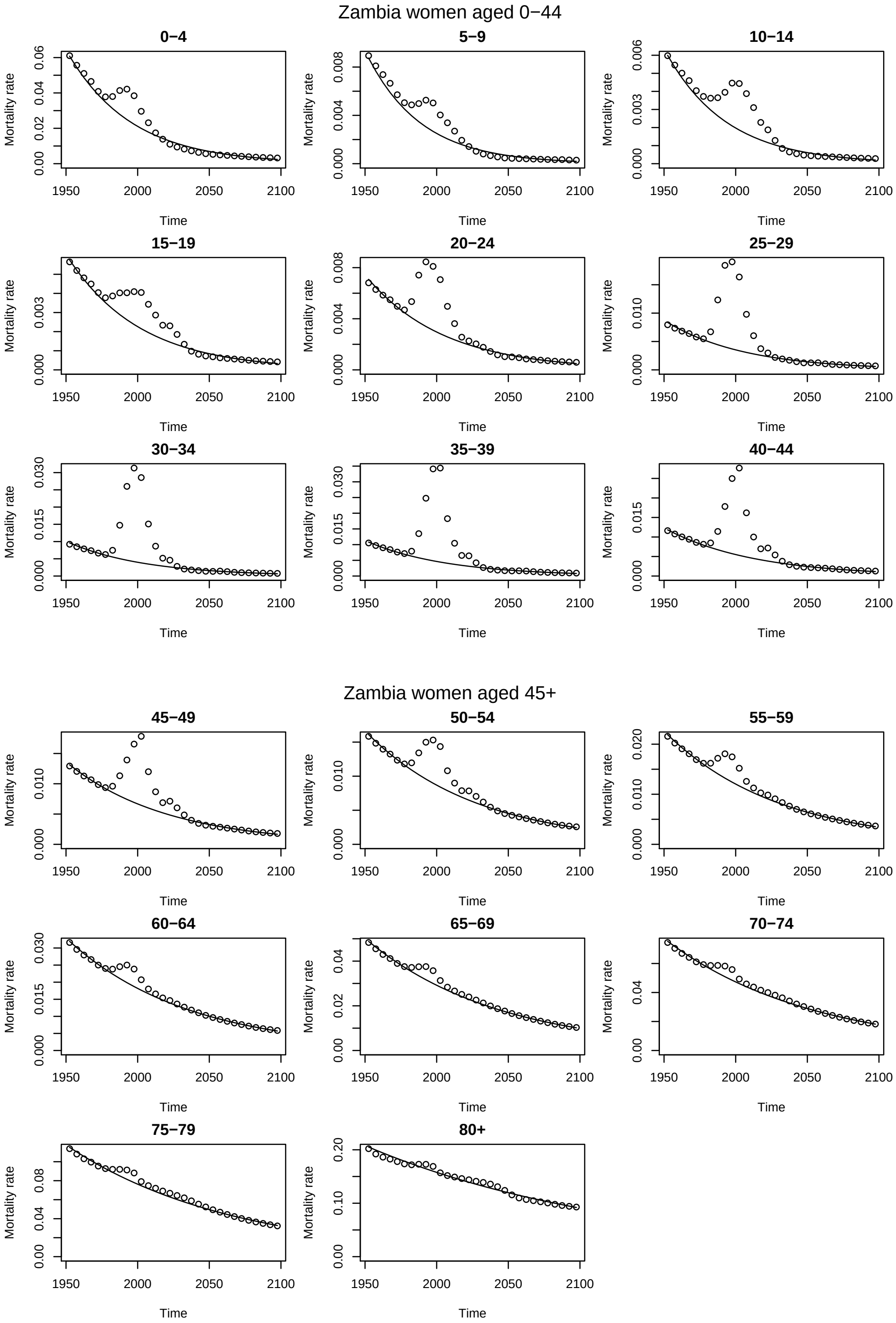

Fig B: Annual mortality for women in Zambia by age group. Circles show UNPD estimates of mortality including HIV-related mortality. Lines show fitted regression estimates of non-HIV mortality as used in PopART-IBM.

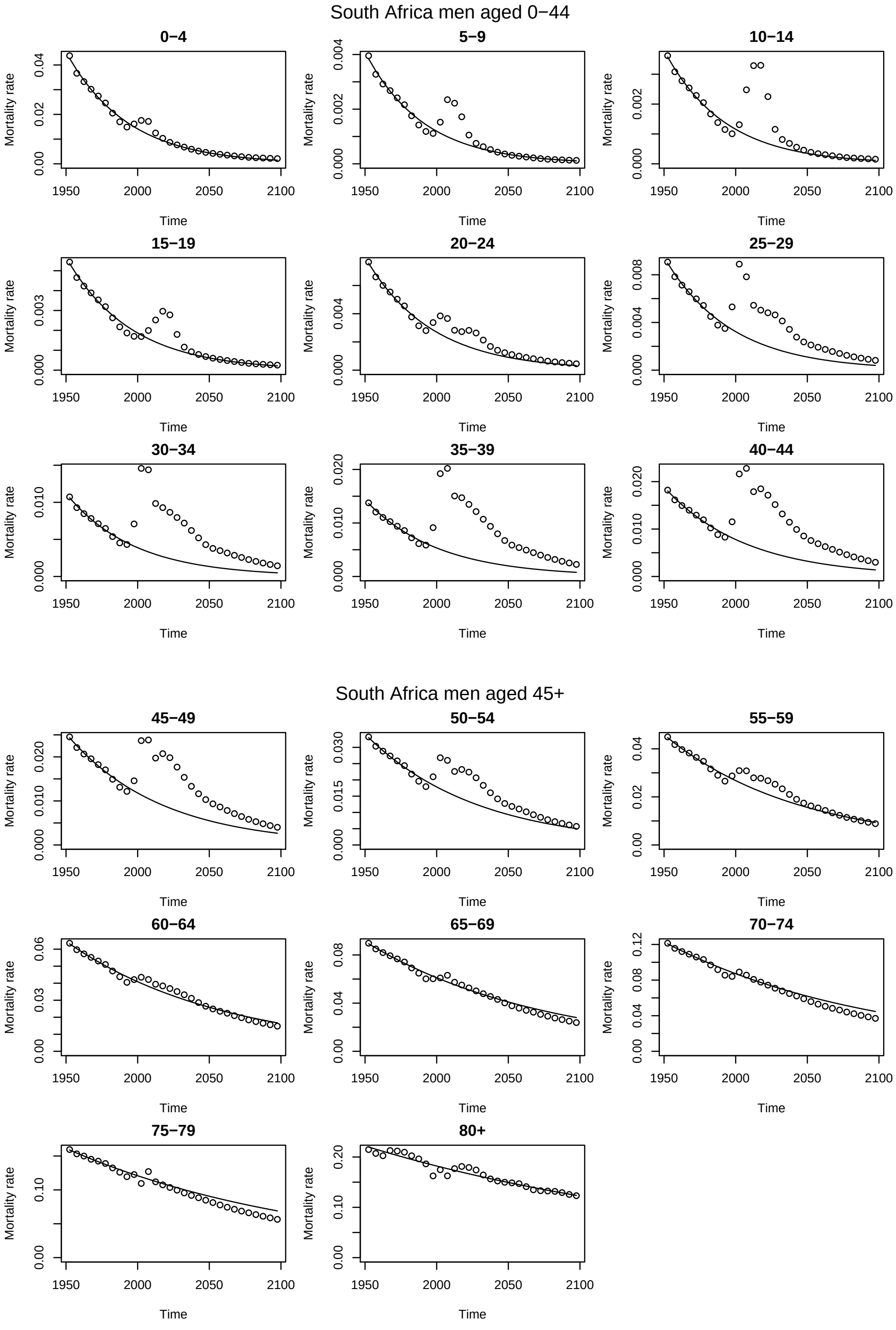

Fig C: Annual mortality for men in South Africa by age group. Circles show UNPD estimates of mortality including HIV-related mortality. Lines show fitted regression estimates of non-HIV mortality as used in PopART-IBM.

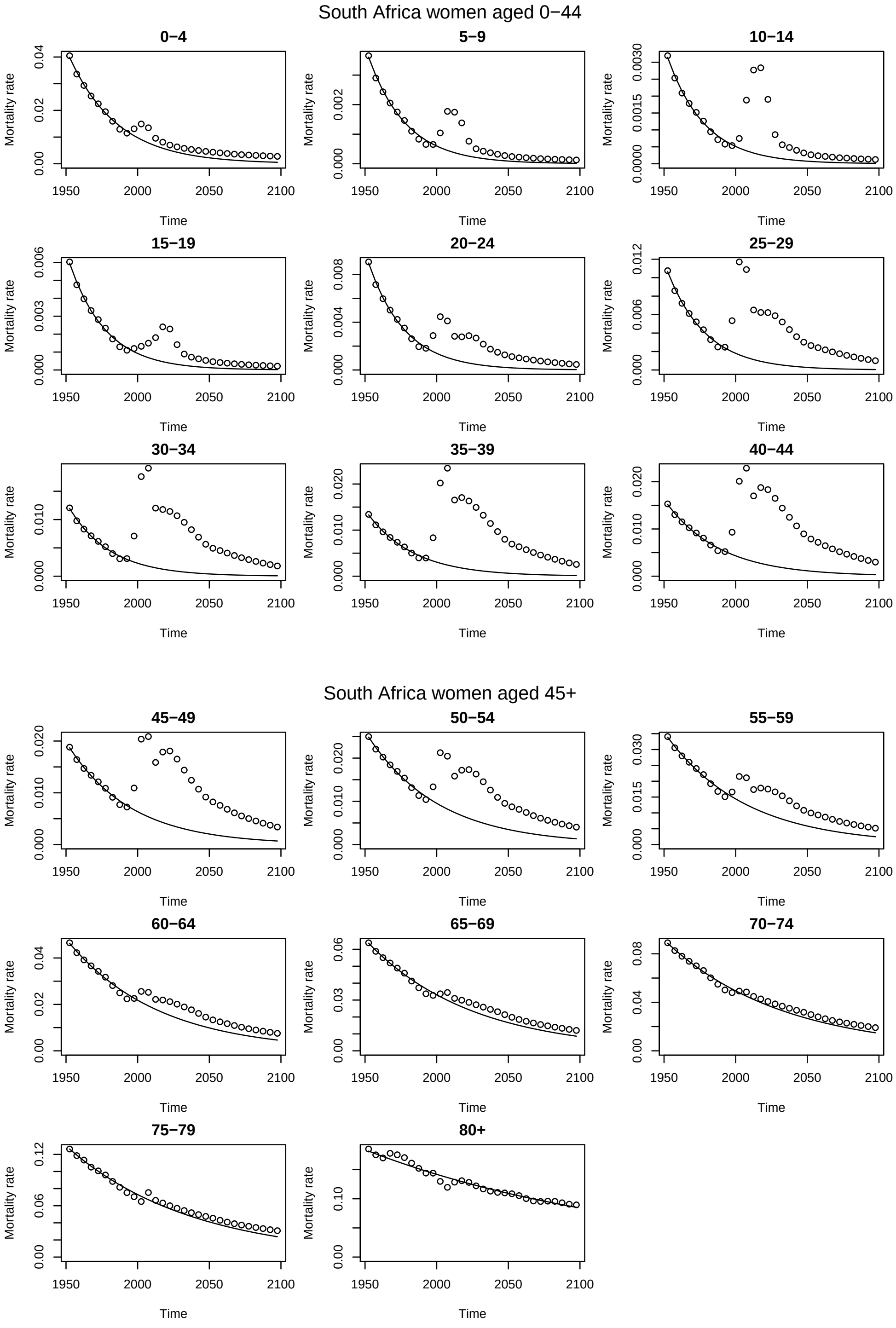

Fig D: Annual mortality for women in South Africa by age group. Circles show UNPD estimates of mortality including HIV-related mortality. Lines show fitted regression estimates of non-HIV mortality as used in PopART-IBM.

	Zambia				South Africa			
	Men		Women		Men		Women	
**Age group**	Intercept	Slope	Intercept	Slope	Intercept	Slope	Intercept	Slope
0-4	40.212	-0.022	40.705	-0.022	42.293	-0.023	54.690	-0.030
5-9	44.032	-0.025	46.709	-0.026	42.586	-0.025	68.215	-0.038
10-14	35.333	-0.021	40.583	-0.023	40.871	-0.024	69.069	-0.038
15-19	24.761	-0.015	33.245	-0.020	38.144	-0.022	71.719	-0.039
20-24	22.432	-0.014	30.933	-0.018	37.628	-0.022	71.849	-0.039
25-29	20.435	-0.013	30.743	-0.018	37.291	-0.022	68.580	-0.037
30-34	17.019	-0.011	31.502	-0.019	36.825	-0.021	63.583	-0.035
35-39	15.467	-0.010	30.226	-0.018	34.332	-0.020	56.239	-0.031
40-44	16.937	-0.011	27.039	-0.016	30.516	-0.018	47.610	-0.027
45-49	17.651	-0.011	23.513	-0.014	26.053	-0.015	40.423	-0.023
50-54	16.497	-0.010	21.266	-0.013	21.867	-0.013	35.665	-0.020
55-59	15.642	-0.010	20.786	-0.013	17.916	-0.011	31.795	-0.018
60-64	14.097	-0.009	19.762	-0.012	15.110	-0.009	27.901	-0.016
65-69	12.925	-0.008	18.136	-0.011	13.182	-0.008	24.094	-0.014
70-74	12.310	-0.008	16.554	-0.010	11.254	-0.007	21.628	-0.012
75-79	11.378	-0.007	14.728	-0.009	9.395	-0.006	20.417	-0.012
80+	7.562	-0.005	9.202	-0.006	6.229	-0.004	8.480	-0.005

Table F: Country, sex and age-group specific mortality parameters.

For computational efficiency ageing in the one year age cohorts is accomplished at the start of the year by relabelling the cohorts, so that 14 becomes 15, etc. Individuals in the age 79 cohort are moved into the 80+ cohort. In terms of the model code, the array previously containing the age 79 cohort is now the age 14 cohort and is emptied until new individuals enter the adult population. The same relabelling technique is used for other objects storing data in one year age groups such as population size.

In the partnership age groups we use the one year age cohort to list all the individuals who are about to transition from one partnership age group to the next age group. These individuals are moved in a multi-step process. Firstly an individual is added to the end of the next age group list. Then the previous age group list is updated: the last individual on the previous list is moved into the position that beloned to the individual who is moving age groups. Finally the number of individuals in each age group is updated, reducing the number in the previous age group by one and increasing the number in the new age group by one. This is repeated until all individuals have been moved.

2.6 Sex ratio

The sex ratio in the population is primarily governed by the parameter $f^{sex=M}$ which determines the proportion of new adults who are male. The sex ratio in the population is also affected by mortality, both non-HIV related and HIV related, as these vary by the sex of the individual. The value for the parameter $f^{sex=M}$ used is the country-level sex ratio from [6], and is given in Table B.

3 Partnerships

3.1 Overview

Within PopART-IBM we explicitly model heterosexual partnerships: in other words individuals form and dissolve (break up) sexual partnerships with other individuals of the opposite sex. Prior ranges and values for partnership parameters come from an analysis of data from PC0.

Partnership formation is governed by an age-mixing matrix. As mentioned previously, there are three levels of sexual activity: low, medium or high, where higher activity reflects a higher propensity to form partnerships (Section 3.2). We assume independence between the effects of age and sexual activity level on the rate of partnership formation, and partnership formation by sexual activity level is determined by an assortativity parameter χ which is calibrated. A proportion of partnerships (fixed across sex, sexual activity level and age) are assumed to be formed within the same patch (i.e. community), and the remaining with partners outside that patch.

In the PC0 survey, women report lower partnership formation rates than men. To account for this, within the model we assume that partnership formation rates can be over-reported by men, underreported by women, or a combination of both (see Section 3.3.2 for more details).

In this section we describe the theory behind the approach used in PopART-IBM, as well as the way that partnerships are modelled in PopART-IBM, and the analysis of the baseline HPTN 071 (PopART) population cohort survey (PC0) that parameterises the partnership processes (sexual partnership formation and breakup) in PopART-IBM. A full list of partnership-related parameters is given in Table G.

3.1.1 Parameter estimation using the PC0 data

We use data from the baseline population cohort (PC0) of the HPTN 071 (PopART) trial to derive the parameters related to partnership formation and dissolution used in PopART-IBM. PC0 contains a number of questions on sexual behaviour, among other topics. All PC0 participants were asked basic questions about sexual activity such as the number of partners they had in the past year. A subset of the survey population was given an extended questionnaire (*"Extended PC0"*) containing additional questions on sexual behaviour. Those individuals were asked detailed information about their up to three most recent sexual partners in the last year (note that an individual who had only one partner in the last year would therefore respond only about that partner regardless of how many lifetime partners they had had).

Note that parameters on sexual behaviour are estimated at the country level (i.e. using participants from Zambia or South Africa respectively) in order to have a large enough sample: in some cases parameters were stratified by sex, age and also sexual activity level; in others the analysis was based on data from the Extended PC0 questionnaire, which only a subset of participants completed.

Parameter	Value		Notes
	Zambia	South Africa	
χ, risk assortativity	0.05-0.95		Large range to reflect uncertainty.
θ, proportion of compromise from males	0.01-0.5		Assumption that women underreport more than men.
$c_a^{m,in}$, within community partnership formation rates for men			
13-17	0.0174 yr^{-1}	0.0506 yr^{-1}	No PC data. Assumed 50% less than 18-22.
18-22	0.0348 yr^{-1}	0.1011 yr^{-1}	PC0 analysis
23-29	0.0409 yr^{-1}	0.1615 yr^{-1}	PC0 analysis
30-39	0.0251 yr^{-1}	0.0646 yr^{-1}	PC0 analysis
40-49	0.0243 yr^{-1}	0.0505 yr^{-1}	PC0 analysis
50-59	0.0051 yr^{-1}	0.0152 yr^{-1}	No PC data available, assume 50% decline
60-79	0.0025 yr^{-1}	0.0076 yr^{-1}	in each age group.
$c_a^{f,in}$, within community partnership formation rates for women			
13-17	0.0089 yr^{-1}	0.0260 yr^{-1}	No PC data. Assumed 50% less than 18-22.
18-22	0.0177 yr^{-1}	0.0520 yr^{-1}	PC0 analysis
23-29	0.0147 yr^{-1}	0.0286 yr^{-1}	PC0 analysis
30-39	0.0102 yr^{-1}	0.0303 yr^{-1}	PC0 analysis
40-49	0.0131 yr^{-1}	0.0391 yr^{-1}	PC0 analysis
50-59	0.0065 yr^{-1}	0.0196 yr^{-1}	No PC data available, assume 50% decline
60-79	0.0033 yr^{-1}	0.0098 yr^{-1}	in each age group.
Relative number of partnerships by risk group			
Low risk δ^{low}	1.00	1.00	
Medium risk δ^{med}	8.89	2.06	
High risk δ^{high}	22.4	6.38	
$c_{multiplier}$, multiplier to account for mis-reporting of number of sexual partners	0.5-4.0	0.5-4.0	1.0 means that people report the correct number of partners, > 1 means people are under-reporting. Lower range taken to ensure 1 is sampled well and to allow over-reporting.
Relative rate of formation of partnerships between patches compared to within patches	0.562	0.665	PC0 analysis.
Unscaled duration of low risk partnerships within patch	15.4 years	9.80 years	PC0 data
Unscaled duration of medium risk partnerships within patch	6.1 years	6.99 years	PC0 data
Unscaled duration of high risk partnerships within patch	3.8 years	4.16 years	PC0 data
Multiplier scaling duration of all partnerships	1.0-2.0	1.0-2.0	Assumption
Multiplier scaling duration of partnerships between patches compared to within patch	0.456	0.575	PC0 data
Maximum number of concurrent partners by risk group:			
Low	1		Assumption - over 90% of PC have 0 or 1 partners in last year.
Medium	3		Assumption.
High	10		Assumption.

Table G: Partnership-related parameters used in PopART-IBM.

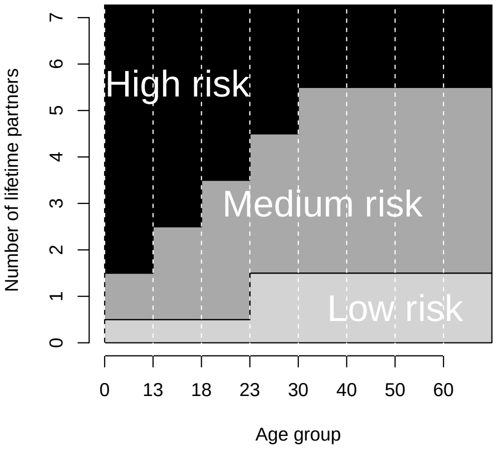

Fig E: Definition of sexual activity levels in PopART-IBM according to age and lifetime number of partners.

3.2 Sexual activity level

Here we describe the sexual activity levels used in PopART-IBM. We first describe how they are defined based on PC0 data, and then we describe how sexual activity level affects individuals within PopART-IBM.

3.2.1 Defining activity levels using PC0 data

The sexual activity levels used in PopART-IBM are defined based on threshold number of lifetime partners by age groups reported by individuals in the PC0 survey. In the PC0 survey, for each age group, we chose thresholds as shown in Fig E to maximise differences in prevalence between different activity classes. Fig F shows that prevalence for men and women in PC0 is indeed higher in the higher sexual activity levels, even when accounting for age. These thresholds were then used to stratify the PC0 data for analysis by sexual activity level. The proportion of individuals in each activity class, as observed in PC0, is given in Table H. Note that the thresholds are not applied in PopART-IBM directly - instead individuals in a given age group and sexual activity level have partnership formation rates coming from the analysis of PC0 for that age group and sexual activity level.

As described in Section 2.3, the sexual activity level to which an individual belongs in PopART-IBM is determined at entry to adulthood and is constant for life.

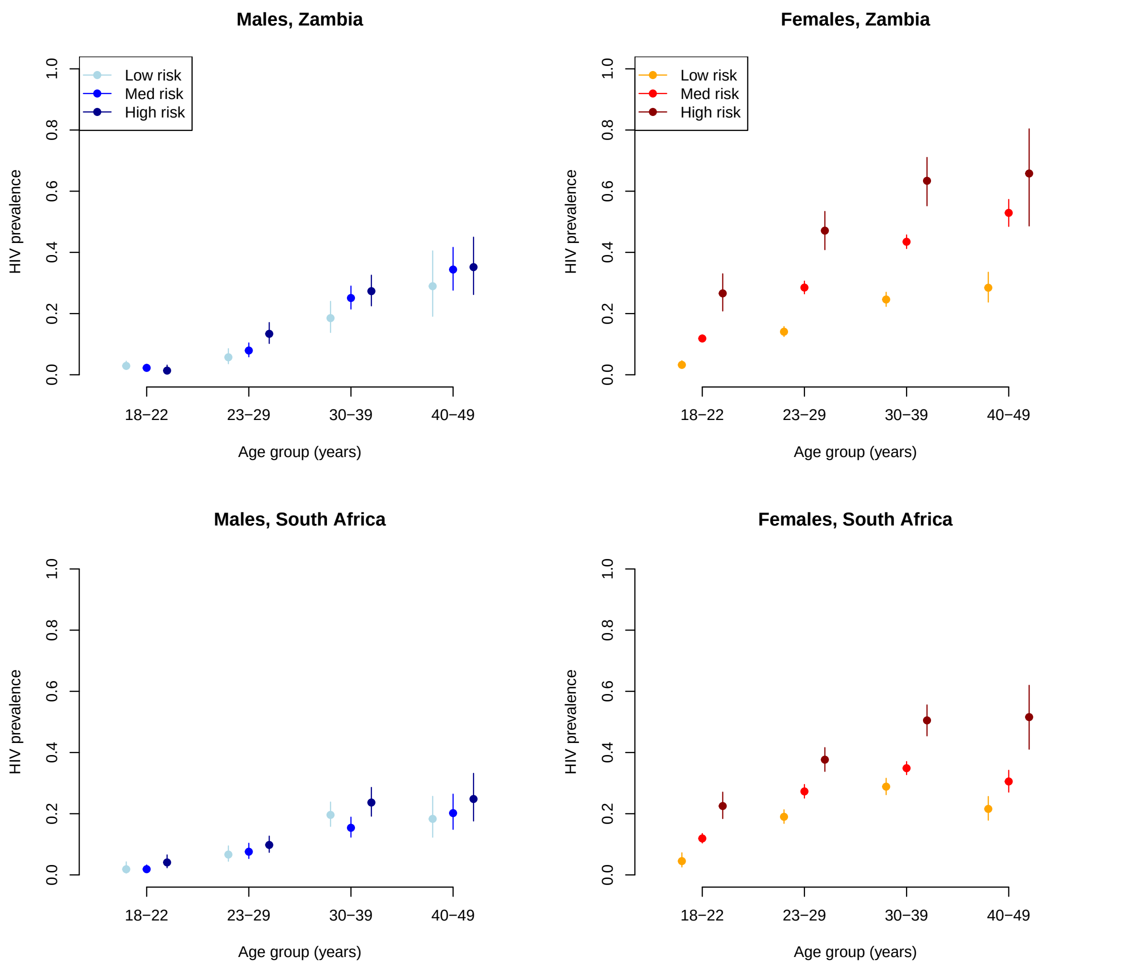

Fig F: HIV prevalence by sex, sexual activity level and age group for Zambia (top row) and South Africa (bottom row) in PC0 data.

3.2.2 Maximum number of simultaneous partners

We assume that individuals in PopART-IBM cannot have more than a certain number of partners at any given time. This maximum number of partners is assumed to depend on the activity class and to be higher in the higher activity classes. For each individual, formation of new partnerships is independent of the current number of partners, but with the constraint that they cannot exceed the maximum number of partners at any given time.

We take the maximum number of partners to be 1 (low), 3 (medium) and 10 (high), informed by PC0 data. In a sensitivity analysis (not shown) we allowed each of these to increase by 1 (i.e. 2 (low); 4 (medium); 11 (high)), with no effect on results.

	% low activity	% medium activity	% high activity
Females, Zambia	35.50	59.30	5.21
Males, Zambia	28.60	46.90	24.60
Females, South Africa	29.60	56.40	14.00
Males, South Africa	27.70	41.50	30.80

Table H: Proportion of population in each sexual activity level by sex from PC0 survey data.

3.3 New partnership formation in PopART-IBM

Within PopART-IBM at each timestep we first choose all the partnerships to be formed, and then we form each partnership in turn, updating each partner's characteristics to reflect the newly formed partnership. In this section we describe how the partnerships to be formed are chosen.

In PopART-IBM, based on initial analysis of PC0, the rates of partnership formation and dissolution are dependent on age (and sex for partnership formation) as well as the level of sexual activity level of the individual r, where higher activity reflects a higher propensity to form partnerships.

We assume independence between the effects of age and sexual activity level on the rate of partnership formation. Partners are chosen according to an age-mixing matrix (Section 3.3.1), while the extent to which individuals prefer other individuals with the same level of inherent sexual activity is determined by an assortativity parameter χ which is calibrated ($\chi = 0$ represents proportionate mixing; $\chi = 1$ is fully assortative mixing; see Section 3.3.3). A proportion of partnerships (fixed across sex, sexual activity level and age) are assumed to be formed within the same patch (i.e. community), and the remaining with partners outside that patch (Section 3.3.4).

In the PC0 survey, women report lower partnership formation rates than men; to account for this, within the model a compromise parameter θ (Section 3.3.2) governs the extent to which actual partnership formation rates reflect these two reported rates - in other words we assume that partnership formation rates can be over-reported by men, underreported by women, or a combination of both. We firstly outline the mathematics behind partner formation: how partnerships balance between age groups and how this under/over-reporting by sex is accounted for (Section 3.3.2), followed by how sexual activity level is also incorporated (Section 3.3.3). In Section 3.5 we describe the mechanics of partnership formation between two individuals.

3.3.1 Age Assortativity

Tables I, J, K and L present the age mixing matrices p_{age} by sex for Zambia and South Africa, obtained by considering the age of the last reported up to three partners (within the last year) of each individual surveyed in the Extended PC0 questionnaire. In younger (respectively older) age groups, where no data was available in PC0, we assumed age mixing would be similar to that of the age group immediately older (respectively younger),

	13-17	18-22	23-29	30-39	40-49	50-59	60+
13-17	0.92	0.08	0.00	0.00	0.00	0.00	0.00
18-22	0.38	0.54	0.08	0.00	0.00	0.00	0.00
23-29	0.07	0.59	0.31	0.03	0.00	0.00	0.00
30-39	0.01	0.10	0.49	0.38	0.03	0.00	0.00
40-49	0.00	0.01	0.04	0.63	0.28	0.04	0.00
59-59	0.00	0.00	0.01	0.04	0.63	0.28	0.04
60+	0.00	0.00	0.00	0.01	0.04	0.63	0.32

Table I: Age mixing matrix for men in Zambia. Each row i shows $p^{m}_{age}[a, .]$, that is, for a surveyed man in age group a, the age distribution of the reported partners.

	13-17	18-22	23-29	30-39	40-49	50-59	60+
13-17	0.15	0.61	0.05	0.09	0.11	0.00	0.00
18-22	0.01	0.14	0.61	0.05	0.09	0.11	0.00
23-29	0.01	0.01	0.26	0.55	0.13	0.06	0.00
30-39	0.00	0.00	0.01	0.37	0.51	0.05	0.06
40-49	0.01	0.00	0.00	0.04	0.52	0.25	0.19
50-59	0.00	0.01	0.00	0.00	0.04	0.52	0.44
60+	0.00	0.00	0.01	0.00	0.00	0.04	0.96

Table J: Age mixing matrix for women in Zambia. Each row i shows $p^{f}_{age}[a, .]$, that is, for a surveyed woman in age group a, the age distribution of the reported partners.

but with age categories shifted accordingly.

3.3.2 Mathematical approach to balancing the number of partnerships formed between age groups

Within an individual-based model, the process of generating a sexual network is complex. While preferences for mixing by age in partnerships is specified by the age mixing matrix in Section 3.3.1, the actual number of partnerships formed between age groups must balance. We present here the theoretical underpinning of partnership formation process according to sex and age used in PopART-IBM. For simplicity, we initially ignore sexual activity level in this section, and introduce this additional level of complexity in Section 3.3.3. Throughout we use a (and a^*) to denote age groups as used by PopART-IBM (13-17, 18-22, 23-29, 30-39, 40-49, 50-59, 60-79, 80+), and the term 'aged a' should be understood as 'in age group a'.

Let c^f (respectively c^m) be a vector so that c^f_a (respectively c^m_a) is the average number of new partners per year as reported by a female (respectively a male) aged a (note that these are the partnership formation rates as derived directly from analysis of PC0 data; as explained above, women tend to report lower rates than men, so as we describe shortly we use a parameter θ to adjust these rates within the model itself - in other words the adjustment is done during the simulation).

	13-17	18-22	23-29	30-39	40-49	50-59	60+
13-17	0.89	0.04	0.07	0.00	0.00	0.00	0.00
18-22	0.22	0.67	0.04	0.07	0.00	0.00	0.00
23-29	0.03	0.49	0.34	0.14	0.00	0.00	0.00
30-39	0.01	0.08	0.43	0.35	0.06	0.00	1.00
40-49	0.01	0.00	0.11	0.46	0.32	0.09	0.00
50-59	0.00	0.01	0.00	0.11	0.46	0.32	0.09
60+	0.00	0.00	0.01	0.00	0.11	0.46	0.42

Table K: Age mixing matrix for men in South Africa. Each row i shows $p^m_{age}[a,.]$, that is, for a surveyed man in age group a, the age distribution of the reported partners.

	13-17	18-22	23-29	30-39	40-49	50-59	60+
13-17	0.29	0.54	0.00	0.18	0.00	0.00	0.00
18-22	0.01	0.28	0.54	0.00	0.18	0.00	0.00
23-29	0.01	0.01	0.39	0.37	0.14	0.08	0.00
30-39	0.00	0.00	0.04	0.41	0.47	0.07	0.00
40-49	0.01	0.00	0.00	0.10	0.61	0.10	0.19
50-59	0.00	0.01	0.00	0.00	0.10	0.61	0.28
60+	0.00	0.00	0.01	0.00	0.00	0.10	0.90

Table L: Age mixing matrix for women in South Africa. Each row i shows $p^f_{age}[a,.]$, that is, for a surveyed woman in age group a, the age distribution of the reported partners.

Let p^f_{age} be a matrix so that $p^f_{age}[a,.]$ is the distribution over age of "desired" male sexual partners of a female aged a. Similarly, let p^m_{age} be a matrix so that $p^m_{age}[a,.]$ is the distribution over age of "desired" female sexual partners of a male aged a.

Let $\mathbf{N^f}(t)$ and $\mathbf{N^m}(t)$ be two vectors containing the population size of each age group within females and males respectively, which may vary over time. The elements of, for example, $\mathbf{N^f}(t)$ are then denoted $N^f_a(t)$, the number of females aged a at time t.

At each timestep in PopART-IBM, we compute the number of new partnerships that the group of females of age a desire to form in this timestep with males of age a^* as $S^f_{a,a^*}(t) = N^f_a(t)\, c^f_a p^f_{age}[a,a^*]dt$, where dt is the duration of a timestep in the model (=1/48 year). Similarly, we compute the number of partnerships that the group of males of age a^* desire to form in this timestep with females of age a as $S^m_{a^*,a}(t) = N^m_{a^*}(t)\, c^m_{a^*} p^m_{age}[a^*,a]dt$.

Let $T^f_{a,a^*}(t)$ and $T^m_{a^*,a}(t)$ be the number of actual partnerships formed between females aged a and males aged a^*, and males aged a^* and females aged a respectively between t and $t+dt$. In order for partnerships to be balanced, we need

$$T^f_{a,a^*}(t) = T^m_{a^*,a}(t) \qquad \forall (a,a^*).$$

Garnett and Anderson [8] proposed that to achieve this, one should take the geometric mean between the number of partnerships as desired by females and as desired by males,

weighted by a compromise parameter, varied between 0 and 1 depending on the extent to which men or women made compromises.

Here we argue that an equal compromise would be that half of the unmatched partnerships (desired by one sex but not by the other) are satisfied. This corresponds to taking the arithmetic instead of the geometric mean, weighted by a compromise parameter θ with, again, $\theta = 1$ if only women make compromises, $\theta = 0$ if only men make compromises, and $\theta = 0.5$ if men and women make equal compromises. Unlike the geometric mean approach, this approach does not tend to favour the group that desire fewer partners.

Therefore, at each timestep, the actual number of partnerships to be formed between females aged a and males aged a^* (ignoring sexual activity levels) is

$$T^f_{a,a^*}(t) = (1-\theta)\, S^f_{a,a^*}(t) + \theta S^m_{a^*,a}(t) .$$

We generalize this to reflect the fact that there are different sexual activity levels as described in Section 3.3.3 below.

Note that in this approach we assume that, for an individual of a given age and sex, the desired number and ages of new partners are constant over time (as p^f_{age}, p^m_{age}, c^f and c^m are taken to be constant over time) but the actual number and ages of new partners is limited by the availability of individuals in each age group (since $\mathbf{N^f}$ and $\mathbf{N^m}$ both vary over time).

3.3.3 Mathematical approach to balancing the number of partnerships formed between sexual activity levels

As described before, we assume that individuals are divided into sexual activity levels corresponding to different propensities to form partnerships (the low, medium, high activity levels described previously - see Section 3.2.1 for how these levels are defined from analysis of PC0 data) and that each individual remains in the same activity level for their whole life (although it should be noted that the rate of acquisition of new partners changes for an individual as they age). We also assume, for parsimony, that the age and sexual activity level preferences in forming partnerships are independent.

We denote $c^{f,r}$ (respectively $c^{m,r}$) the rate of partnership formation of females (respectively males) in activity level r. We further assume that the relative rate of partnership formation according to activity level is the same in males and females so that:

$$\begin{aligned} c^{f,high} &= \delta^{high} c^{f,low}; \quad & c^{f,med} &= \delta^{med} c^{f,low}; \quad & c^{f,low} &= \delta^{low} c^{f,low} \\ c^{m,high} &= \delta^{high} c^{m,low}; \quad & c^{m,med} &= \delta^{med} c^{m,low}; \quad & c^{m,low} &= \delta^{low} c^{m,low} \end{aligned}$$

We fix $\delta^{low} = 1$ by convention, so that all the rates of partnership formations are written relative to the low activity class.

Let $p^f_{risk}(t)$ be a time-varying matrix (time-varying as the proportion of individuals in each sexual activity level can vary over time) so that $p^f_{risk}[r,.](t)$ is the distribution

over activity levels of desired male sexual partners of a female of sexual activity level r at time t. Similarly, let $p^m_{risk}(t)$ be a matrix so that $p^m_{risk}[r,.](t)$ is the distribution over activity levels of desired female sexual partners of a male of sexual activity level r at time t. We assume the elements of these matrices are of the form (given here for females but the same formula, after swapping sex, applies for males):

$$p^f_{risk}[r,r^*](t) = \begin{cases} \chi + (1-\chi) P^m_r(t) & r = r^* \\ (1-\chi) P^m_{r^*}(t) & r \neq r^* \end{cases}$$

where $P^m_r(t) = \sum\limits_{a=1}^{n_{age}} N^m_{a,r}(t) \,/\, \sum\limits_{s=1}^{n_{risk}} \sum\limits_{a=1}^{n_{age}} N^m_{a,s}(t)$ is the proportion of the male population in activity level r at time t (with $N^m_{a,r}(t)$ and $N^f_{a,r}(t)$ the number of males and females in age group a and activity level r at time t).

In this formulation, χ is the assortativity, i.e. the proportion of partnerships formed preferentially within the same activity level (as opposed to random partnerships formed with any activity level). χ is a constant which is calibrated.

The number of partnerships that women aged a in activity class r "want" to make, between t and $t+dt$, with men aged a^* in activity class r^* is:

$$S^f_{(a,r),(a^*,r^*)}(t) = N^f_{a,r}(t)\, c^{f,low}_a \delta^r p^f_{age}[a,a^*] p^f_{risk}[r,r^*](t)\, dt \tag{1}$$

And the number of partnerships that men aged a^* in activity class r^* "want" to make, each year, with women aged a in activity class r is:

$$S^m_{(a^*,r^*),(a,r)}(t) = N^m_{a^*,r^*}(t)\, c^{m,low}_{a^*} \delta^{r^*} p^m_{age}[a^*,a] p^m_{risk}[r^*,r](t)\, dt \tag{2}$$

As in Section 3.3.2, if the "desired" number of partnerships are not the same for both sexes (and they will almost certainly not be, both because of the previously mentioned over/underreporting in PC0 data and because the "desired" number depends on the number of people in a given sex/age/sexual activity group which has stochastic variability coming from the demographic component of PopART-IBM), we compute an adjusted number of partnerships, calculated as the weighted arithmetic mean between the two. This is, as before:

$$T^f_{(a,r),(a^*,r^*)}(t) = (1-\theta)\, S^f_{(a,r),(a^*,r^*)}(t) + \theta S^m_{(a^*,r^*),(a,r)}(t) \tag{3}$$

3.3.4 Partners inside and outside the community

We assume that individuals form a proportion of their new partnerships with partners in their own patch (community), and the rest in the other patch ('outside the community'). Mixing by sexual activity level and age is assumed to be exactly the same with partners inside and outside the community. In practice, we assume that the within-patch partnership formation rates are $c^{f,in}_a$ for females and $c^{m,in}_a$ for males aged a. We then assume that the between-patch partnership formation rates are $c^{f,out}_a = \xi c^{f,in}_a$ for females and $c^{m,out}_a = \xi c^{m,in}_a$ for males aged a. This is equivalent to assume that a proportion $\frac{\xi}{1+\xi}$ of

partnerships formed by both males and females (of all ages) are formed with partners outside their patch.

Within the model, the risk of HIV transmission within a serodiscordant partnership depends on a number of characteristics of the two individuals, described in detail in Section 4.2 on HIV transmission, but it also depends on whether individuals are in the same patch or in different patch; the risk of HIV transmission is lower when partners are not in the same patch, to reflect the lower frequency of unprotected sex reported in the PC0 survey when the partner lives outside the community.

3.3.5 Estimating the rate of partnership formation from PC0 by sex, age and sexual activity level

To estimate the rate of partnership formation, we considered the reported date of first sex with the up to last three partners of all individuals who were administered the Extended PC0 questionnaire, and examined whether it was more or less than a year before date of the PC0 interview. Based on this, for each individual, we classified their up to last 3 partners as "new" (first sex occurred in the last year) or "not new". For individuals who reported more than 3 partners, the proportion of the additional partners who were "new" was imputed based on the proportion of new partners among the last 3, assuming that these 3 were representative of all partners. For the last up to 3 partners, individuals also reported whether these partners lived inside or outside the community. We assumed the new partners among these last 3 were representative of potential additional new partners, so inferred (deterministically) the location of these extra new partners, when needed, based on the reported location of the new partners among the last 3 partners. This allowed us to estimate the number of "new" partnerships initiated by each individual in the last year both inside and outside the community.

We then used this data to estimate, using a Poisson likelihood, the parameters of our partnership formation model. These parameters are $c_a^{f,in}$ and $c_a^{m,in}$ (the rates at which low-activity females and males aged a form partnerships with partners inside their community respectively), ξ (the relative rate of partnership formation with partners outside versus inside the community), and δ^{med} and δ^{high} (the relative rate of partnership formation for medium and high activity individuals). We compared our model to an 'unconstrained' model where rates of partnership formation inside / outside the community and in each sex/age/activity level are estimated completely independently, without assuming multiplying effects as we have done in our 'constrained' model. The results of this comparison are shown in Figs G and H. Despite the additional complexity of the unconstrained mode the estimates were similar, suggesting that the original model captures the trends in the data by age, sex and activity level well. Values for the parameters are given in Table G.

3.4 Allowing flexibility around reported behaviours

To allow some flexibility around parameters estimated directly from reported data on sexual behaviours we used two 'multiplier' parameters; one for the rate of partnership

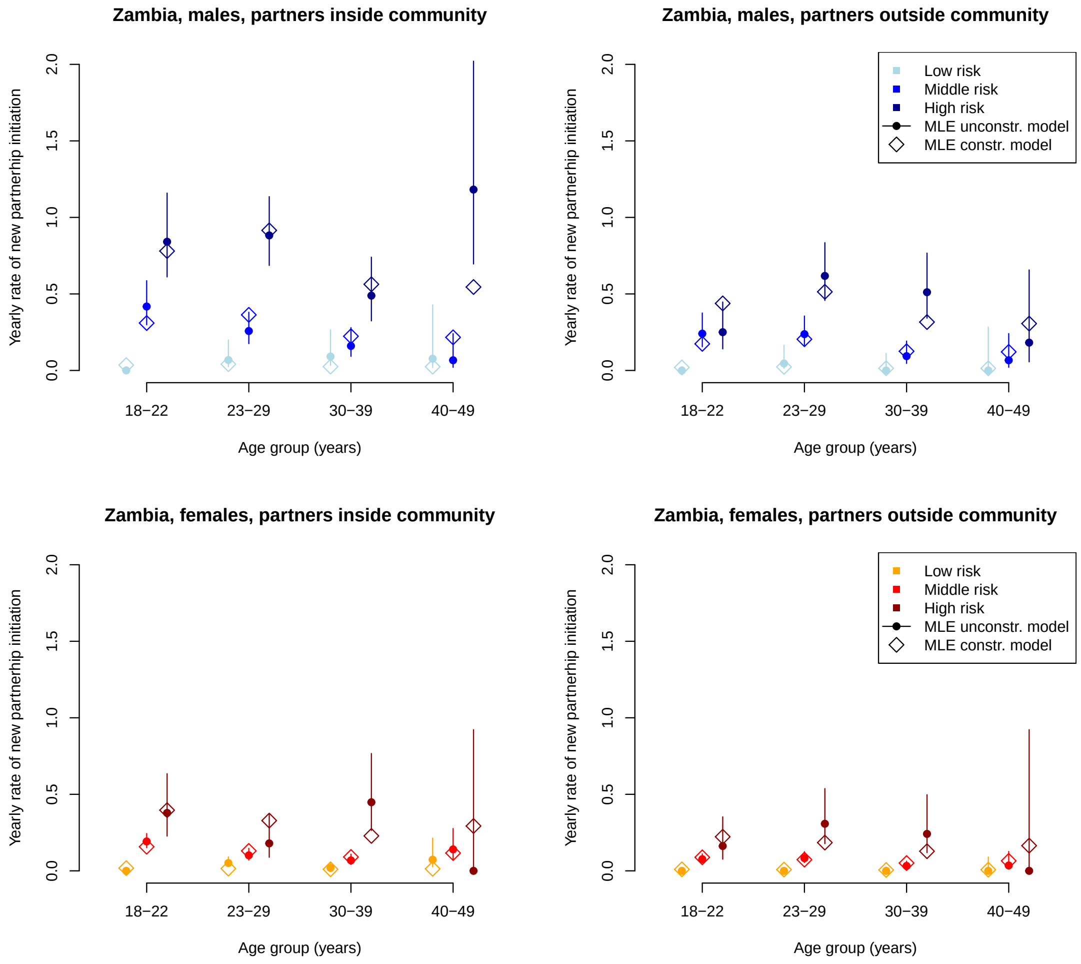

Fig G: Rate of partnership formation inside (left) and outside (right) the community, by age, activity class and sex for Zambia. Age groups not shown had too few observations to provide a reliable estimate. The diamonds correspond to the maximum likelihood estimates of the rates in our model, where the effects of age, activity class and location (inside/outside) are modelled using multiplying effects on the partnership formation rate (see text for details). The dots and confidence intervals correspond to the likelihood for the unconstrained model where rates of partnership formation inside / outside the community and in each sex/age/activity class are estimated completely independently.

formation ($c_{multiplier}$), and one for the mean duration of partnerships. Table G shows the prior range for each parameter used in the calibration process.

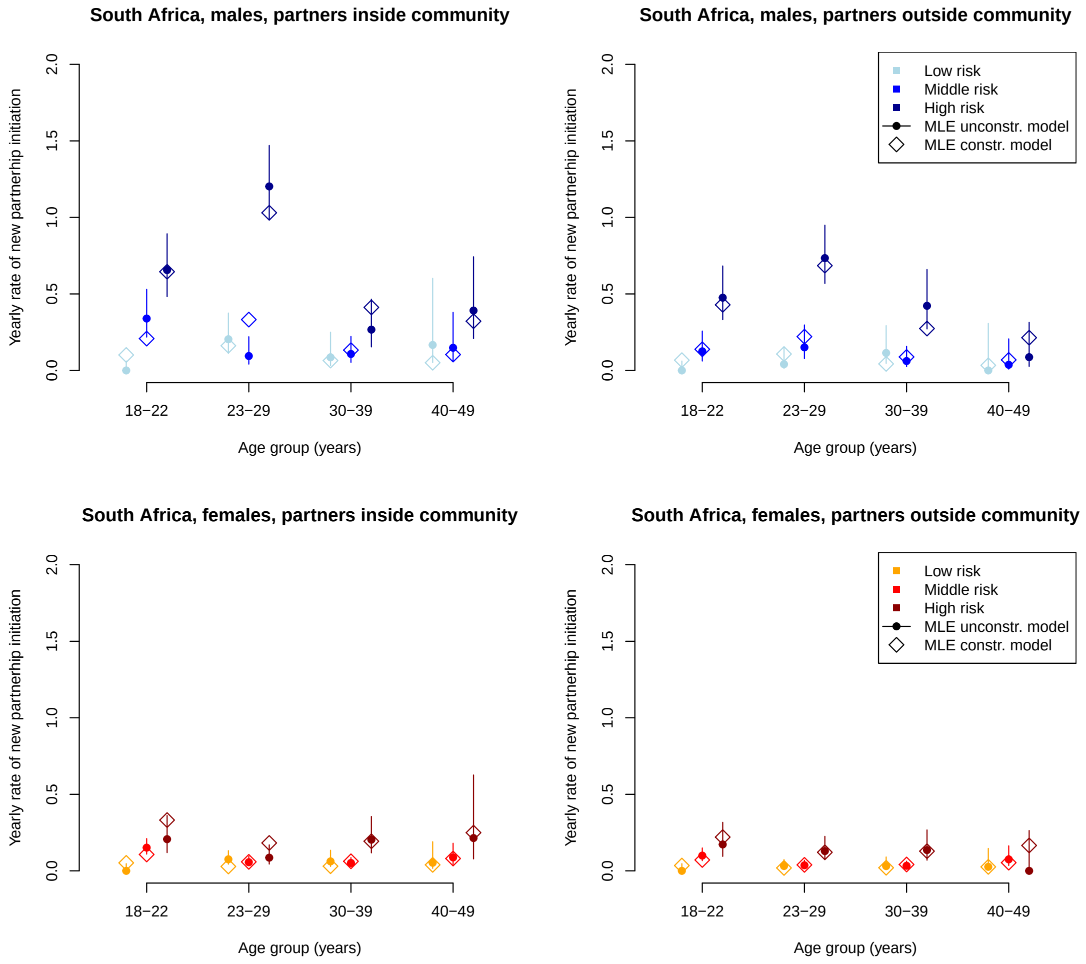

Fig H: Rate of partnership formation inside (left) and outside (right) the community, by age, activity class and sex for South Africa. Age groups not shown had too few observations to provide a reliable estimate. The diamonds correspond to the maximum likelihood estimates of the rates in our model, where the effects of age, activity class and location (inside/outside) are modelled using multiplying effects on the partnership formation rate (see text for details). The dots and confidence intervals correspond to the likelihood for the unconstrained model where rates of partnership formation inside / outside the community and in each sex/age/activity class are estimated completely independently.

3.5 The mechanics of partnership formation in PopART-IBM

In this section we describe the process of partnership formation as occurs in PopART-IBM at each step. The values and ranges for the partnership parameters is given in Table G.

Firstly, we compute the number of partnerships to be formed between each age and risk group, using equation 3, allowing for the multiplier on the rate of partnership formation $c_{multiplier}$ described in Section 3.4, and then the number inside and outside the patch using the parameter ξ defined in Section 3.3.4. Note that this may not be the actual number of partnerships formed at a given timestep. While PopART-IBM will try to form this number of partnerships, it may be that occasionally there are insufficient "available" potential partnerships, for example if everybody in a given subgroup has already got the maximum number of allowed partners as described in Section 3.2.2. In that case we reduce the number of partnerships to be formed to the number of "available" potential partnerships.

We then form the required number of partnerships. This is done by drawing randomly from the pool of 'available' partners, a list of the potential partners (stratified by patch, sex, age group, and activity level) available; note that if an individual has for example a maximum of 10 potential partners (i.e. they are in the 'high' activity level), but only has three current partners then they will appear in this list seven times since they could potentially have seven extra partners.

Partnerships are drawn systematically by group (so that for both men and women we go through the age/activity groups). Once the partnerships have been drawn for a specific group of men and a specific group of women, we go through each potential new partnership in turn to do the following checks:

- We validate that neither partner is dead.
- We check that the two individuals are not already in a sexual partnership with each other. If they are then we re-draw the partners in these specific groups of men and women, and check again that no two individuals are already in a partnership. This is repeated until successful or 10 attempts have been made; after 10 unsuccessful attempts the model moves on to the next age/activity group.

If the above conditions are satisfied for all the potential new partnerships in these specific groups of men and women, then the individuals form sexual partnerships in PopART-IBM. When a partnership is formed, the following steps occur:

- A partnership duration is drawn as described in Section 3.6, and a partnership breakup event is scheduled at that time.
- We update each individual's partnership characteristics: we increment by one the number of partners and number of lifetime partners of each individual, and we add the partner to the list of partners of each individual.
- If the partners are serodiscordant, we update the relevant serodiscordant partnership characteristics and list: we increment by one the number of seropositive partners of the seronegative partner, and we add the seropositive partner to the list of positive partners of the seronegative partner.
- If the partners reside in different patches, we increment by one the number of partners living outside the community of each individual.

- Each of the partners has one 'available partnership' removed from the list of available partnerships.

3.6 Modelling partnership duration

Once formed, a partnership is scheduled for dissolution at a future time (assuming neither partner dies in the interim), parameterised by PC0 data. We assume partnership durations to be exponentially distributed, with mean duration depending on the activity classes of the two partners (Figs I and J in Section 3.6.1 show that the assumption of an exponential distribution fits PC0 data well). We define mean duration by activity class μ_{low}, μ_{med} or μ_{high} (with $\mu_{high} < \mu_{med} < \mu_{low}$ as determined by analysis of PC0 data). The estimation of these parameters from PC0 is described in Section 3.6.1. When two individuals in PopART-IBM form a new partnership, the mean duration of their partnership is determined by the "higher" of the two sexual activity levels the partners belong to. For instance, a partnership between a high and a low activity level individual will have a duration exponentially distributed with mean μ_{high}.

The above parameters are used to model the duration of partnerships between partners in the same patch. We further allow partnerships between different patches to be shorter on average, with a multiplying factor ν derived from analysis of country-level data from PC0.

When a new partnership forms, and a duration is drawn randomly based on the sexual activity level of each partner and whether they are in the same patch, this duration is used to schedule the end of this partnership. However the partnerships will be dissolved prior to this expected end if one of the two individuals dies.

When a partnership is dissolved, all relevant characteristics and lists are updated. The list of sexual partners of each individual in the former partnership is updated. We decrease the number of current partners of each individual by one, and add an extra potential partnership from each ex-partner to the list of available partnerships. If the partnership was a serodiscordant one, then we update the list of serodiscordant partnerships to remove the dissolved partnership. We also update the list of HIV-positive partners of the negative ex-partner when the partnership was serodiscordant, checking if the negative ex-partner still has any other current HIV-positive partners; if they do not then we remove the ex-partner from the list of individuals who are in a serodiscordant partnership.

3.6.1 Estimating partnership duration from PC0

Estimating the distribution of partnership duration from PC0 data requires accounting for two main issues. First, there is right censoring, i.e. we observe partnerships which may be ongoing and we don't know when in the future they might end. Second, there is selection bias through which longer partnerships are more likely to be observed, precisely because they are longer. These issues have been described elsewhere, for instance in Burington et al. STI 2010 [9], but to our knowledge no method has been proposed to solve to estimate partnership duration from cross-sectional data which would fully account for

these issues. Here, we propose to first analytically derive the expected distribution of the observed duration of partnerships "so far", accounting for censoring and selection bias, given the true distribution of duration of partnerships. We will then show how this model for duration fits the PC0 data.

We assume that partnership distributions are exponentially distributed with mean duration $1/\lambda$. Consider n partnerships, where each partnership (indexed by i, $i \in \{1..n\}$) has a formation date S_i, and an end date E_i so that $E_i - S_i \sim Exp\left(\lambda\right)$. To closely mimic the process of data collection in PC0, we assume that individuals are asked at time t_2 about all their partnerships during the time interval $[t_1, t_2]$ (in PC0 this time interval $t_2 - t_1 = 1$ year). We call a partnership 'observed' if it would be reported , i.e. if the partnership started before t_2 and ended after t_1, and we define an indicator δ_i with value $\delta_i = 1$ if partnership i is observed at time t_2 ($S_i \leq t_2$ and $E_i \geq t_1$), and $\delta_i = 0$ otherwise. For the observed partnerships ($\delta_i = 1$), in the absence of information on whether partnerships are still active or not, we denote $D_i = t_2 - S_i$ the time between the start of the partnership and the time of interview.

We are interested in the distribution of D_i, given by the probability density function f.

$$
\begin{aligned}
f\left(t\right) dt &= P\left(D_i \in [t, t+dt], \delta_i = 1\right) \\
&= P\left(t_2 - S_i \in [t, t+dt], S_i \leq t_2, E_i \geq t_1\right) \\
&= P\left(S_i \in [t_2 - t - dt, t_2 - t], E_i \geq t_1\right) \\
&= P\left(E_i \geq t_1 | S_i \in [t_2 - t - dt, t_2 - t]\right) P\left(S_i \in [t_2 - t - dt, t_2 - t]\right) \\
&\propto P\left(E_i - S_i \geq t_1 - t_2 + t | S_i \in [t_2 - t - dt, t_2 - t]\right) dt
\end{aligned}
$$

So $f\left(t\right) \propto 1 - F_{Exp(\lambda)}\left(t_1 - t_2 + t\right) \propto e^{-\lambda t}$, where $F_{Exp(\lambda)}$ denotes the cumulative density function of an exponential with rate λ. Hence D_i is expected to be exponentially distributed with same rate as the true durations $E_i - S_i$.

An additional issue to account for is that at the time of interview, eligible individuals are aged 18-44. Hence very long durations of partnerships so far ($>$ 44 years) cannot be observed.

Therefore, we use a truncated exponential likelihood to describe the observations; we use maximum likelihood to estimate its parameter. We assume the duration depends on the activity class of the partner as well as whether the two partners are in the same community or not.

Figs I and J show the observed durations of partnerships by sexual activity level in PC0 by country, and the corresponding fitted durations. In general the model for duration reproduces the data well, and the assumption that the relative duration of partnerships outside versus inside the community is the same for all three activity levels makes little difference (as the black and red lines are generally close), apart from for low activity partnerships with the partner outside the community where there were few observations.

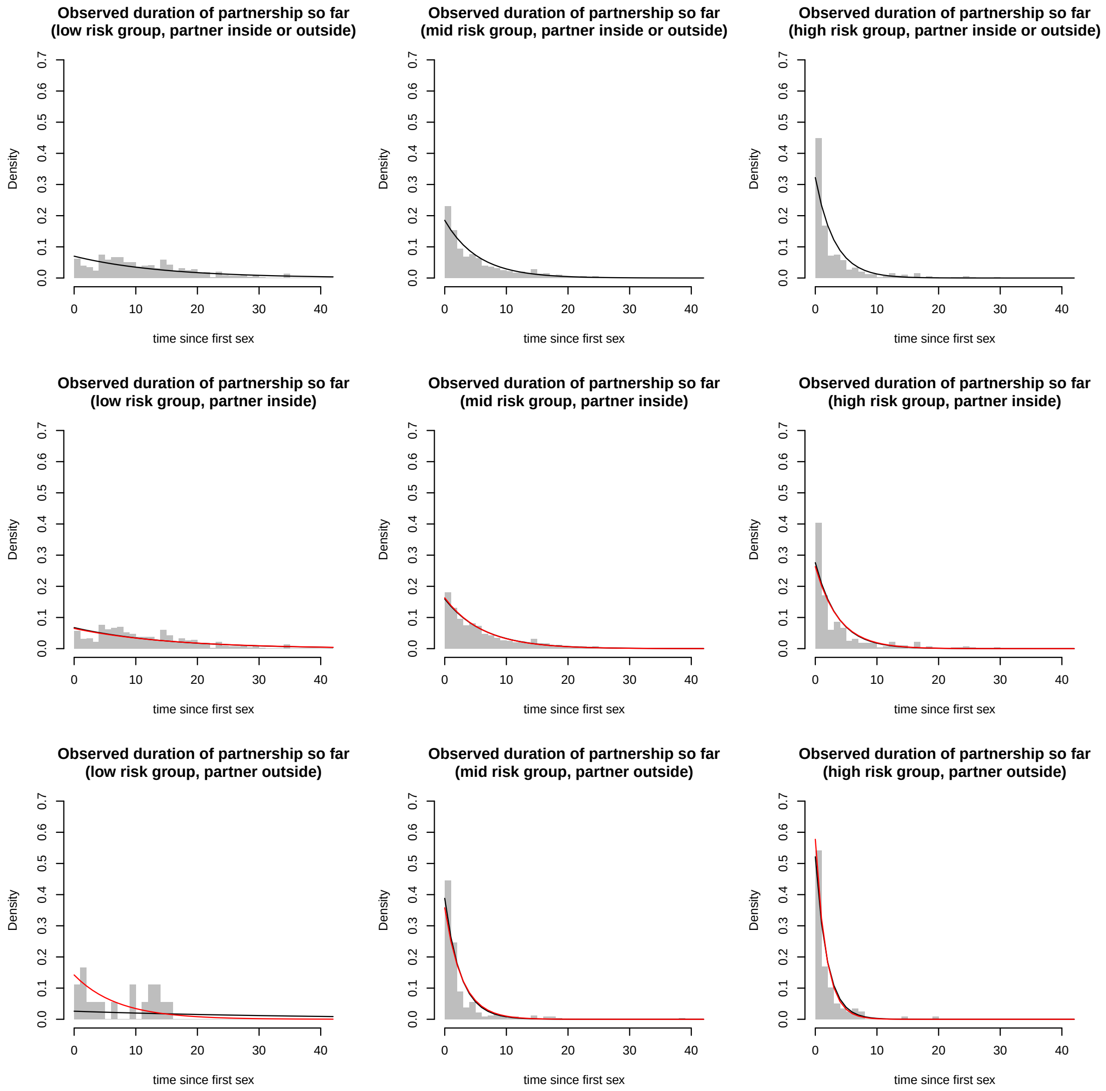

Fig I: Observed and fitted duration (in years) of partnerships so far for Zambia. The left, middle and right panels are for individuals in the low, medium and high sexual activity levels respectively. The top panels show all partnerships, the middle panels show partnerships with partners inside the community, and the bottom panels show partnerships with partners outside the community. The grey bars are the data; the black solid line the maximum likelihood estimate of the distribution for that activity class, and the red solid line (middle and bottom panels only) the maximum likelihood estimate of the distribution assuming that the relative duration of partnerships outside versus inside the community is the same for all three activity levels, which was used in the parameterisation of PopART-IBM.

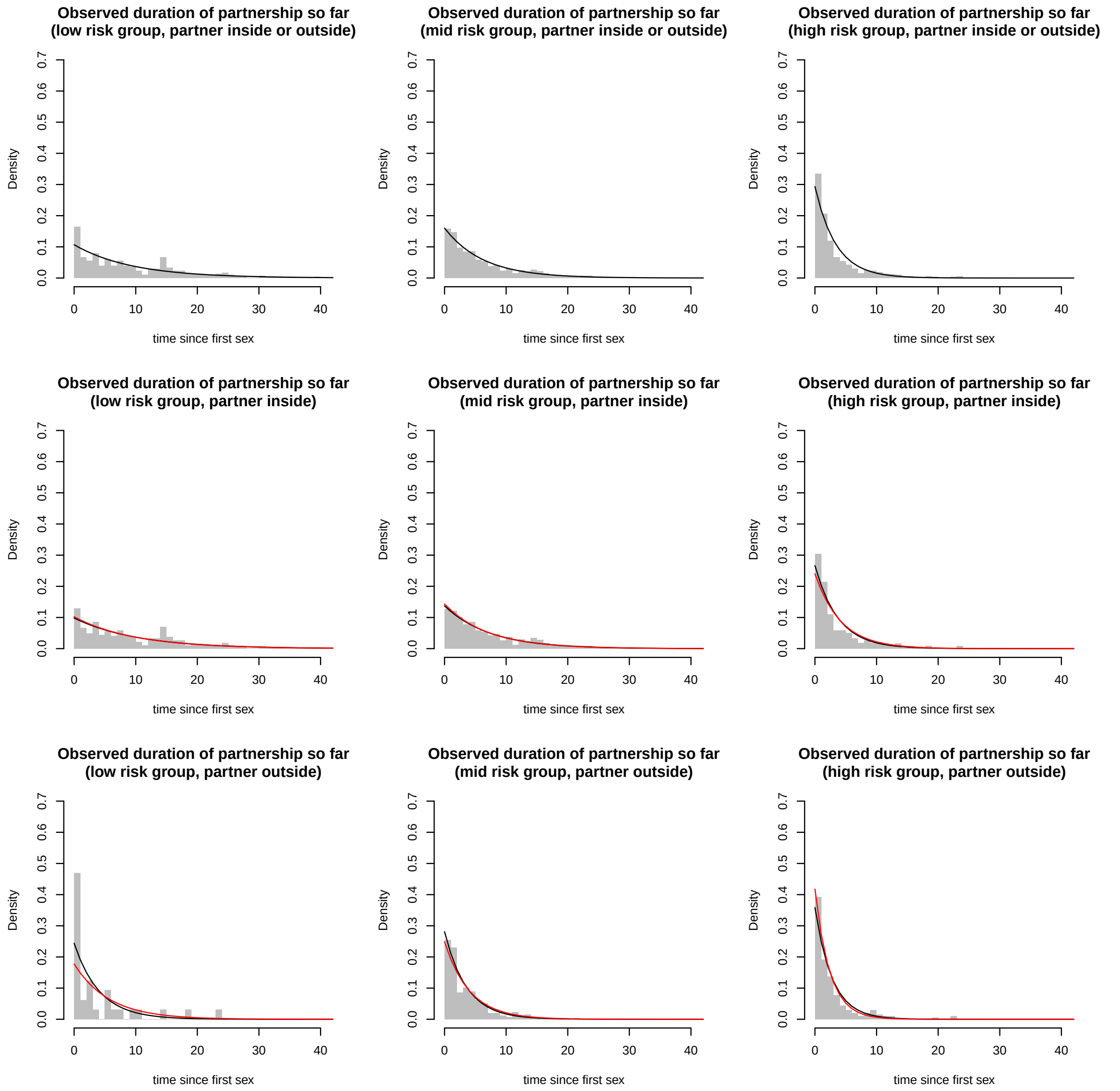

Fig J: Observed and fitted duration (in years) of partnerships so far for South Africa. The left, middle and right panels are for individuals in the low, medium and high sexual activity levels respectively. The top panels show all partnerships, the middle panels show partnerships with partners inside the community, and the bottom panels show partnerships with partners outside the community. The grey bars are the data; the black solid line the maximum likelihood estimate of the distribution for that activity class, and the red solid line (middle and bottom panels only) the maximum likelihood estimate of the distribution assuming that the relative duration of partnerships outside versus inside the community is the same for all three activity levels, which was used in the parameterisation of PopART-IBM.

4 HIV transmission, HIV testing and the ART cascade

In this section we describe the HIV components of PopART-IBM, which we take to consist of HIV transmission, HIV progression, HIV testing, initiation of antiretroviral therapy (ART) and other ART cascade events such as being lost to follow-up. AIDS-related death and emergency ART (individuals starting ART at low CD4 due to symptoms rather than after learning their serostatus following a routine HIV test) are included in this section as they are treated by the model as 'HIV progression' events.

Within PopART-IBM, individuals have several characteristics related to HIV and the ART cascade. These are:

- HIV status (uninfected, acute and early HIV infection (AEHI), chronic infection);
- CD4 compartment (CD4 $>$ 500, 350-500, 200-350 or $\leq$200 cells/mm^3);
- ART status if HIV-positive (not aware of status, aware of status but not yet on ART, early ART (started ART recently), on ART and virally suppressed, on ART but not virally suppressed, dropped out prior to starting ART, and dropped out when previously on ART);
- Set-point viral load (SPVL, the viral load of the individual during the period 6-24 months post infection).

These characteristics can get updated over time and can mutually interact - for example CD4 and set-point viral load influence infectivity - and this is described in this section. Male circumcision as a process is described later in Section 5.

4.1 Initializing the HIV epidemic

After the partnership network and age-distribution of the population have settled, at a given time in the simulation (`start_time_hiv`) HIV is introduced for the first time. This is a parameter sampled uniformly from the country-specific range given in Table M. Over the next 5 years, at the start of each year, a small percentage of the population is seeded to be HIV-positive, with a higher percentage amongst individuals in higher sexual activity levels. The percentage seeded is governed by a multiplicative parameter $F_{initial}$, sampled on a log scale for each run, to try to ensure that epidemics can be in different phases and have different values of R_0.

The seeding is done over several years to try to avoid the epidemic dying out due to stochastic fluctuations even though R_0 is greater than 1. While a larger seed could have been used at a single time point, significantly more people would be initially infected, and the model would likely need longer to forget the assumptions around who was infected (e.g. which age groups, sexual activity levels) in that case. A sensitivity analysis was carried out with a seed 3 times larger than the baseline value (results not shown) to ensure that the seed is not so small that it is constraining parameters (for example a very small initial seed may mean that R_0 needs to be larger, forcing the infectivity or partnership formation rates to be higher).

Table M shows the prior values/ranges for the parameters relating to HIV initialization in PopART-IBM.

4.2 HIV transmission

As described in Section 3 PopART-IBM explicitly models heterosexual partnerships between individuals. HIV transmission can only occur in the model between individuals who are in a serodiscordant sexual partnership. At each timestep any HIV-negative individual who currently has at least one HIV-positive partner is at risk of HIV infection, and the model keeps track of every such serodiscordant partnership. This list of serodiscordant partnerships is updated at each timestep as serodiscordant partnerships are created (either through an HIV-negative and HIV-positive person forming a new partnership, or when one partner in a previously concordant seronegative partnership gets infected through another partner) or destroyed (when the partnership breaks up, when either individual in the partnership dies, or when the seronegative partner in a serodiscordant partnership gets infected). Mother-to-child transmission is not modelled.

Within PopART-IBM we do not model explicitly condom use or frequency of sex acts with a given partner. Instead an HIV-negative partner in one or more serodiscordant partnership experiences a hazard of HIV transmission from each HIV-positive partner. This hazard is calibrated, and is affected by a number of behavioural and biological factors:

- The sex of the HIV-negative partner;
- The set-point viral load and stage of HIV infection of the HIV-positive partner (either AEHI, or CD4 stage if the infection is chronic);
- Whether the HIV-positive partner is on ART (and if so, whether they are in early ART, virally suppressed, or not suppressed);
- The circumcision status of the HIV-negative partner if male (including type of circumcision - i.e. medical or traditional). See Section 5 for more details and

**Parameter**	**Value**	**Notes**
Start of HIV epidemic (`start_time_hiv`)	1975 (Zambia) 1980 (South Africa)	Assumption
Number of years of HIV seeding after start of HIV epidemic	5	Assumption
Unscaled % of population seeded HIV+ each year by sexual activity level		
Low	0.002%	These are scaled by $F_{initial}$ below to
Medium	0.005%	get the actual % of the population
High	0.008%	seeded HIV+.
$F_{initial}$, factor multiplying seeded % at the start of the HIV epidemic	1-100	Assumption. Sampled on log scale

Table M: Parameters related to initializing HIV in PopART-IBM.

parameter values;

- The type of partnership (inside/between patches). In the the baseline HPTN 071 (PopART) population cohort survey (**PC0**) individuals who reported having a partner outside the community reported lower coital frequency and higher condom use with that partner than when both partners were in the community. See Section 4.2.1 for more details.

Each of these factors acts via a multiplicative cofactor on the baseline hazard, with all cofactors multiplying together (see Table N for ranges and values used. Note that circumcision-related parameters are given separately in Table R).

At each timestep, for each individual with at least one HIV-positive partner, the hazard of infection within the timestep is calculated from the baseline hazard, rescaling the hazard to be for a single timestep and adjusting for the above additional factors. Individuals with more than one HIV-positive partner are exposed to a total hazard that is the sum of the hazards from each partner. The total hazard is then the probability of infection in that timestep, and we draw from a Bernoulli distribution to determine if the individual will become infected in that timestep.

4.2.1 HIV transmission, condom use and frequency of sex acts

As described above, the HIV transmission hazard is lower when partners are not in the same patch, to reflect the lower frequency of unprotected sex reported in the Extended PC0 survey when the partner lives outside the community. Here we describe how we estimated this relative hazard based on the Extended PC0 survey data.

In the Extended PC0 survey, surveyed individuals were asked to report the frequency of sex acts with their last up to 3 partners in the last year, and to report whether they were using condoms all the time, sometimes or never with each of these partners. Following exploratory analyses which suggested that sexual behaviours might be different with partners inside and outside the community, we used these data to compare reported condom use and frequency of sex acts in partnerships with partners inside versus outside the community.

For those reporting more than 3 partners in the last year, we inferred the location of (inside/outside the community) and condom use with the extra (>3) partners in the last year in a deterministic manner based on the last 3 partners. We considered condom use as consistent if individuals responded "always" to the question on condom use, and to be inconsistent if they reported "sometimes" or "never" using condoms with a partner.

In order to compare the risk of HIV transmission in serodiscordant partnerships between communities and within a community, we looked at both condom use and coital frequency. We compared the proportion of partnerships in which condom use was reportedly used consistently among partners inside the community and partners outside the community, and found that reported condom use was higher with partners outside the community than inside, with an estimated relative hazard of transmission for between versus within community partnerships due to condom use of 0.75. We also compared the frequency of sex acts reported for partners outside versus inside the community, this

Parameter	Value	Source
β_{max}, annual transmission hazard in individuals with maximal SPVL	0.05-0.3 yr^{-1}	[10] estimated 0.313 yr^{-1} for individuals with SPVL 1 million copies/ml. Lower limit chosen to be more consistent with observed values in serodiscordant couples (e.g. [11]).
Hill function coefficients for modifying hazard by SPVL:		
β_k,	1.02	[10]
β_{50}	13,938 copies/ml	[10]
Relative infectivity by HIV stage (compared to chronic infection with CD4>500):		
- AEHI	5.3	[12].
- 350-500	1.0	Assumption.
- 200-350	1.0	Assumption.
- $\leq$200	2.34	[13].
Relative infectivity of male-to-female transmission (compared to female-to-male)	1.0-3.0	Lower limit no difference, upper limit is mean of low and high-income country estimates from [14].
Relative infectivity by ART stage (compared to no ART):		
$RR^{\text{early ART}}$, (early ART)	0.5	Assumption
RR^{VS}, (on ART and VS)	0.0	No infections in [15] when VS. In pessimistic ART sensitivity analysis, a value of 0.07 is used.
RR^{VU}, (on ART and VU)	0.7	Assumption to get overall effectiveness of 0.93 in [15] if 90% on ART are VS. In pessimistic ART sensitivity analysis, a value of 0.5 is used.
Relative HIV transmission risk for partnerships between patches, compared to partnerships within same patch	0.452	Pooled analysis from all PC0 analysis (i.e. Zambia and South Africa together). Partnerships between patches are assumed to have different unprotected coital frequency (see 4.2.1).

Table N: HIV transmission-related parameters used in PopART-IBM. ART=antiretroviral therapy. VS=virally suppressed. VU=virally unsuppressed. SPVL=set-point viral load. PC0=baseline HPTN 071 (PopART) population cohort survey. Note that circumcision-related parameters are in Table R

time only considering the last up to three partners, as it would be difficult to accurately infer a continuous variable from only three observations. We found that, unsurprisingly, reported frequency of sex act was lower with partners outside the community than inside. We therefore assumed that the risk of HIV transmission would be further decreased in serodiscordant partnerships between compared to within communities, with an estimated relative hazard of transmission for between versus within community partnership due to frequency of sex acts of 0.602. Overall, we therefore assumed that the risk of HIV transmission within a serodiscordant partnership with partners from different communities was 0.452 times that in a serodiscordant partnership with partners from the same community.

4.3 Immediately post-infection

Once an individual becomes HIV infected, the following events happen to them in the model:

- They enter the acute and early phase of HIV infection (AEHI);
- They are scheduled to end AEHI 1-3 months after the current time. The time of the end of AEHI is drawn uniformly between that range, separately for each individual;
- They are assigned a set-point viral load (**SPVL**) (see Section 4.5 for more details);
- Based on their SPVL (see Section 4.5 for details), they are assigned a CD4 compartment (>500, 350-500, 200-350 or ≤ 200 cells/mm^3) which they will enter once they leave AEHI reach chronic infection.
- Their seronegative partners are added to the list of serodiscordant partnerships (as the newly infected individual is now in serodiscordant partnerships with those people), while the list of serodiscordant partnerships is updated to remove the newly infected individual's partnerships that have become seroconcordant positive.
- We increment the counter of the total number of HIV infections that have ever occurred in the population. This counter is outputted over time to count total population incidence.

Seeded infections (those individuals who are made HIV-positive at the start of the epidemic) are assumed to not enter AEHI but to start in a random CD4 compartment with the same probability distribution as an individual leaving AEHI, with duration chosen as for that given CD4 stage. Otherwise they undergo the same steps as other individuals who become infected.

4.4 HIV progression when not on ART

Here we describe how HIV progression occurs for an individual who is not currently on ART (including both those who are ART-naïve, and those who were previously on ART but have stopped treatment).

Once an individual leaves AEHI, they enter a CD4 compartment (>500, 350-500, 200-350 or ≤ 200 cells/mm^3) drawn at random as described in Section 4.3 above (see Fig 2 in the main text for an illustration of HIV progression). They will then progress sequentially to lower CD4 categories until they reach CD4 ≤ 200 cells/mm^3. After that compartment the next stage, if the individual does not start ART, is AIDS-related death; individuals are assumed to only die from AIDS-related illness in the model once their CD4 is below 200 cells/mm^3.

The time each individual is scheduled to spend in a given CD4 compartment is drawn from an exponential distribution with mean time based on the analysis in Cori et al. [16],

which analysed the rate of CD4 progression of the ATHENA cohort of HIV-positive individuals in the Netherlands by set-point viral load. Based on that analysis, within PopART-IBM individuals with higher set-point viral load progress more quickly on average, as described in Section 4.5.

'HIV testing and the ART cascade' is treated as a separate process from HIV progression within PopART-IBM, so that initiating ART may be viewed as being a competing hazard. In practice this means that individuals are still scheduled to progress to the next CD4 compartment as described below, but if they are scheduled to start ART before the next HIV progression event then ART initiation takes precedence and modifies the future HIV progression as described in Section 4.7.

4.5 Set-point viral load

Within the model, set-point viral load (SPVL, the viral load of the individual during the period 6-24 months post infection) modifies infectivity. SPVL also affects the initial CD4 compartment of a new infectee and the rate of CD4 progression, as previously mentioned in sections 4.3 and 4.4 respectively.

Each individual, upon seroconversion (including those who are seeded HIV-positive at the beginning of the epidemic), is assigned a set-point viral load v (measured in viral copies/ml), with the $\log_{10}$ of their set-point viral load drawn from a normal distribution: $\log_{10} v \sim N(4.74, 0.61^2)$, based on data from a cohort in Zambia [17].

As described in Section 4.4, after a short period of AEHI, individuals enter chronic infection. They enter an initial CD4 compartment ($>$ 500, 350-500, 200-350 or $\leq$ 200 cells/mm^3). The compartment is drawn randomly for each individual, and the probability of being in a given CD4 compartment is dependent on their SPVL. Following the analysis in Cori et al.[16] of data from the ATHENA cohort in the Netherlands we use 4 categories for $\log_{10}$ SPVL ($\leq$ 4, 4-4.5, 4.5-0, $>$ 5.0). For each individual we work out the SPVL category given their category, and then using the corresponding probability distribution from [16] (see Table O for values) we draw their initial CD4 compartment.

4.5.1 Set-point viral load and HIV transmission

Based on [10] we assume that the average annual hazard of transmitting HIV $\beta(v)$ of an individual with set-point viral load v is described by the formula:

$$\beta(v) = \frac{\beta_{max}\ v^{\beta_k}}{(v^{\beta_k} + \beta_{50}^{\beta_k})}$$

where the parameters $\beta_k = 1.02$ and $\beta_{50} = 13,938$ copies per ml, and β_{max} is the average annual hazard of (uncircumcised) man getting HIV from an HIV-positive partner who has maximal SPVL, which is a parameter varied in the calibration uniformly from 0.2 to 0.6 yr^{-1} (see also Table N). By way of comparison, in [10] a value of $\beta_{max} = 0.317$ yr^{-1} was found for a Zambia-based cohort.

4.5.2 Set-point viral load and HIV progression in the absence of ART

Set-point viral load v modifies the length of time a person not on ART will spend in a given CD4 compartment as follows. We assume that individuals with $\log_{10} v \leq 4$ would spend a time in a given CD4 compartment c drawn uniformly from the range given by [16]. For individuals with higher SPVL, we draw the baseline time $t^c_{baseline}$ from the same range, and we then adjust it by a factor depending on their SPVL. The time they would spend in the CD4 compartment c is

$$t^c_{baseline}/\mathrm{S}_c^{(\log_{10} v - 4.0)}, \qquad (\log_{10} v > 4) \tag{4}$$

where S_c is a factor, depending on the current CD4 compartment, which comes from analysis of ATHENA cohort data [16]. The parameter S_c determines how much time an individual with higher SPVL (i.e. $\log_{10}$SPVL> 4) will spend in a given compartment.

4.6 HIV testing

HIV testing is divided into different testing routes, representing multiple potential delivery channels. For example in the context of the HPTN 071 (PopART) trial there are two routes: 'background' HIV testing, and 'PopART' HIV testing (also called 'CHiPs testing'). The former represents the testing that takes place outside the trial: testing through clinics, as part of antenatal care, etc. The latter is the household-based testing by community health workers, known as CHiPs (community HIV care providers), in the trial. These two different testing routes are treated as separate processes in PopART-IBM, and the testing rate and linkage to care for each route are parameterised separately, reflecting that these routes may have different outcomes: the home-based CHiPs testing will, by design, reach individuals who may have been less inclined to actively seek out healthcare; in addition CHiPs teams actively re-visit known HIV-positives to encourage linkage to care, which may facilitate individuals initiating ART. Note that within intervention communities, additional activities to promote HIV prevention and to reduce stigma around HIV testing have been carried out; while these events may also increase 'background' testing (as individuals may choose to go to a clinic for testing due to increased awareness), the rate of background testing is assumed in the model to be unchanged in intervention communities compared to the simulated control group. This is a conservative assumption for trial impact, as it may lead to underestimates of true trial impact.

We first describe the 'background' HIV testing in Section 4.6.1. The 'PopART' HIV testing, carried out by CHiPs teams, is described in detail in Section 4.6.2.

4.6.1 Background HIV testing scheduling

As described above, 'background' HIV testing means testing through standard channels (in other words everything except additional testing through the trial via CHiPs), for example through antenatal testing, or individuals going to clinics to get tested. We divide time up into fixed periods (e.g. 2000-2006, 2007, 2008...), and during each fixed period

**Parameter**	**Value**	**Source**
Distribution of initial CD4 category after HIV infection, by $\log_{10}SPVL$ category:		
$\log_{10}$SPVL$\leq$ 4.0:		
- CD4>500:	0.864	Analysis in [16]
- CD4 350-500:	0.113	
- CD4 200-350:	0.023	
- CD4$\leq$200:	0.000	
$\log_{10}$SPVL 4.0-4.5:		
- CD4>500:	0.780	
- CD4 350-500:	0.190	
- CD4 200-350:	0.030	
- CD4$\leq$200:	0.000	
$\log_{10}$SPVL 4.5-4.0:		
- CD4>500:	0.740	
- CD4 350-500:	0.210	
- CD4 200-350:	0.050	
- CD4$\leq$200:	0.000	
$\log_{10}$SPVL >5.0:		
- CD4>500:	0.710	
- CD4 350-500:	0.250	
- CD4 200-350:	0.040	
- CD4$\leq$200:	0.000	
**HIV progression when not on ART:**		
t_{acute}, Duration of AEHI	0.08-0.25 yrs	[12]
For individuals with $log_{10}SPVL < 4.0$:		
- $t^{>500}_{baseline}$, time in CD4 > 500 category	4.56-6.37 yrs	[16]
- $t^{350-500}_{baseline}$, time in CD4 500-350 category	2.98-4.53 yrs	[16]
- $t^{200-350}_{baseline}$, time in CD4 350-200 category	5.04-13.69 yrs	[16]
- $t^{\leq 200}_{baseline}$, time in CD4 $\leq$ 200 category	1.8-2.8 yrs	[13]
S_c:*Factor by which time in CD4 category c is decreased, per 10-fold increase in SPVL*		Analysis of ATHENA cohort data [16]
CD4 > 500	2.17	
CD4 500-350	1.88	
CD4 350-200	1.96	
CD4 $\leq$ 200	1.63	
$f^{VU progression}$, multiplier for increased duration in each CD4 stage when on ART but VU	1.0-2.0	[13]
$\log_{10}$SPVL of newly HIV-infected person (in $\log_{10}$ copies/ml)	$\sim$N(4.74, 0.61^2)	Data from Zambian cohort in [17]

Table O: HIV progression-related parameters used in PopART-IBM. ART=antiretroviral therapy. VU=virally unsuppressed. SPVL=set-point viral load.

we assume that, apart from those PLHIV who already know their status, everyone has a set probability of having an HIV test. This probability differs by sex, being higher for women, and varies by period. At the start of the period we go through the whole adult population and, for those who are not already HIV+ and aware of their status, we draw randomly to determine if they will have an HIV test during the period. If they are going to have a test, we draw a test date uniformly from the fixed period, converting to a discrete timestep, and schedule a test for the individual in the model at that future time.

The first fixed period is 2000-2006, when HIV testing rates were low. After 2006 yearly intervals are used for the fixed periods, with a constant probability of being tested each year. Within the model that probability is the product of a baseline probability for women that varies over the two periods (2000-6 and 2007 onwards), and a fixed multiplier for men compared to women. There are thus three parameters, and all are allowed to vary freely, being fitted at the calibration stage. Main text Fig 5 shows how the calibrated runs compare to the CHiPs data by age and sex on the proportion of people living with HIV (PLHIV) who know their status just before the first visit, and the proportion of those PLHIV aware of their status who are on ART, as well as the proportion of PLHIV who are virally suppressed. Generally these outcomes are comparable to the observed data.

4.6.2 CHiPs HIV testing scheduling

Within the HPTN 071 (PopART) trial CHiPs teams aim to visit every household in a given 'round' (a round is approximately one year, although round 1 lasted longer). In the current paper we show results for a scenario without the trial intervention. We therefore only include a description here of how the intervention is implemented in the model for completeness.

When visiting a household, CHiPs teams record information on all individuals seen. This CHiPs data includes the number of people who are successfully visited by CHiPs, the outcome of that visit (for example they may accept an HIV test and receive the result, self-report that they are HIV-positive or report having had an HIV-negative test in the past 3 months), their age and sex, as well as the date of the visit.

When modelling the intervention, Anonymised CHiPs data is used, on a community-by-community basis, to parameterize the proportion of individuals, by age (using one-year age groups) and sex, successfully visited at each timestep. Within the model this is subsequently converted into a number of individuals who are to be visited in that timestep. Given that the community population size in the model at the time of the trial may not exactly match that in reality (since the model is begun in 1900 and the population grows over time), this ensures that the proportion of the population reached by CHiPs teams (i.e. coverage) is the same in the model as occurs in the trial. The proportion visited varies from timestep to timestep so that the process of CHiPs visits over time in the model exactly mimics what happened in reality over these rounds.

Within PopART-IBM, this data is used as follows: at the beginning of the CHiPs round, the adult population in the model is divided up by age and sex. For a given

age a and sex g, the total number of people in each group $n_{g,a}$ scheduled to be visited throughout that round is calculated based on the CHiPs data. The model draws $n_{g,a}$ individuals randomly from all those of age a and sex g, and stores them into a list. This list is then randomly shuffled (to ensure that there is no ordering within each list) and split up into subsets where each subset contains the correct number of people for that age and sex group to be visited at a given timestep. Future CHiPs visits are then scheduled in the model for these individuals. Because these visits are scheduled in advance, some individuals, who are scheduled to be visited by CHiPs at this point in the model, may die prior to the CHiPs visit. However this is assumed to be negligible for simplicity. This assumption was tested for community 2 for the baseline scenario. In this case around 100-150 individuals who were scheduled for a CHiPs visit died prior to the visit out of an adult population of 45,000, around 0.5% of all individuals to be visited, suggesting that this assumption is indeed valid.

4.6.3 HIV testing from the individual's point of view

While HIV test scheduling is done within the model at the population level, the test itself and the subsequent outcomes are determined for each individual separately. Upon having an HIV test, whether this test is due to CHiPs or background testing, an individual may receive a positive or negative test result. The result of an HIV-negative person is taken to be always negative (100% specific). However an HIV-positive person may test negative due to a 'window period' in AEHI, when the necessary antibodies/antigens may not yet be present for detection [18]. The window period has reduced over time as later generation HIV tests have been adopted within country, and is taken to last 60 days until 2006, and 30 days thereafter, with the latter corresponding to the window period of more recent antigen/antibody tests [19].

Individuals who receive a negative HIV test can have new future HIV tests scheduled either by CHiPs testing (if CHiPs operate in the community) or by background testing. Men testing HIV-negative (either through CHiPs or background testing) may then undergo voluntary male medical circumcision, as described in Section 5.

In Section 4.7 below we look at what happens to individuals who receive a positive HIV test.

4.7 The care cascade

We divide the care continuum for HIV-positive individuals into discrete states as follows (note that we are including states, such as HIV+ unaware, in the care continuum; in reality such states are normally referred to as outside care):

- *HIV+ unaware*: individuals who are HIV-positive but have never received a positive HIV test result. This may include people who have received a false negative HIV result (and have never received a true positive HIV result).
- *HIV+ aware*: individuals who are HIV-positive, have received a positive HIV test result, and have scheduled their CD4 test/are awaiting their CD4 test results/are

eligible for ART and are scheduled to start ART. Note that in reality even within the Arm A HPTN 071 (PopART) community clinics, where ART is started regardless of CD4 count, a CD4 test is normally required prior to starting ART, and the model therefore mimics this waiting period.

- *In care, not on ART*: have had a CD4 test and are currently not eligible to start ART, as they lie outside treatment guidelines at the time. They have not decided to drop out, meaning that they have another CD4 test result scheduled in the future to check eligibility once more.

- *Early ART*: recently started ART. Once an individual starts ART they have a short period where their viral load is high, even if they will eventually become virally suppressed. During this time they may also be at higher risk of mortality if their CD4 count is low [20]. In the model, they will have partly reduced infectivity and we assume they will not undergo CD4 progression during this period for simplicity as the period is relatively short.

- *On ART virally suppressed*: once an individual has been on ART for some time, they will generally become virally suppressed, meaning that their viral load is undetectable. In the model, their infectivity is substantially reduced compared to someone who has never been on ART who has the same CD4 and SPVL, and they do not undergo CD4 progression.

- *On ART but virally unsuppressed*: individuals who have been on ART for some time but whose viral load is detectable (which in reality may correspond to, for example, taking pills sub-optimally). In the model, they will have partly reduced infectivity and slower CD4 progression than an individual who has never been on ART (but who has the same CD4 and SPVL).

- *Dropped out of care*: includes both individuals who were on ART but have since stopped taking pills, and those who received an HIV-positive test but dropped out before ever starting ART. In the model, they are assumed to have the same infectivity and HIV progression as an individual who has never been on ART (but who has the same CD4 and SPVL). These individuals have no scheduled cascade events at this time.

A schematic overview showing the allowed transitions between states for this process in the model, including the transition from being unaware of HIV status, is shown in Fig K. We now describe the transitions in more detail. Table P gives the corresponding parameter ranges for this process.

ART eligibility Eligibility for ART in PopART-IBM is determined as follows: over time the eligibility criteria in real life for initiating ART have changed, and within PopART-IBM the CD4 categories used reflect the different CD4 thresholds that have been in place in these different periods. In the model the rules for initiating ART mirror

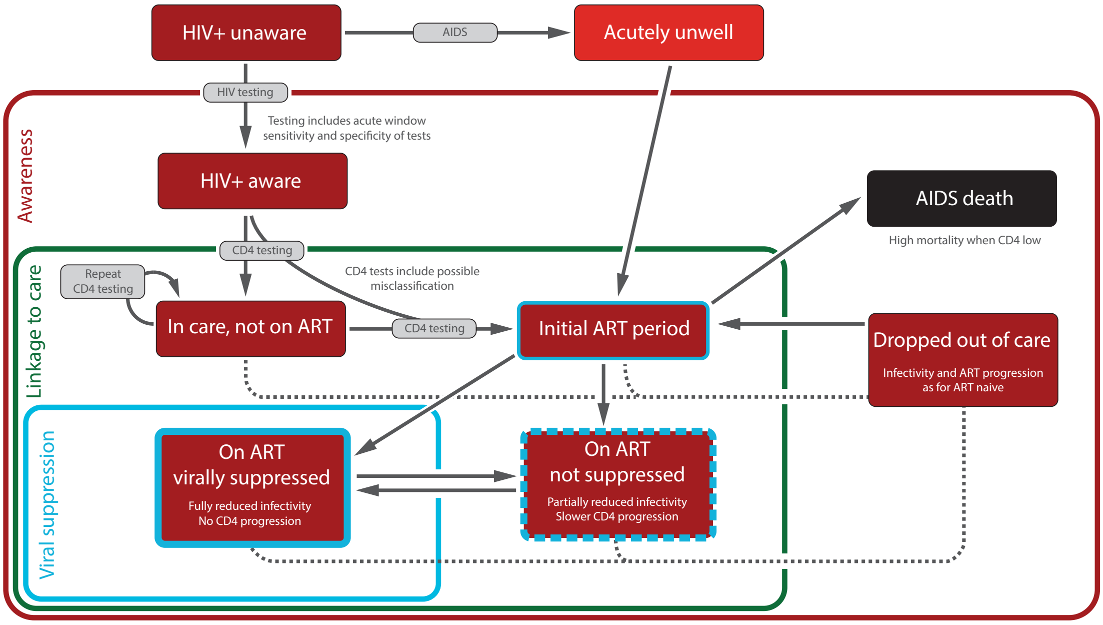

Fig K: Schematic of the HIV care cascade in the model.

the changes within the community. These changes follow changes in national guidelines, apart from in Arm A communities where since the beginning of the trial ART has been available regardless of CD4 count. Table P shows the dates at which guidelines changed by country. In Arm A communities in PopART-IBM it is assumed that ART has been available regardless of CD4 count since the beginning of the trial, which is defined as the start of CHiPs visits in the community.

Transitions after becoming HIV aware After receiving a positive HIV test result, an individual becomes HIV+ aware. At this point they will be scheduled to move next into one of three states in the model: dropped out of care, in care but not on ART (when they are not currently eligible for ART in the patch in which they reside), and early ART (if eligible for ART in the patch in which they reside). Note that these transitions in PopART-IBM may consist of several steps in real life, such as registering for care and having a CD4 test.

Upon receiving a positive HIV test result we first decide whether that individual will drop out of care (i.e. will neither initiate ART nor remain in care until their next CD4 test to determine ART eligibility). This corresponds to an individual in the real world who either drops out before collecting their HIV test results, or collects their HIV test results but leaves prior to starting ART (if eligible) or before their CD4 retest is due (if not eligible for ART at the first CD4 test). In order to count the relative number of HIV and CD4 tests we therefore include probabilities for both events. For PopART testing the probability of dropping out before collecting HIV test results is taken to be zero since

the CHiPs data used is that from ‘successful’ visits: a successful visit means that the individual has an HIV test and receives the results. Similarly the analysis of time to start ART following a positive HIV test (Section 4.7.1) incorporates the possibility that an individual does not start ART from the CHiPs visit: if the sampled time to initiate ART is after the CHiPs visit in the following round, then during the following round the time to start ART is redrawn, effectively meaning that the individual "dropped out" during the earlier round, only to be able to relink in the following round.

For background testing the probabilities $p^{background}_{collectHIVresults}(c)$ (depending on the current CD4 category c of the individual) and $p^{background}_{collectCD4results}$ represent the probabilities of collecting the HIV test result, and of initiating ART/remaining in care if they have received a positive HIV test result (see Table P for the ranges/values) of these parameters.

For individuals who remain in care rather than dropping out, the model determines whether they are eligible for ART given their current CD4 and the guidelines in the patch they reside as described above. If they are not eligible for ART, then they are scheduled to have a new CD4 test at a time in the future to check eligibility once more. For each CD4 retest the time to retesting is uniformly sampled from a range which can depend on whether the original HIV test was a CHiPs test ($t^{CD4\ retest}_{PopART}$) or background test ($t^{CD4\ retest}_{background}$).

Those eligible for ART have initiation of ART scheduled when they become aware that they are HIV-positive (if they do not drop out, and if they are eligible to start ART). The distribution of the time to start ART depends on whether the HIV test was a CHiPs test or a background test (since additional effort is made by CHiPs to link newly diagnosed individuals to care, it is to be expected that the time to start ART may differ in this way). For CHiPs testing we take a bi-exponential (so that individuals are divided up into ‘fast’ and ‘slow’ initiators), based on data from CHiPs corresponding to the time taken to start ART. Section 4.7.1 describes how this time is derived using CHiPs data, and the corresponding parameters are in Table Q. For background testing, given the lack of information about how long it takes to start ART when eligible from background testing, we assume the time to start ART is exponentially distributed. We conservatively take the mean time from analysis of CHiPs data for the given country, so that individuals eligible and starting ART who are not in PopART Arm A/B communities will start (on average) as quickly as those in Arm A/B communities.

Emergency ART, where an individual seeks treatment due to a low CD4 count and associated symptoms, is described in Section 4.8, and is treated as an independent process within PopART-IBM.

In care, not on ART Within PopART-IBM, this corresponds to the period between the positive HIV test and starting ART for individuals who were initially ineligible for ART (due to being outside ART initiation guidelines). Individuals in an Arm A community are ineligible for ART if their CD4 count is above guidelines only until the start of the trial. In Arm B, patch 1, and for the simulated counterfactual (shown in this paper), an individual whose CD4 category is above the guidelines is ineligible for ART (see Table P for how ART guidelines changed in the model). They would then have their

CD4 retested periodically (at an approximately yearly schedule) until either they were eligible or they dropped out of care. In background testing the probability of remaining in care after another ineligible CD4 test result is assumed to be $p^{cd4}_{background}$, while in PopART arm B communities it is assumed to be 1 due to the more active linkage to care facilitated by CHiPs.

Note that when guidelines change (even in Arm A trial communities), individuals who were in care but not on ART do not automatically begin ART; they can begin only at their next scheduled CD4 test (or in the case of communities with CHiPs, they may begin shortly after a CHiPs visit, with the delay given by the time between receiving a positive HIV test and initiating ART for someone who is eligible - thus reflecting the fact that CHiPs teams do not provide ART themselves, and the PLHIV must still visit a clinic).

Initiating ART (not emergency ART) Individuals who are eligible to start ART, and are in care, are scheduled to start ART shortly afterwards. For background testing this time is exponentially distributed with mean $t^{ART}_{background}$ years, while for PopART it is drawn from a biphasic exponential that depends on the CHiPs round.

Upon starting ART, an individual firstly enters a state of 'early ART' during which they are not yet virally suppressed, lasting a short duration $t^{\text{early ART}}$. During this time they have a reduced infectivity $RR^{\text{early ART}}$. Individuals may subsequently end up in one of three states: they may remain adherent and on ART and become virally suppressed; they may remain on ART but with poor adherence and be virally unsuppressed; or they may die, due to complications from immune reconstitution inflammatory syndrome, which is assumed in the model to occur only in individuals with CD4≤ 200 (with probability $p^{\text{early ART}}_{die}$). We assume that, of those who do not die during early ART, a proportion $p^{\text{become VS}}$ of individuals become virally suppressed afterwards. This parameter governs outputs on viral suppression in the model.

Becoming virally suppressed An individual who is virally suppressed is assumed to keep their CD4 at the time of initiating ART without progressing any further or increasing their CD4 through the benefits of ART, provided they remain on ART and virally suppressed. They are also assumed to have very reduced transmissibility of HIV, by a factor RR^{VS}. However over time they may become virally unsuppressed, or drop out of ART.

Data from a Zambian cohort in Lusaka [20] shows that over about 8 years roughly 30% of those successfully initiated on ART have dropped out, while around 13% have poor adherence. In PopART-IBM, on becoming virally suppressed an individual has a next event drawn. The possible next events are: remain virally suppressed for life with probability $p^{remainVS}$ (for women) or $p^{remainVS} * p^{staysVSmale}$ (for men); become virally unsuppressed in the future with probability $p^{VSbecomeVU}$; or drop out from care with probability $p^{VSdropout}$. If either of the latter, a time to that event happening $t^{\text{End VS}}$ is drawn, with a prior range roughly corresponding to the timescale observed in [20].

Becoming virally unsuppressed An individual who is virally unsuppressed is assumed to undergo CD4 progression but at a rate reduced by a factor $f^{VUprogression}$ than someone not on ART, an assumption also previously made in [13]. They also have reduced infectivity compared to someone not on ART by a factor RR^{VU}. Individuals who are virally unsuppressed are assumed to eventually drop out, ceasing ART, as they are already poor adherers, at a time $t^{\text{End VU}}$.

Dropped out of care to early ART Upon dropping out, infectivity is no longer reduced. For individuals who were previously virally suppressed, they had no HIV progression event scheduled (i.e. their CD4 remained unchanged while virally suppressed); for these individuals CD4 progression restarts at the same rate as when not on ART

Individuals who have dropped out can re-enter care in one of two ways. If PopART is taking place in their patch, they may receive a CHiPs visit (or if they dropped out in a previous round, they may successfully link in the current round). Outside of PopART (i.e. in patch 1, or in patch 0 before the start of the trial, or in any patch for the simulated counterfactual scenario without intervention) they may start emergency ART when their CD4 is low (red dashed lines in Fig K), as described in 4.8.

4.7.1 Time between an HIV-positive test result by CHiPs and initiation of ART

Uptake of ART after a CHiPs visit is modelled using a bi-exponential distribution: we assume that among PLHIV not on ART identified by CHiPs, a proportion q will initiate ART more quickly (with exponential distribution at rate ν_{fast}) and a proportion $1-q$ will initiate ART more slowly (with exponential distribution at rate ν_{slow}). Hence, the proportion who initiate ART by time t after a CHiPs visit is given by $q\left(1-e^{-\nu_{fast}t}\right)+(1-q)\left(1-e^{-\nu_{slow}t}\right)$.

Note that individuals who have not yet initiated ART (having had an HIV-positive test in an earlier CHiPs round) when they receive the CHiPs visit of the subsequent round get ‘reset’ - that is, their time of ART initiation is assumed to be driven by the latest CHiPs visit, and so the time to start ART is redrawn. In the individual based model, this means that occasionally, an individual can get rescheduled his/her start of ART to a later date following a new round of CHiPs visit. This assumption may seem odd but is closest to the way the data was analysed. It also means that there is an implicit probability of not starting ART during a given round.

We fitted this bi-exponential model, using a maximum likelihood approach, to the CHiPs intervention data from round 1, with analyses stratified by country, and year quarter, in order to get round 1 estimates. Note that in Zambia the intervention started earlier, so that there are 6 quarters in the first round (which lasted 1.5 years) but only 5 quarters in South Africa (lasting 1.25 years). For rounds 2 and 3 there was less variation over time within the round, and the analysis was stratified by country but not by quarter.

The bi-exponential model presented above was favoured over a saturated exponential model (similar to the bi-exponential model but with $\nu_{slow}=0$, i.e. a proportion $(1-q)$

never initiate ART), as the bi-exponential model produced a much better fit to the data.

4.8 Emergency ART

Hallett et al. have hypothesized that individuals with low CD4 may start ART outside of the standard HIV testing care cascade, since data suggests that a large percentage of patients initiate treatment at late stages of infection [21]. In PopART-IBM there is a process which we term 'emergency ART' in the model, whereby individuals who have CD4 below 200 cells/mm^3 can quickly start ART without having had a prior HIV test once they have clinical symptoms. An individual who reaches the CD4$\leq$200 cells/mm^3 compartment in the absence of ART will have had a time to AIDS-related death assigned to them, as described in Section 4.4. They will then have a second time drawn from the same distribution, which functions as a competing hazard for starting emergency ART. At the population level an individual therefore has a 0.5 probability of starting emergency ART rather than dying, but individuals who have a longer time to AIDS-related death will be more likely to start emergency ART before dying.

Initial runs suggested that this has a small effect over the course of the trial compared to runs where it is switched off (results not shown), while Fig R shows the distribution of transitions that the population of PLHIV undergoes in the cascade annually. In particular, while emergency ART is important in early years in getting individuals onto ART (especially when ART initially becomes available, when there is a pool of individuals with low CD4 who are eligible to start ART), once HIV testing is increased by 'PopART' CHiPs household testing it becomes much less important.

Parameter	Value	Source
Time when background HIV testing begins	2000	Assumption
Time when ART first available	2004	Assumption
Time when ART guidelines changed to CD4<350	2011	
Time when ART guidelines changed to CD4<500	2014.5 (Zambia) 2015 (S Africa)	
Time when ART guidelines changed to immediate treatment	2016.33 (Zambia) 2017.0 (S Africa)	As reported by trial community clinics .
Probability of a woman having an HIV test before 2006	[0-1]	Allowed to vary freely.
Annual probability of a woman having an HIV test from 2006 onwards	[0-1]	Allowed to vary freely.
Relative probability of man having HIV test compared to woman	[0-1]	Men test less frequently in CHiPS and PC data.
Time for someone who is HIV-positive aware (and wants to start ART) to have a CD4 test to determine ART eligibility when ART first becomes available	0-2 years	Assumption.
$p^{background}_{collectHIVresults}(CD4 \geq 200)$, probability collect background HIV test results	0.97	Zambia DHS 2013.
$p^{background}_{collectHIVresults}(CD4 < 200)$, probability collect background HIV test results	1.0	Assume all collect at lower CD4 due to experiencing HIV-related symptoms.
$p^{background}_{collectCD4results}$, probability collect background CD4 test results	0.75-0.95	Lower limit [22]. Upper limit assumption that most collect.
$p^{\text{early ART}}_{die}$, probability die while on early ART	0.08	[20]
$p^{\text{become VS}}$, probability become VS after early ART if do not die	0.9	To get observed values of viral suppression in PC0.
$p^{\text{become VU}}$, probability become VU after early ART if do not die	0.1	So probabilities sum to 1.
$p^{\text{remains VS}}$, probability that a VS woman remains VS for life	0.6	[20]
$p^{\text{stays VS male}}$, relative probability that a VS man remains VS for life (compared to a VS woman)	0.5	Assumption
$p^{\text{VS becomes VU}}$, probability that someone VS eventually becomes VU	0.1	[20]
$p^{\text{VS dropout}}$, probability that someone VS eventually drops out	0.3	[20]
$t^{\text{End VS}}$, time taken for someone VS to drop out/become VU	0.01-6 yrs	Chosen to roughly correspond to the timescale in [20]
$t^{\text{End VU}}$, time taken for someone VU to drop out	0.01-6 yrs	Assumed to have same range as $t^{\text{End VS}}$
$t^{earlyART}$, duration of early ART phase	2 months	Comparable with analysis of ATHENA cohort data from Netherlands (unpublished)
$t^{delayHIVtoCD4}_{background}$, time between background HIV+ test and CD4 test	1 week-6 months	Assumption
$t^{delayHIVtoCD4}_{PopART}$, time between CHiPs HIV+ test and CD4 test	1 week-1 month	Assumption
$t^{\text{CD4 retest}}_{background}$, time between successive CD4 tests when not eligible for ART	0.9-1.1 yrs	Assumption
$t^{\text{CD4 retest}}_{PopART}$, time between successive CD4 tests when not eligible for ART (Arm B)	0.9-1.1 yrs	Assumption
$t^{ART}_{background}$, mean time to start ART through background HIV testing (of those who decide to start ART)	0.4-0.7 yrs	Range from analysis of CHiPs data by country.
t^{ART}_{CHiPs}, mean time to start ART through CHiPs testing	See table below	Analysis of CHiPs follow-up data.

Table P: Cascade-related parameters used in PopART-IBM. Times for initiating ART following a CHiPs visit are in Table Q. *VS=virally suppressed, VU=virally unsuppressed.*

**Round/period**	**Mean time to start ART fast (yrs)** $1/\nu_{fast}$	**Mean time to start ART slow (yrs)** $1/\nu_{slow}$	**Probability of being a fast starter** q
**Zambia**			
Round 1, period 1	0.198	2.711	0.215
Round 1, period 2	0.161	2.378	0.186
Round 1, period 3	0.118	2.086	0.180
Round 1, period 4	0.135	1.620	0.200
Round 1, period 5	0.081	1.282	0.313
Round 1, period 6	0.028	0.854	0.175
Round 2	0.075	1.262	0.226
Round 3	0.030	0.795	0.284
Round 4	0.030	0.795	0.284
**South Africa**			
Round 1, period 1	0.055	1.942	0.076
Round 1, period 2	0.128	2.040	0.078
Round 1, period 3	0.127	1.444	0.075
Round 1, period 4	0.949	15.465	0.915
Round 1, period 5	0.111	1.475	0.202
Round 2	0.109	1.476	0.243
Round 3	0.064	0.659	0.352
Round 4	0.064	0.659	0.352

Table Q: Parameters for time to ART initiation after receiving a positive HIV test result from CHiPs in PopART-IBM. These parameters give a biphasic exponential distribution from which a time to initiate ART is drawn for each individual in the model who receives a positive result from a CHiPs HIV test. Round 4 parameters take the same values as Round 3.

5 Male circumcision

5.1 Types of circumcision

In PopART-IBM we assume that there are two types of male circumcision: traditional circumcision and voluntary male medical circumcision (VMMC). Traditional circumcision is assumed to occur in men prior to entry to the IBM adult population, while VMMC can occur after a man receives an HIV-negative test. VMMC is assumed to reduce susceptibility to HIV in men by Eff^{VMMC}, but not affect infectivity of an HIV-positive man. Traditional circumcision reduces susceptibility in men by Eff^{TMC}. In this setting, based on an analysis from the baseline HPTN 071 (PopART) population cohort data (PC0) we take traditional circumcision to be ineffective in reducing susceptibility ($\text{Eff}^{TMC} = 0$).

In the model, men who are circumcised traditionally are assumed to not receive VMMC in addition.

5.2 Traditional circumcision

From PC0 we use the proportion of men who report being circumcised by a traditional practitioner for the parameter p^{TMC}. Individuals receive traditional circumcision in the model prior to entering the adult population age 14 with probability p^{TMC}. This process of circumcision prior to starting sexual activity is similar to what is found in PC0 in Zambia, where the mean age at circumcision of those who underwent traditional circumcision was 9 years.

5.3 VMMC

VMMC is assumed to occur only in adult men who have received an HIV-negative test (either through background testing or via CHiPs testing, as described in Section 4.6.3). After the test, the probability of receiving circumcision is $p_{circ}^{background}$ and p_{circ}^{CHiPs} respectively depending on whether the HIV test is a background test or CHiPs test. It should be noted that not every man tests in a given year, and these probabilities are conditional on receiving an HIV test of that type (with a negative result) as well as not already being circumcised.

If an individual in the model chooses to receive VMMC, a time for the VMMC is scheduled. For individuals coming from background (non-CHiPs) testing, this is a time $t_{VMMC}^{background}$ in the future; for individuals coming from CHiPs testing this is a time t_{VMMC}^{CHiPs} in the future. One an individual has VMMC scheduled, if they receive another HIV test during the time waiting for VMMC they will not be drawn to see if they will receive VMMC again, to avoid double-scheduling; for example if a man receives a negative CHiPs HIV test result, and chooses to undergo VMMC, if they have a background test shortly after and prior to VMMC they will not have the possibility of scheduling VMMC from that test since they already have a VMMC event scheduled.

Upon getting VMMC an individual initially undergoes a healing period $t_{VMMC}^{Healing}$ of

about 2 weeks, during which they have a modified risk of HIV acquisition, from both the increased susceptibility of the open wound and the reduced coital frequency, resulting in an overall $RR_{\text{circ unhealed}}$, as in [13]. After the end of the healing period they become circumcised with reduction in susceptibility to HIV of Eff^{VMMC}.

5.4 Circumcision coverage over time

VMMC (following a background HIV test) is assumed to have first become available widely in 2010 in Zambia in PopART-IBM: the Zambian Ministry of Health launched the National Male Circumcision Programme in July 2009 [23], and thus 2010 was used to allow a short time to implement the policy at clinic level. VMMC following a CHiPs HIV test is assumed to start at the beginning of the first CHiPs round.

Traditional circumcision is assumed to have been available since the beginning of the simulation.

**Parameter**	**Value**	**Source**
Time when VMMC first became available nationally	2010	Assumption
p^{TMC}, probability of being traditionally circumcised	0.0618 (for community shown); varies by community	PC0 data
Eff^{VMMC}, effectiveness of VMMC in reducing susceptibility	0.6	[24]
Eff^{TMC}, effectiveness of TMC in reducing susceptibility	0.0	PC0 analysis [5]
$RR_{\text{circ unhealed}}$, relative risk of acquiring HIV during VMMC healing phase	0.330	[13]
$p_{circ}^{background}$, probability of being circumcised following background HIV- test	0.4	PC data for individuals in Arm C
p_{circ}^{CHiPs}, probability of being circumcised following CHiPs HIV- test	0.4	Based on analysis of CHiPs uptake data
$t_{VMMC}^{background}$, time between background HIV- test and getting VMMC	0.25-1.0 yrs	Assumption
t_{VMMC}^{CHiPs}, time between CHiPs HIV- test and getting VMMC	0.08-1.0 yrs	Assumption
$t_{VMMC}^{Healing}$, time for VMMC wound to heal	2 weeks	[13]

Table R: Circumcision-related parameters used in PopART-IBM

6 Model validation

A number of steps have been taken when developing PopART-IBM to minimise the risk of bugs in the code. In this section we describe some of the key steps.

Firstly, the model was built modularly, with individual components tested when added. The Valgrind tool[3] was regularly used to check for memory management issues. Different C compilers (GNU and Intel C compilers) were used with different compiler settings to flag unused code, to reduce the likelihood that anything was omitted. The code was compiled on three different platforms (Mac OS X; Windows; Ubuntu) to ensure cross-compatibility. Two modellers (MP, AC) each developed different parts of the code, and the other modeller would check any new code. Additional code walk-throughs were done with three other modellers (WP,RS,CF). The model is available for download and independent scrutiny under the GNU General Public License version 3 at *https://github.com/BDI-pathogens/POPART-IBM*.

To minimize input errors and ensure reproducibility, all model input parameters were analysed using scripts that form part of the model workflow, with warnings whenever the underlying dataset (such as PC0) was changed but the parameter files were not. When PopART-IBM is run, a checking routine is called immediately after parameters are read in, to check that each parameter lies within a 'plausible' range that is specified by hand; in this way, errors caused by the parameters not being in the same order in the parameter files as expected in the reading function are less likely to occur.

Algorithmic errors will generally result in some unexpected state or transition occurring, for example HIV infection to a person who is already HIV-positive (superinfection is not modelled, so this should be impossible in the code), or a list being incorrectly updated such as the list of all living individuals by age containing people who have died. We carry out checks during run-time to ensure the integrity of these lists (for example see section 6.1.1 below). We also check for impossible transitions, for example when infecting an individual that they are not already HIV-positive. We carry out cross-validations when events happen to individuals (for example that when someone dies and we update their list of partners, we check that the partners did indeed appear in these lists as expected). We also track the life histories of random individuals during debugging to ensure that the events happening to them are as expected, for example that they can only become HIV positive when in a serodiscordant partnership, and that they follow an allowed sequence of events for each process. At any point if a check fails the code immediately terminates with an error message.

Finally, key outputs are examined from the model, to see if they are epidemiologically 'plausible' in the view of the modelling team; many of these outputs are presented in this Supplementary Material. Examples of outputs include: HIV prevalence and incidence (see main text Figs 4 and 5); the age distribution of the population (Fig L below); and mean number of current, new and lifetime partners (Fig S). Outputs examined include both the state of the model at given times as well as the transitions between states over time: it is possible to have the same distribution in different ways (for example the

[3]http://valgrind.org

distribution may be static and no changes occur, or people may be rapidly transitioning between states at a rate that means the overall distribution is stationary), so showing the transitions allows us to distinguish between these situations. An example is the distribution of HIV-positives in the ART cascade, where we can examine the churn where individuals drop out of ART and subsequently re-initiate treatment (see Figs Q and R).

For HIV incidence and HIV prevalence, as these are critical outputs, we used two methods with independently created code to calculate them, although the second method has now been removed for speed reasons since the main method has been validated.

Impact, the fraction of infections averted, is the primary output of this modelling study. It is calculated external to the main model code. To validate the impact calculations, impact was separately calculated by two modellers (AC and MP) in two different languages (R and Python).

In the rest of this section we describe in more detail some of the specific checks and validations carried out for specific model processes.

6.1 Checking demographic processes

6.1.1 Cross-validation of population size counts and age list

Within PopART-IBM, the structure containing the population size by sex, age group and sexual activity level is used in many processes, for example in partnership formation. The age list is another fundamental structure, containing a list of currently alive individuals in a patch by sex, divided up into age groups. Processes occurring by age, such as mortality, will generally use the age list as the sampling frame. These structures must be correctly maintained within the model, and we therefore carry out checks to ensure their integrity.

For each timestep, and for each patch, the model counts the population in three different ways. Firstly, as described above, population size by sex, age group and sexual activity level is stored at each time-step in the simulation. This is summed at each timestep to give the total number of men and women in each patch. Secondly, the age list contains the list of people by age group and sex who are currently alive, and we sum the number of people in each age group to get a second method for estimating population size. Finally, an array stores the list of every individual ever alive in that patch; we go through this array counting the individuals who are currently alive by sex. We then compare the three methods by sex to ensure that the totals match. These checks ensure that the population size by sex, age group and sexual activity level variable is consistent with the total population by sex. It also checks the age list contains the correct number of people by sex.

In addition, we output the ages of people in each age group when debugging, to check that individuals are in the correct age group for their age. We also do multiple runs following random individuals and check that they are in the correct age group at all times (focussing on the times when they move from one age group to the next to ensure this happens correctly). Finally we carry out checks in various processes that individuals are alive, to ensure that there are no dead individuals in the age group list.

6.2 Age distribution

The age distribution of the population in the model is a consequence of the fertility and mortality rate parameters derived from UNPD estimates (see Section 2 for details of how this is done). However, given that many processes such as partnership formation are age-dependent, while estimates of cost-effectiveness require some estimate of age and life-years lost through HIV infection, it is important to represent the age distribution of the population accurately. The initial age distribution is an input, and not based on estimates from literature as such data was not recorded at that time. We therefore need to demonstrate that the age distribution has had time to converge by the start of the HIV epidemic so that the age distribution is comparable to available data.

Fig L show the age distribution of the population at four different time points for four randomly chosen calibrated runs (see main text for details of the calibration procedure; these runs were chosen from the 1,000 calibrated runs), compared to the national Zambian estimates from United Nations Population Division (*UNPD*) 2015 World Population Prospects [4]. In 1950 the simulation has not yet had time for the demographics to converge properly, and there is a notable 'bump' in the distribution for individuals aged 45-70. By 1970, when HIV is introduced, this bump has smoothed out as the majority of individuals alive at the start of the simulation will have died, showing the importance of allowing the model to run for a period that is as long as typical life expectancy prior to the introduction of HIV. Generally the model gives similar estimates to those from UNPD in later years, most importantly in 2015, when the trial was underway, although it should be noted that UNPD estimates are national and may not accurately reflect the age distribution within the local communities.

6.3 Population growth

While the previous section shows that the age distribution of the model, stratified by sex, agrees with UNPD estimates over time, we also need to check that the population is growing at a realistic rate. To do this we again use outputs from the four randomly chosen calibrated runs in Zambia, taking the population size of individuals aged 15 and over (UNPD only outputs population size in 5 year age groups, so we choose 15 and over for comparison). We normalise all the model outputs, as well as the UNPD estimates, to start at the same size in 1950.

Fig M shows that the PopART-IBM gives a similar, but very slightly slower growth rate than UNPD estimates, with the modelled 15+ population 1.5-7.8% smaller in 2015 than UNPD estimates (a relative difference in the population growth rate of 0.8-4.5%). Since the PopART-IBM uses its own estimates of AIDS-related mortality, which can differ from the UNAIDS modelled estimates of AIDS-related mortality used in the UNPD estimates, it would not necessarily be expected that the population sizes would match precisely.

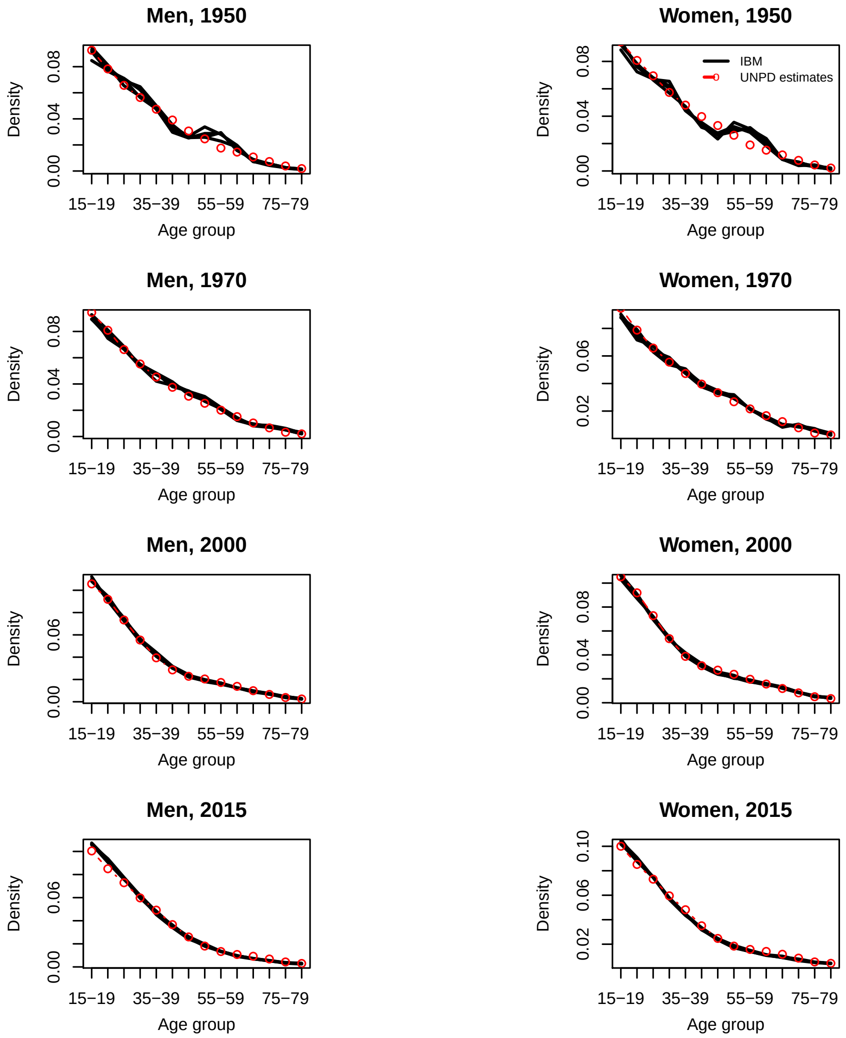

Fig L: Age distribution by sex (left: male; right: female) for 4 randomly chosen calibrated IBM runs in Zambia (black lines) at the following timepoints: 1950, 1970, 2000 and 2015. Red circles show UNPD WPP estimates.

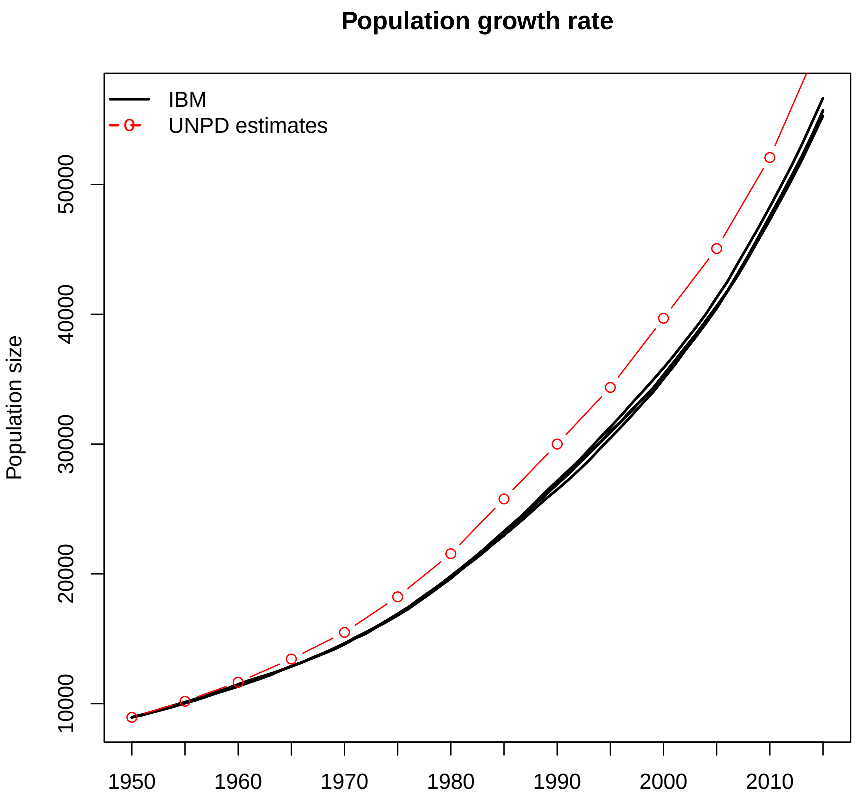

Fig M: Population size over time for 4 randomly chosen calibrated IBM runs in Zambia (black lines). Red line shows UNPD WPP 2015 estimates, normalised to start at the same size in 1950.

6.4 Sexual activity level

Our definition of sexual activity level comes directly from PC0 data (see Section 3.2). Within the model, the proportions in each level at entry into the adult population are fixed by sex. However, HIV may disproportionately affect higher activity levels. Thus high activity level individuals who die from HIV are not replaced on a like-for-like basis.

We therefore need to examine how the population-level distribution of sexual activity level varies over time, particularly during the period corresponding to the HIV epidemic, to understand whether there are large changes in the population-level distribution of sexual activity level that may affect HIV incidence. While it is to be expected that in reality there may be some change in the exact distribution, since on average the most risky individuals will be more likely to acquire HIV and die, large changes in this distribution would signal that assumptions around sexual activity may need to be examined in the model more closely. Fig N shows that in fact the proportion in each activity level is relatively constant over time, with a fairly small decline in the highest activity level, and increase in the lowest activity level, from the 1990s onwards, when HIV-related mortality was affecting particularly those most sexually active. It also shows some of the variability between runs; for the 4 randomly chosen runs, the high sexual activity level group is between 10% and 20% of the population. In section 6.8 we show how the number of sexual partners varies by sexual activity level over time.

6.5 Checking HIV transmission and progression

When an individual is drawn to be infected with HIV, they should be at that point HIV-negative with an HIV-positive partner. We therefore check that they are indeed HIV-negative (and that their CD4 state reflects this), and that they do have at least one HIV-positive partner.

In the PopART-IBM, duration of HIV infection in the absence of antiretroviral therapy depends on the set-point viral load (SPVL) of the individual. For a given SPVL, and in the absence of ART, the time between infection and death will be generated through multiple random draws (see Fig 2 in the main text for the full list of stages). We present here the time between infection and death for individuals who die of AIDS-related illness between the start of the simulation and 2004, when ART becomes available in the simulation. This thus represents a population without access to ART, since once ART becomes available there may be changes to the distribution as, for example, individuals who progress more slowly may have more time to initiate treatment. Note that this population includes seeded infections, who are treated in the same fashion as individuals who have just left acute and early HIV infection (AEHI, see section 4.1 for more details); thus these individuals will survive for a time that is shorter by the duration of the AEHI, but we ignore this since AEHI typically lasts only a few months in the simulation.

Note that this analysis does not present the true distribution of time from infection to AIDS-related death, since it will right-censor individuals who survive beyond 2004, selecting those who survive for less long. However, we include it as it is important in validating a model to understand the underlying dynamics, and duration of infection is

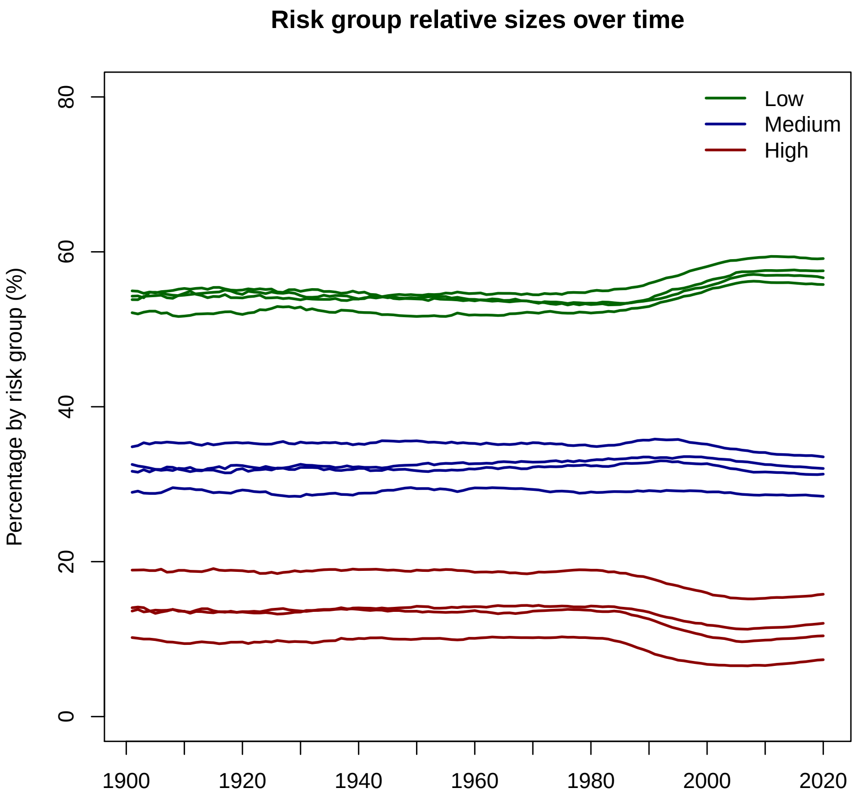

Fig N: Percentage of the population who are in the low, medium and high sexual activity level groups over time, for the 4 randomly chosen calibrated runs.

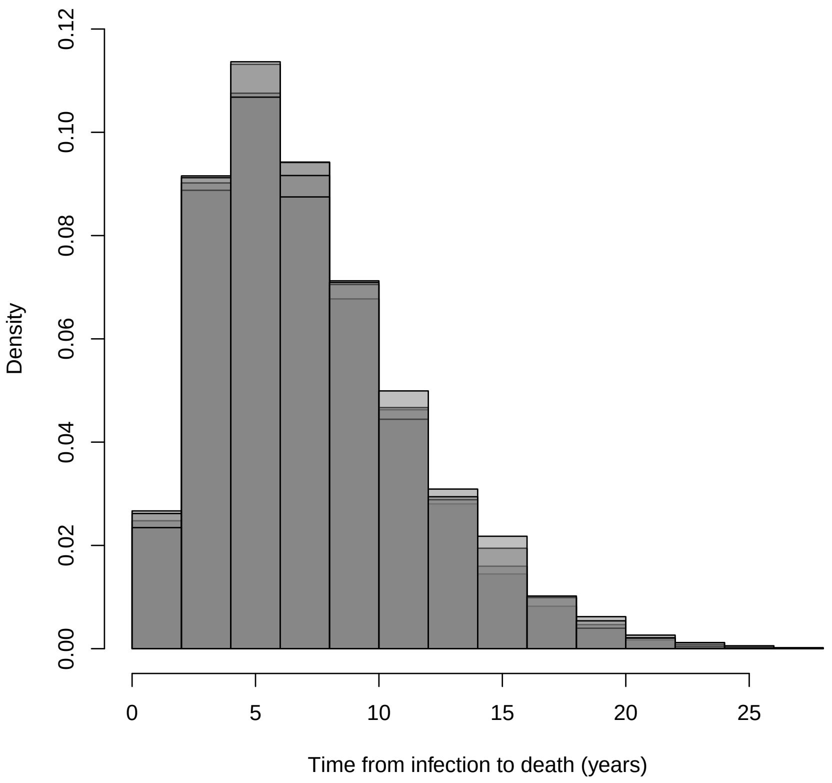

Fig O: Histogram showing the distribution of time from infection to AIDS death for individuals dying by 2004. 4 overlaid histograms are shown, for the 4 randomly chosen calibrated runs.

a critical factor.

Fig O shows the unstratified time from infection to AIDS death for individuals who died by 2004. The figure shows the results from the 4 randomly chosen calibrated runs as overlaid histograms. Thus there is only a small amount of variability between the calibrated runs. Reassuringly there is not a majority who are infected for very short or very long timescales, and this population of individuals who died by 2004 had a mean time to death of 7.2 years.

Fig P shows, for one calibrated run, how the time between infection and AIDS death varies with SPVL category. As expected, individuals with higher SPVL survive for a shorter length of time.

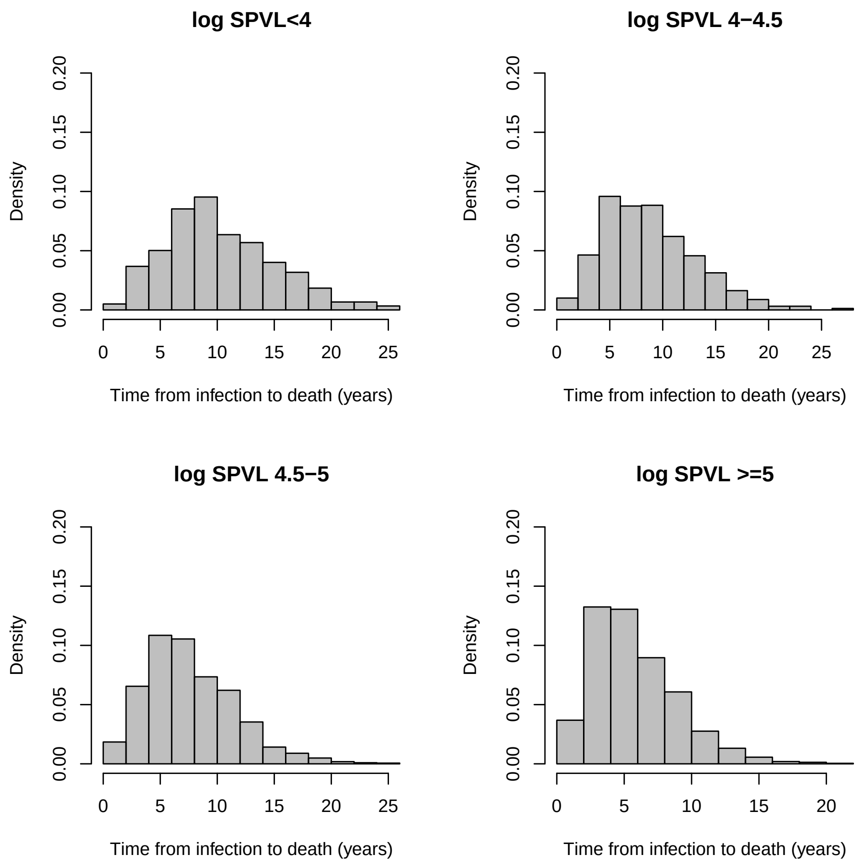

Fig P: Histogram showing the distribution of time from infection to AIDS death for individuals dying in one calibrated run, by set-point viral load category.

6.6 Checking the ART cascade

Whenever a new event is scheduled in the ART cascade, we first check that the individual did not have a prior cascade event already scheduled, as this should not occur. We also check that the individual is still alive.

Fig Q shows the distribution of PLHIV in the ART cascade over time for the four randomly chosen calibrated runs, and Fig R shows the transitions between ART cascade states over time for one of those runs (the others show a similar pattern). As expected, until the advent of HIV testing, individuals are unaware of their HIV status. In 2004 ART becomes available in the model, and individuals can start to initiate ART: at this point a substantial proportion of individuals initiate ART through 'emergency ART' since at this point there will be many individuals with CD4 <200 cells/mm^3 who will have clinical symptoms.

By 2010 the majority of HIV-positive individuals are assumed to know their status, and after this time 'emergency ART' becomes a less important route for ART initiation in the model, with most new ART initiations following a positive HIV test. Discontinuities in the gradient of the line separating 'earlyart' and 'knowposneverart' in Fig Q can be seen at time points reflecting changes in the ART eligibility guidelines, in particular in 2011.

6.7 Checking partnership formation and dissolution

To check for bugs in the partnership formation and dissolution, we performed a series of checks during model validation runs, which are described in this subsection.

6.7.1 Lists of individuals available for partnership formation

Each individual has a fixed maximum number of partners which they cannot exceed at any point in time, which is fixed according to their sexual activity level. If they have not reached this maximum, they are available for partnership formation and appear in a list of available partners (stratified by patch, sex, age and sexual activity level), as many times as appropriate, e.g. once if they have reached their maximum number of partners minus one. The list is initialised at the start of the simulation with all individuals (as there are no partnerships initially), and then updated every time someone enters or leaves the population, and every time a partnership is formed or dissolved. To check this updating is done correctly, we perform the following check: each year, we sweep through all individuals in the population and count their partners and check their maximum number of partners. Based on this information, we check that each individual appears in the list of available partners as appropriate.

6.7.2 Number of partners outside the community

For each individual we record the number of partners outside the community at any time point. This is updated every time a partnership is made or dissolved as appropriate. To check this updating is done correctly, we perform the following check. Each year,

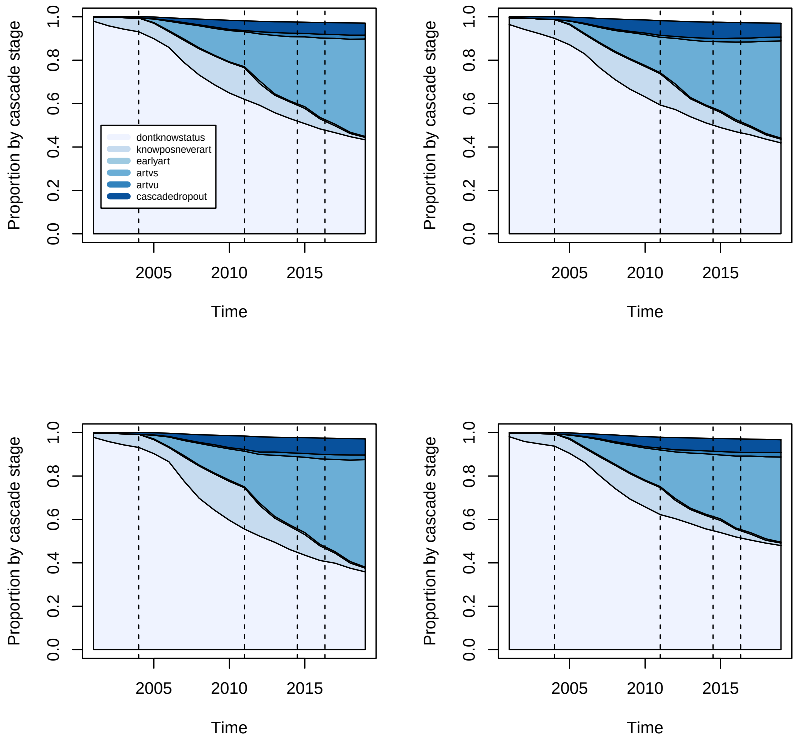

Fig Q: Distribution of PLHIV in the ART cascade over time for the four randomly chosen calibrated runs. Distribution is based on counting the proportion in each cascade state at each timestep. At each timestep, the denominator is equal to the number of currently alive PLHIV.
cascadedropout=dropped out from treatment/care; *artvu*=on ART and virally unsuppressed; *artvs*=on ART and virally suppressed; *earlyart*=early ART (on ART and started at most 2 months ago); *knowposneverart*=PLHIV and aware but never started ART; *dontknowstatus*=PLHIV unaware of status.
Dashed vertical lines show the changes in testing and ART guidelines: in 2004 ART becomes available, and is offered to PLHIV with CD4<200; CD4 guidelines change to CD4<350 (2011), CD4<500 (2014.5) and immediate ART regardless of CD4 (2016.33).

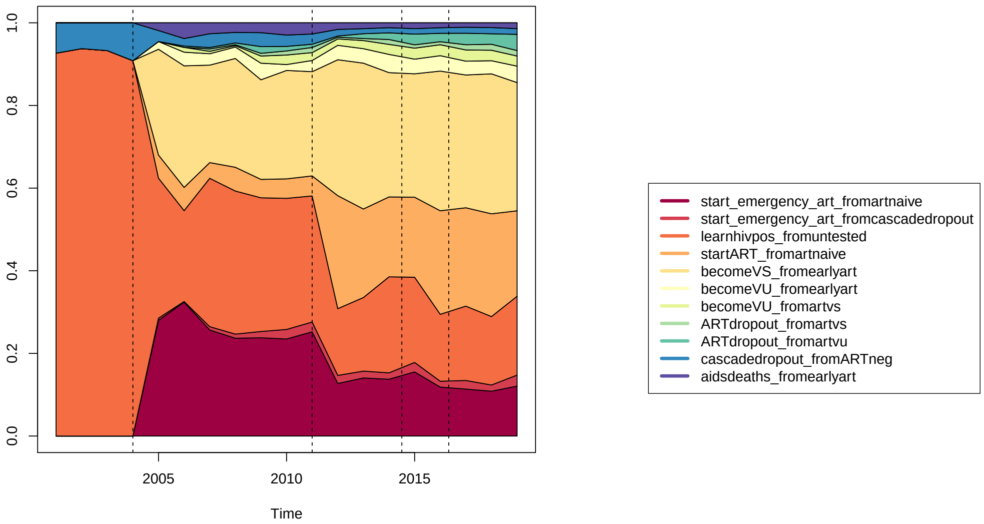

Fig R: Changes in transitions between ART cascade states of PLHIV over time for one randomly chosen calibrated run. Results for the other three calibrated runs are similar. Distribution is based on counting the total number of cascade transitions occurring during the calendar year (a transition is a change from one cascade state to another as shown in Fig K).

Transitions are: *start_emergency_art_fromcascadedropout/artnaive*=start emergency ART when dropped out/never started ART; *learnhivpos_fromuntested*=become aware of HIV status; *startART_fromartnaive*=start ART (note that this can be someone who dropped out as well as someone ART-naive); *becomeVS_fromearlyart*=become on ART and virally suppressed after being in early ART; *becomeVU_fromartvs/earlyart*=become on ART but virally unsuppressed after being on ART and virally suppressed/being in early ART; *ARTdropout_fromartvu/artvs*=stop ART and drop out of care when on ART and VS/on ART and VU; *cascdropout_fromARTneg*=drop out of cascade upon learning HIV+ prior to starting ART for the first time; *aidsdeaths_fromearlyart*=HIV-related death during early ART.

Dashed vertical lines show the changes in testing and ART guidelines: in 2004 ART becomes available to PLHIV with CD4<200; CD4 guidelines change to CD4<350 (2011), CD4<500 (2014.5) and immediate ART regardless of CD4 (2016.33).

we sweep through all individuals in the population and loop through their partners and count how many are outside the community. We then check that this is consistent with the recorded number of partners outside the community for that individual.

6.7.3 Rates of partnership formation

We output, each year, the average number of new partners made by individuals in that year, disaggregated by sexual activity level. We check that this is consistent with the relative rates of partnership formation in each sexual activity level. We similarly check the average number of new partners in the last year made by individuals in the low sexual activity group, stratified by sex and age is consistent with the value calculated from the input parameters. Finally we output, each year, the average number of new partners in the last year made by individuals in the low sexual activity group, with partners in a different community, stratified by sex and age, and check this is consistent with the value calculated from the input parameters.

Note this average number of new partners needs some time to reach equilibrium, and then equilibrium is affected by HIV, so checking is done between reaching equilibrium and HIV starting. Also note that because of balancing issues (see Section 3.3.2) we may not expect exactly the same results.

6.7.4 Assortativity of partnerships

We carry out the following tests to validate that the model is creating partnerships in the correct way by age and sexual activity level.

- **Assortativity by age (at partnership formation)** Every year, we output the ages of the individuals in all newly formed partnerships, building an age assortativity matrix which we can then compare to the PC0 data that was used to determine the input parameter values. Again note there may be some differences because of balancing. Note this includes all partnerships between and within patches without distinction between the two, since the age assortativity matrices used are independent of partners location

- **Assortativity by age (partners at a given time point)** Every year, we sweep through all alive individuals and check their age group and the age group of their partners, and compare with PC0.

- **Assortativity by sexual activity level (at partnership formation)** Every year, we output the activity class of all newly formed partnerships, building a sexual activity level assortativity matrix which we can then compare to the input assortativity parameter. Again note there may be some differences because of balancing. Note also that this includes all partnerships between and within patches without distinction, since the sexual activity assortativity used is independent of the partners' locations.

- **Assortativity by sexual activity level (partners at a given time point)** Every year, we sweep through all individuals who are currently alive, and check their sexual activity level and the sexual activity level of their partners.

Validity of assortativity by age is checked visually by comparing the input age mixing matrices with the age mixing matrix obtained in the PopART-IBM at partnership formation, and also cross-sectionally as described above. Similarly, validity of assortativity by sexual activity level is checked visually by comparing the input assortativity parameter with the sexual activity level mixing matrix obtained in the PopART-IBM at partnership formation, and also cross-sectionally as described above.

6.7.5 Partnership duration by activity class and within vs between communities, as drawn at partnership formation.

We output all duration of partnerships (as drawn at partnership formation so ignoring premature dissolution due to death) between two high sexual activity level individuals overall, as well as in the same community (patch 0) and in different communities (patches 0 and 1), and compare the distributions of the duration for each with the data from PC0 used to derive the input parameter values. We do the same for medium-medium and low-low sexual activity level partnerships. We show the distribution from PopART-IBM versus PC0 data distributions in Figs I and J in Section 3.6.1.

6.8 Validating partnership outputs

It is important to assess how partnership numbers vary between sexual activity levels and over time within the simulation. Fig S shows how the number of current, lifetime, and new (i.e. formed within the past 12 months) partners varies over time for individuals currently alive in the simulation. Results are shown for the four randomly chosen calibrated runs.

The mean number of lifetime partners has reached equilibrium by about 60 years after the start of the simulation, prior to the introduction of HIV. This supports the assertion that the partnership processes have stabilized by the start of the HIV epidemic. Once HIV is introduced, the mean number of lifetime partners of high activity levels individuals decreases, due to their reduced lifespan caused by HIV-related mortality. The same is true to a lesser extent of the mean number of current partners in this group, although of note the number of new partners in this group remains stable (or possibly even increases slightly as the remaining high activity level individuals are more ‘in demand’), showing that these decreases in current/lifetime partners are not caused by reductions in partnership formation.

Across sexual activity levels, we see that low activity level individuals have an average of one or two lifetime partners; medium activity level have 2-8 partners, and high activity level individuals may have over 20 lifetime partners, and several concurrent ones.

Finally, as may be expected by the large population size, there is relatively little stochasticity within runs, and the difference in each measure between runs is larger than within runs.

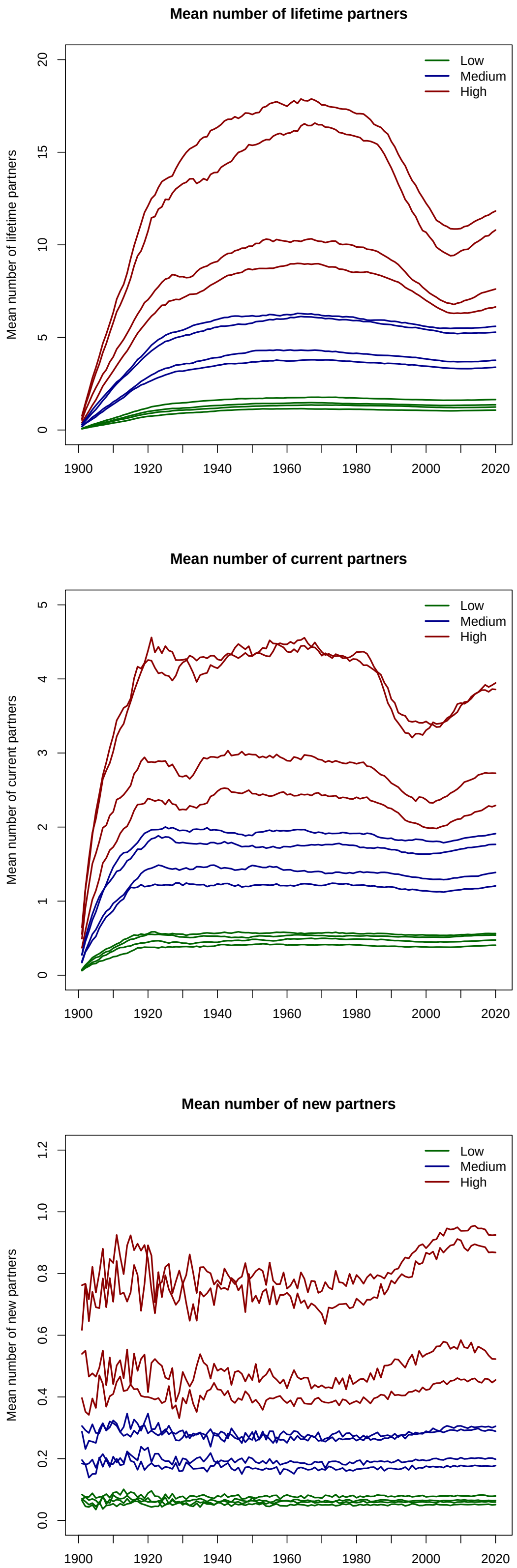

Fig S: Rows show mean number of lifetime (top), current (middle), and new (bottom; i.e. partnerships formed within the past 12 months) partners of individuals currently alive in the simulation, plotted over time by sexual activity level for the 4 randomly chosen calibrated runs.

7 Supplementary Results

7.1 Uncalibrated runs and epidemic diversity

We first consider how PopART-IBM performs without calibration: this allows us to examine to what extent the prior ranges imposed on the parameters constrain the space of possible epidemic trajectories. A selection of 200 parameter sets was generated from the specified prior ranges using Latin hypercube sampling, and the model was run with each of them them. Fig T shows HIV prevalence over time for these 200 runs. This suggests that the model, with the given prior parameter ranges, is capable of simulating diverse epidemic trajectories, with higher/lower peak HIV prevalence and faster/slower growing epidemics all possible.

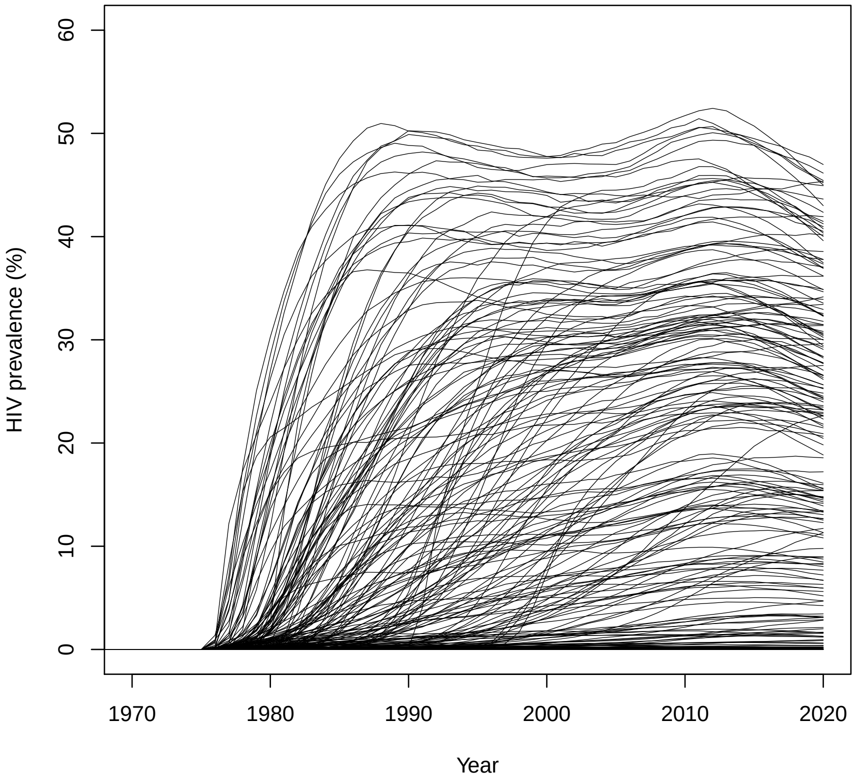

Fig T: HIV prevalence for 200 uncalibrated runs.

7.2 Posterior parameter plots

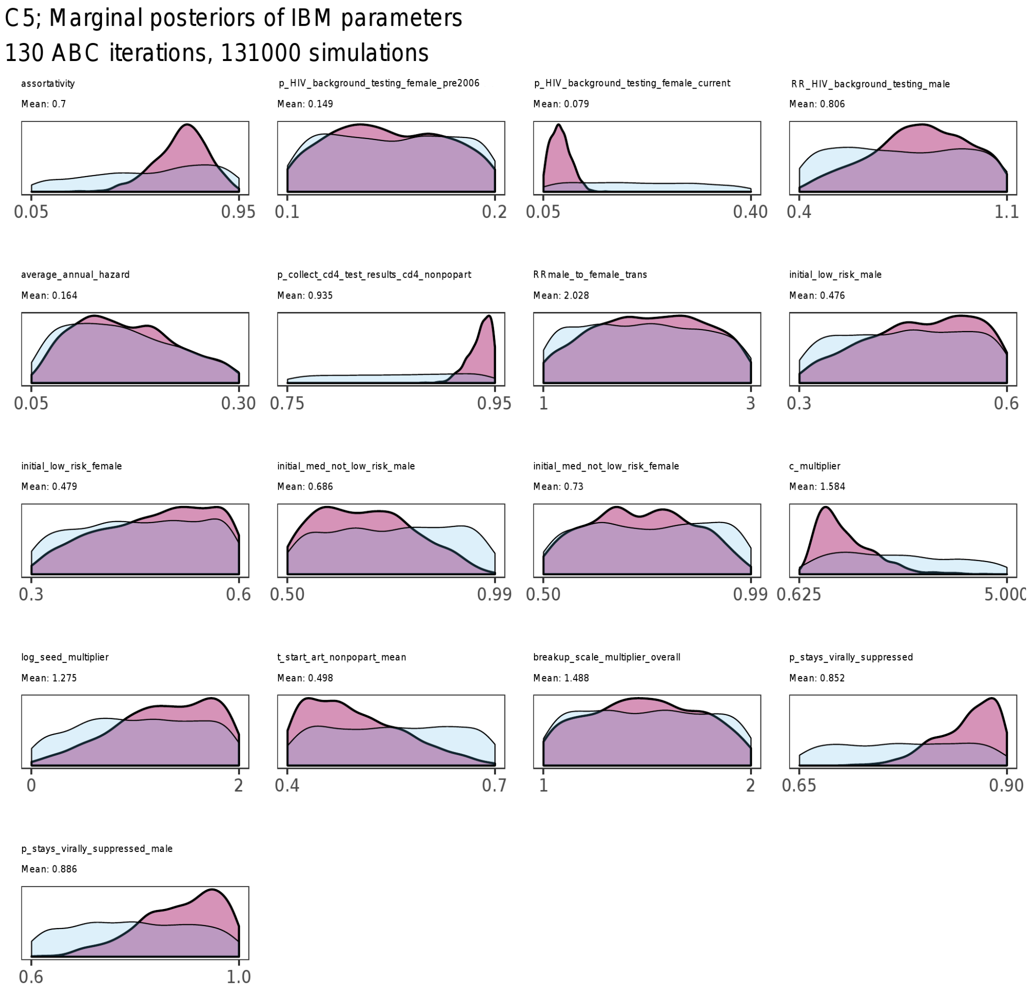

Fig U: Marginal posterior distributions for the 17 calibrated parameters, based on 131 ABC iterations and 131,000 simulations.

Fig U shows the marginal posterior distribution of each parameter. In the setting given, assortativity, the baseline annual probability of a women having an HIV test in a given year after 2006, the probability that an individual not in PopART collects CD4 test results and therefore join pre-ART care, the multiplier to account for potential over/under-reporting of number of sexual partners, the probability that a woman stays virally suppressed after the initial ART phase, and the multiplier for the probability that a man stays virally suppressed after the initial ART phase compared to a women were most informed by the calibration process.

References

[1] M. Beaumont, J.-M. Cornuet, J.-M. Marin, and C. P. Robert. Adaptive approximate Bayesian computation. *Biometrika*, 96:983–990, 2009.

[2] M. Lenormand, F. Jabot, and G. Deffuant. Adaptive approximate bayesian computation for complex models. arXiv:1111.1308 [math.ST], 2015.

[3] M. Pickles, M. C. Boily, P. Vickerman, C. M. Lowndes, S. Moses, J. F. Blanchard, K. N. Deering, J. Bradley, B. M. Ramesh, R. Washington, R. Adhikary, M. Mainkar, R. S. Paranjape, and M. Alary. Assessment of the population-level effectiveness of the Avahan HIV-prevention programme in South India: a preplanned, causal-pathway-based modelling analysis. *Lancet Glob Health*, 1(5):e289–299, Nov 2013.

[4] Department of Economic United Nations and Population Division Social Affairs. World population prospects: The 2015 revision, dvd. `http://esa.un.org/unpd/wpp/Download/Standard/Population/`, 2015. Accessed 27 May 2016.

[5] M. Pickles, A. Cori, S. Floyd, H. Ayles, N. Beyers, P. Bock, S. Fidler, R. Hayes, and C. Fraser. Exploring the effectiveness of traditional circumcision practices in preventing hiv. In *CROI 2016*, Boston, 2016.

[6] `https://www.cia.gov/library/publications/the-world-factbook/fields/2018.html`. Accessed 16 June 2016.

[7] Department of Economic United Nations and Population Division Social Affairs. World population prospects: The 2015 revision, methodology of the united nations population estimates and projections, working paper no. esa/p/wp.242. `http://esa.un.org/unpd/wpp/publications/Files/WPP2015_Methodology.pdf`, 2015. Accessed 7 June 2016.

[8] Geoffrey P. Garnett and Roy M. Anderson. Balancing sexual partnership in an age and activity stratified model of hiv transmission in heterosexual populations. *Mathematical Medicine and Biology*, 11(3):161–192, 1994.

[9] Bart Burington, James P Hughes, William L H Whittington, Brad Stoner, Geoff Garnett, Sevgi O Aral, and King K Holmes. Estimating duration in partnership studies: issues, methods and examples. *Sexually Transmitted Infections*, 86(2):84–89, 2010.

[10] C. Fraser, T. D. Hollingsworth, R. Chapman, F. de Wolf, and W. P. Hanage. Variation in HIV-1 set-point viral load: epidemiological analysis and an evolutionary hypothesis. *Proc. Natl. Acad. Sci. U.S.A.*, 104(44):17441–17446, Oct 2007.

[11] D. Donnell, J. M. Baeten, J. Kiarie, K. K. Thomas, W. Stevens, C. R. Cohen, J. McIntyre, J. R. Lingappa, C. Celum, C. Celum, A. Wald, J. Lingappa, J. M. Baeten, M. Campbell, L. Corey, R. W. Coombs, J. P. Hughes, A. Magaret, M. J.

McElrath, R. Morrow, J. I. Mullins, D. Coetzee, K. Fife, E. Were, M. Essex, J. Makhema, E. Katabira, A. Ronald, S. Allen, K. Kayitenkore, E. Karita, E. Bukusi, C. Cohen, S. Allen, W. Kanweka, S. Allen, B. Vwalika, S. Kapiga, R. Manongi, C. Farquhar, G. John-Stewart, J. Kiarie, S. Allen, M. Inambao, S. Delany-Moretlwe, H. Rees, G. de Bruyn, G. Gray, J. McIntyre, and N. R. Mugo. Heterosexual HIV-1 transmission after initiation of antiretroviral therapy: a prospective cohort analysis. *Lancet*, 375(9731):2092–2098, Jun 2010.

[12] S. E. Bellan, J. Dushoff, A. P. Galvani, and L. A. Meyers. Reassessment of HIV-1 acute phase infectivity: accounting for heterogeneity and study design with simulated cohorts. *PLoS Med.*, 12(3):e1001801, Mar 2015.

[13] A. Cori, H. Ayles, N. Beyers, A. Schaap, S. Floyd, K. Sabapathy, J. W. Eaton, K. Hauck, P. Smith, S. Griffith, A. Moore, D. Donnell, S. H. Vermund, S. Fidler, R. Hayes, C. Fraser, Y. Agyei, M. Baldwin, M. Barnes, V. Bond, D. Burns, N. Chishinga, V. Cummings, L. Emel, S. Eshleman, P. Godfrey-Faussett, E. Greene, J. Hargreaves, T. Headen, L. Horn, P. Kim, E. Piwowar-Manning, K. McCarthy, M. Musheke, A. Mwango, A. Mwinga, M. Muyoyeta, M. Simwinga, K. Shanaube, D. Watson-Jones, S. Wolf, and R. White. HPTN 071 (PopART): a cluster-randomized trial of the population impact of an HIV combination prevention intervention including universal testing and treatment: mathematical model. *PLoS ONE*, 9(1):e84511, 2014.

[14] M. C. Boily, R. F. Baggaley, L. Wang, B. Masse, R. G. White, R. J. Hayes, and M. Alary. Heterosexual risk of HIV-1 infection per sexual act: systematic review and meta-analysis of observational studies. *Lancet Infect Dis*, 9(2):118–129, Feb 2009.

[15] M. S. Cohen, Y. Q. Chen, M. McCauley, T. Gamble, M. C. Hosseinipour, N. Kumarasamy, J. G. Hakim, J. Kumwenda, B. Grinsztejn, J. H. Pilotto, S. V. Godbole, S. Chariyalertsak, B. R. Santos, K. H. Mayer, I. F. Hoffman, S. H. Eshleman, E. Piwowar-Manning, L. Cottle, X. C. Zhang, J. Makhema, L. A. Mills, R. Panchia, S. Faesen, J. Eron, J. Gallant, D. Havlir, S. Swindells, V. Elharrar, D. Burns, T. E. Taha, K. Nielsen-Saines, D. D. Celentano, M. Essex, S. E. Hudelson, A. D. Redd, and T. R. Fleming. Antiretroviral Therapy for the Prevention of HIV-1 Transmission. *N. Engl. J. Med.*, 375(9):830–839, Sep 2016.

[16] A. Cori, M. Pickles, A. van Sighem, L. Gras, D. Bezemer, P. Reiss, and C. Fraser. CD4+ cell dynamics in untreated HIV-1 infection: overall rates, and effects of age, viral load, sex and calendar time. *AIDS*, 29(18):2435–2446, Nov 2015.

[17] S. Bonhoeffer, C. Fraser, and G. E. Leventhal. High heritability is compatible with the broad distribution of set point viral load in HIV carriers. *PLoS Pathog.*, 11(2):e1004634, Feb 2015.

[18] M. S. Cohen, C. L. Gay, M. P. Busch, and F. M. Hecht. The detection of acute HIV infection. *J. Infect. Dis.*, 202 Suppl 2:S270–277, Oct 2010.

[19] Zambia Ministry of Health. https://www.k4health.org/sites/default/files/ct_guidelines_march_2006.pdf. Accessed 20 November 2014.

[20] M. J. Vinikoor, J. Joseph, J. Mwale, M. A. Marx, F. M. Goma, L. B. Mulenga, J. S. Stringer, J. J. Eron, and B. H. Chi. Age at antiretroviral therapy initiation predicts immune recovery, death, and loss to follow-up among HIV-infected adults in urban Zambia. *AIDS Res. Hum. Retroviruses*, 30(10):949–955, Oct 2014.

[21] T. B. Hallett and J. W. Eaton. A side door into care cascade for HIV-infected patients? *J. Acquir. Immune Defic. Syndr.*, 63 Suppl 2:S228–232, Jul 2013.

[22] C. Mugglin, J. Estill, G. Wandeler, N. Bender, M. Egger, T. Gsponer, and O. Keiser. Loss to programme between HIV diagnosis and initiation of antiretroviral therapy in sub-Saharan Africa: systematic review and meta-analysis. *Trop. Med. Int. Health*, 17(12):1509–1520, Dec 2012.

[23] Ministry of Health, Republic of Zambia. National voluntary medical male circumcision (VMMC) communication and advocacy strategy 2012-2015. https://www.malecircumcision.org/sites/default/files/document_library/Zambia_Ministry_of_Health_VMMC_Communications_and_Advocacy_Strategy_April_2012.pdf, 2012. [Online; accessed 24-November-2009].

[24] N. Siegfried, M. Muller, J. J. Deeks, and J. Volmink. Male circumcision for prevention of heterosexual acquisition of HIV in men. *Cochrane Database Syst Rev*, 15(2):CD003362, 2009.

List of Figures

A Annual mortality for men in Zambia by age group 20
B Annual mortality for women in Zambia by age group 21
C Annual mortality for men in South Africa by age group 22
D Annual mortality for women in South Africa by age group 23
E Definition of sexual activity levels in PopART-IBM 27
F HIV prevalence by sex, sexual activity level and age group for Zambia and South Africa in PC0 data 28
G Rate of partnership formation inside and outside the community, by age, activity class and sex for Zambia 35
H Rate of partnership formation inside and outside the community, by age, activity class and sex for South Africa 36
I Observed and fitted duration of partnerships so far for Zambia 40
J Observed and fitted duration of partnerships so far for South Africa . . . 41
K Schematic of the HIV care cascade in the model. 53
L Age distribution by sex for 4 randomly chosen calibrated IBM runs in Zambia 65
M Population size over time for 4 randomly chosen calibrated IBM runs in Zambia 66
N Percentage of the population who are in the low, medium and high sexual activity level groups over time, for the 4 randomly chosen calibrated runs. 68
O Histogram showing the distribution of time from infection to AIDS death for individuals dying by 2004 69
P Histogram showing the distribution of time from infection to AIDS death for individuals dying in one calibrated run, by set-point viral load category. 70
Q Distribution of PLHIV in the ART cascade over time for the four randomly chosen calibrated runs 72
R Changes in transitions between ART cascade states of PLHIV over time for one randomly chosen calibrated run 73
S Mean number of lifetime, current, and new partners of individuals currently alive in the simulation, plotted over time by sexual activity level for the 4 randomly chosen calibrated runs 76
T HIV prevalence for 200 uncalibrated runs 77
U Marginal posterior distributions for the 17 calibrated parameters 78

List of Tables

A List of epidemiological characteristics of each adult individual stored by PopART-IBM. . . . 8
B Parameters related to initialization of the population in PopART-IBM . . 13
C Fertility rate for Zambia over time 15
D Fertility rate for South Africa over time 16
E Years for which UNPD mortality estimate is used in regression model . . . 19
F Country, sex and age-group specific mortality parameters. . . . 24
G Partnership-related parameters used in PopART-IBM. . . . 26
H Proportion of population in each sexual activity level by sex 29
I Age mixing matrix for men in Zambia 30
J Age mixing matrix for women in Zambia 30
K Age mixing matrix for men in South Africa 31
L Age mixing matrix for women in South Africa 31
M Parameters related to initializing HIV in PopART-IBM. . . . 43
N HIV transmission-related parameters used in PopART-IBM 45
O HIV progression-related parameters used in PopART-IBM 49
P Cascade-related parameters used in PopART-IBM 58
Q Parameters for time to ART initiation after receiving a positive HIV test result from CHiPs in PopART-IBM 59
R Circumcision-related parameters used in PopART-IBM 61
