# Supplementary material for: PopART-IBM, a highly efficient stochastic individual-based simulation model of generalised HIV epidemics developed in the context of the HPTN 071 (PopART) trial
Source: PLoS Comput Biol. 2021 Sep 2;17(9):e1009301. doi: 10.1371/journal.pcbi.1009301 (PMC8478209; doi:10.1371/journal.pcbi.1009301)
Supplement: Attachment — Submitted filename: Reviewer comments_01.pdf [file pcbi.1009301.s002.pdf]

The is an important article aimed to use the Bayesian inference framework on the computationally efficient PopART-IBM to calibrates age- and sex-stratified data on HIV prevalence, HIV status, ART status & viral suppression then project the prevalence and incidence (in the absence of trial intervention) for an HPTN 071 study community in Zambia. The analysis is reasonable and the paper is written and presented well though I have a few comments.

Comment 1:

Under model parameterization and calibration, the authors could possibly present some key mathematical expressions from the supplementary in the main manuscript for ease of readability and flow. In case a reader is interested in understanding further details then can easily refer to the supplementary document.

Comment 2:

With the mathematical expressions the authors can then consider summarizing the Methods section again for ease of readability.

Comment 3:

Figure S4.1 can also be included in the main manuscript.

Comment 4:

Figure S7.3 does not seem to be in the supplementary document.

Comment 5:

The supplementary document is huge (about 84) pages. Can the authors consider reducing it to what is extremely necessary for reference?
