# Supplementary material for: PopART-IBM, a highly efficient stochastic individual-based simulation model of generalised HIV epidemics developed in the context of the HPTN 071 (PopART) trial
Source: PLoS Comput Biol. 2021 Sep 2;17(9):e1009301. doi: 10.1371/journal.pcbi.1009301 (PMC8478209; doi:10.1371/journal.pcbi.1009301)
Supplement: Attachment — Submitted filename: Review of PopART-IBM for PLOS Computational Biology.pdf [file pcbi.1009301.s003.pdf]

Major revisions, (general):

- This article appears to be improperly formatted for *PLOS Computational Biology*. The authors present a novel mathematical model (PopART-IBM) for increased predictive power of epidemiological models relating to HIV, specifically for HPTN 071. Such a model could be both useful and of interest to the scientific community and could possibly lead to more informed decision-making, etc. However, the authors delegate crucial details, (e.g., much of Section S3, including Table S3.1, and Section S6) of their new model to the Supplementary Material, providing only generic information in the main article. I think that it is fine to leave excessive details such as those comprising Sections S2, S4, and Section S5 in the Supplementary Material, but the mathematical basis behind, parameterisation of, and validation of the novel model being presented should absolutely be laid out within the main paper. A recently published example in *PLOS Comp. Biol.* of authors presenting a new model with both good graphical representations and mathematical foundations of their model within the actual article is: https://doi.org/10.1371/journal.pcbi.1007606. As *PLOS Comp. Biol.* does not confine authors to the strict page limits of journals such as *PNAS* and *Nature*, there is really no excuse to not provide these details within the main paper.
- In the same vein as the above comment, the summary statistics chosen by the authors should be clearly presented, perhaps in a table, and discussed in the main paper. The authors have alluded to the computational costliness and complexity of running such models as theirs, so I assume this is the reason behind using ABC rather than a full likelihood description. However, the authors should then stress the importance of the choice and dimensionality of their summary statistics within the main article.
- In the .pdf file provided for reviewers, the quality of the figures in the main article is very poor, with very low resolution. I can barely read the numbers on the x-axes of the graphs in Figures 3a and 3b. A duplicate of Figure 1 appears in the Supplementary Material (S1) and has better resolution. In fact, all of the figures in the Supplementary Material are of better quality, so I assume the way the .pdf file of the main paper was generated messed up the figures. That said, the authors should ensure good quality of their figures upon final submission.

Major revisions, (specific):

- Paragraph 2 in the Background section states, “Phylogenetic studies are starting to provide new quantitative insights into transmission dynamics” without a citation (e.g., doi: 10.1093/ve/vey037). As an aside, I would recommend removing the “are starting to” part of this sentence as even phylodynamic (joint inference of phylogenetic and epidemiological information) models have been around since 2004, (doi: 10.1126/science.1090727), and there have been many new phylodynamic models, extensions of preexisting ones, and data analyses using them since then.
- Top of Page 9, in the **HIV transmission** section, there is the statement, “The relative transmission rates are determined from published literature on HIV transmission in serodiscordant couples in sub Saharan Africa,” with no citation.
- Why did the authors choose the distance measure of 9% for using APMC-ABC, as opposed to, for example, 5%? How does the model perform under other values?

Presumably the smaller the number, the better the statistics, but also the longer the model will need to run. I urge the authors to give us an idea of how different values affect both their model performance and computation time.

- I cloned the git repository linked in the paper (https://github.com/BDI-pathogens/POPART-IBM) to test the authors' code. (Note: gcc and gsl must be pre-installed, which are not part of the instructions in the README in the GitHub repository. For example, I received the error shown below when attempting to compile the simulation code with both macOS and Ubuntu, as I had not yet installed the GNU Scientific Library.)

```
make clean; make all
rm -f constants.o demographics.o hiv.o init.o input.o interventions.o output.o partnership.o utilities.o checks.o memory.o fitting.o debug.o pc.o simul.o main.o  popart-simul.exe
gcc -g -Wall -fmessage-length=0 -I/usr/local/include -O0 -c constants.c -o constants.o
In file included from constants.c:17:
./constants.h:32:10: fatal error: 'gsl/gsl_rng.h' file not found
#include <gsl/gsl_rng.h>
         ^~~~~~~~~~~~~~~
1 error generated.
make: *** [constants.o] Error 1
```

  - After installing gsl via Homebrew, the code compiled, but running the executable (popart-simul.exe) produced another error (shown below), both when using macOS and Ubuntu. It seems something is going wrong with the parameter file. I tried to explicitly specify the parameter values as given in the README ("nruns=10" and "CF=0") rather than calling the variables but this produced the same error. With a little more prodding, I saw something about an argument labelled "n_runs", but by this point I decided that further debugging was the responsibility of the authors.

```
./src/popart-simul.exe $output_dir $nruns $CF
Offset for PC is 0
Cannot open python_seed.txtLINE 2015; FILE input.c
python --version
Python 2.7.10
```

  - The Supplementary Material states in Section S6 that the code should be accessible to Mac OS X, Windows, and Ubuntu users. But it appears that either more detailed instructions or possibly some debugging is required in order to run POPART-IBM/ reproduce the authors' results.

Minor revisions:

- I noticed both British spelling (notably, "parameterised" and "parameterise") and American spelling ("parameterized", "parameterization", and "parameterizing"). Either is fine, but consistency makes the paper look a bit tidier.
- Formatting inconsistency of the superscripts in Supplementary Material (examples: "early ART" on page 57 and "End VU" on page 58 have larger font and are not italicised like the other superscripts around them).
- The **Parameter** column in Table S4.4 is a bit difficult to read.
