# Supplementary material for: PopART-IBM, a highly efficient stochastic individual-based simulation model of generalised HIV epidemics developed in the context of the HPTN 071 (PopART) trial
Source: PLoS Comput Biol. 2021 Sep 2;17(9):e1009301. doi: 10.1371/journal.pcbi.1009301 (PMC8478209; doi:10.1371/journal.pcbi.1009301)
Supplement: Attachment — Submitted filename: review4.docx [file pcbi.1009301.s004.docx]

**Main comments**

This paper describes the features of the PopART-IBM model, which simulates the HIV-epidemic in Zambia in the context of the PopART study. Below are my comments:

1) The strength of the model is its high computational efficiency, as one simulation takes less than 1 minute. However, the authors should explain in more details how this short computational time influence the calibration process. Are the other IBMs that the authors describe in the discussion also able to estimate some of their parameters or is their computational time too long for that? If they are able, how many parameters do they estimate? In addition, it would be interesting to know how much the whole calibration process takes, and not only how much one simulation takes.

2) Another strength of the model is that it reproduced the PopART intervention and its impact on the study community (patch 0) and its proximate surrounding area (patch 1). However, the authors did not use this feature of the model to compare the impact of different interventions (PopART vs no intervention) on both patches. The authors should explain why (see also my second comment in the *Results* section). Moreover, the PopART intervention could be quickly described in the main manuscript as you did in the supplementary material S1.8.

3) The model does not differentiate between the different ART regimens (NNRTI-based, DTG-based). It would be good to acknowledge that and explain why such simplification might not drastically change the results (did Rwanda already start DTG?).

4) The authors should also clarify the research question. Does this paper aim at showing the high computational efficiency of the model or does it aim at describing model results about the impact of PopART intervention on the HIV-epidemic? Currently, it seems to me that the paper mixes a bit the two aims.

5) I did not find specific information on both prior and posterior distributions of the 17 calibrated parameters. See also my second comments in the *Discussion* section.

**Other comments:**

- It would have been much easier to review this paper with line and page numbers.

- The quality of the pictures is quite low, making them hard to read.

**Background:**

- 2nd paragraph: I am not sure to understand what granular exactly means here in the context of mathematical modelling. Consider rephrase it.

- You should also add the limitations of IBM models, e.g. that such complex models are difficult to parametrize/calibrate and that's why simpler model, such as compartmental models, are preferred, as they are easier to control and do not make unverifiable assumptions.

**Methods:**

- Figure 1: To me, the Figure 1 is far too simple and does not reflect the complexity of the model. I know that it is impossible to summarize all the model features in one figure but I expected more details. For example, to explain how your IBM works, the author could use the same representation as in Figure 5 (but with less individuals), and show the trajectory of a few hypothetical individuals (split by patches) and represent the following events: birth, deaths, partnership (by linking two individuals), HIV infections, care cascade, interventions (testing, PopART intervention). You could use different colors for the different features (as you currently do in Figure 1 with the 6 features), keep the "puzzle" that explains the 6 features next to the representation of your IBM.

- The author could make a wider use of the extended supplementary material. For example, when describing how partnership is modelled, the author could add a reference to the section in the supplementary material that describes that in details.

- The author could add a sentence explaining how the new partnerships are formed (and which individuals are selected for each new partnership) or at least add a reference to the supplementary material.

- The Model section could be split in two subsection: 1) Model structure, 2) Model parametrization/simulation, for more clarity.

- The authors could add a table that summarizes the 17 parameters that are estimated through the calibration process. In the current version, it is unclear which parameters are estimated and why the authors chose these parameters.

- In the "Measuring computational efficiency" section, the authors explain that the simulations start in 1900 and end in 2020, corresponding to 120 years. In the Results section, it is written that they model 130 years of the epidemic. Why is it different? In addition, it is not clear why the model has to start so early in 1900. As far as I understood, the authors need some simulation time before the start of the HIV epidemic to estimate the age distribution of the population (i.e. to have a "stabilized" age distribution). Why cannot the authors simply take the known age distribution at the beginning of the epidemic and start the model at that time?

**Results:**

- The authors claim that the simulation lasts about 30 seconds. In the "Calibration" subsection, they say that the ABC algorithm required 131,000 simulations. This would correspond to 131,000 times 30 seconds, i.e. more than 45 days. Does the model really takes so much time? Some clarity is needed here. Overall, it would be useful to know how much time the model takes to estimate the 17 parameters, and not only how much time it needs to run one simulation (i.e. 30 seconds).

- Figure 4 does not bring anything new, in the sense that you do not use the features of the model (i.e. considering the impact of PopART intervention). It would be much more interesting to present model estimations of the impact of the different "packages" of interventions as described in the PopART. Moreover, it would also be interesting to split the results by patches.

**Discussion:**

- The authors wrote: "In addition, model predictions for the proportion of PLHIV on ART are somewhat lower than the data, though this may be expected due to social desirability biases when individuals self-report ART status". Can the authors explain more about this social desirability bias? Do you expect that the proportion on ART is overestimated by the data, due to the fact that untreated HIV-infected individuals might falsely affirm that they are on ART (social desirability)? Moreover, as the 17 free parameters are not clearly described (at least I have not found any table/list that describe them in details), it is difficult to understand why the model underestimates the data. Intuitively, I expected that the value of the 17 free parameters were selected by the algorithm so that the model could accurately describe the data (percent on ART). Why is it not the case?
