# Supplementary material for: PopART-IBM, a highly efficient stochastic individual-based simulation model of generalised HIV epidemics developed in the context of the HPTN 071 (PopART) trial
Source: PLoS Comput Biol. 2021 Sep 2;17(9):e1009301. doi: 10.1371/journal.pcbi.1009301 (PMC8478209; doi:10.1371/journal.pcbi.1009301)
Supplement: Attachment — Submitted filename: ploscb reviewer responses_final.docx [file pcbi.1009301.s005.docx]

**Responses to reviews of “PopART-IBM, a highly efficient stochastic individual-based simulation model of generalised HIV epidemics developed in the context of the HPTN 071 (PopART) trial” from initial submission to PLOS Computational Biology**

*6^th^ January 2020*

___________________________________________________________________________

Reviewer 1:	Reviews in document **“PopART-IBM for PLOS Computational Biology.pdf”**

***1.1 This article appears to be improperly formatted for PLOS Computational Biology.***

Thank you for raising this issue. We are not aware of the specific concerns surrounding formatting of our initial submission, however, the resubmission guidelines for PLOS Computational Biology are more stringent than initial submissions and we have adjusted our resubmitted manuscript accordingly. The figures have now been submitted individually which should resolve any issues in relation to legibility.

***1.2 the authors delegate crucial details, (e.g., much of Section S3, including Table S3.1, and Section S6) of their new model to the Supplementary Material, providing only generic information in the main article. I think that it is fine to leave excessive details such as those comprising Sections S2, S4, and Section S5 in the Supplementary Material, but the mathematical basis behind, parameterisation of, and validation of the novel model being presented should absolutely be laid out within the main paper.***

Thank you for this suggestion. We have moved the description of the partnership formation algorithm into the main text and included a table of key partnership-related parameters in the main text (table 1). The full description is still kept in its entirety in the Supplementary Materials.

We have added an additional section ‘Model Validation’ which provides an overview of the validation outlined in Section 6 of the Supplementary Material.

We have also included further detail on the model within the main text by including the figure of the HIV disease progression (as suggested by reviewer 3).

***1.3 … the summary statistics chosen by the authors should be clearly presented, perhaps in a table, and discussed in the main paper. The authors have alluded to the computational costliness and complexity of running such models as theirs, so I assume this is the reason behind using ABC rather than a full likelihood description. However, the authors should then stress the importance of the choice and dimensionality of their summary statistics within the main article.***

The chosen summary statistics are outlined in the third paragraph of the section “Model parameterization and calibration” in the main paper. We have provided further illustration of how the dimensionality is broken down by age group, sex, and time (round). The summary statistics are also visually presented in figure 3.

An ABC approach was adopted for several reasons: 1) ABC has been applied to many different types of data (including phylogenies e.g. Saulnier et al., 2017) and therefore allowed this extension using the diverse data sources available within HPTN 071 (PopART); 2) there are many packages available for implementing ABC algorithms in a number of computer languages (ubiquity and availability); 3) EasyABC has had reasonable coverage and has therefore been tested by, and is accessible to, researchers from a range of fields (validity and familiarity); 4) EasyABC has been available for almost a decade with continued development and maintenance (version 1.0 released in 2012, first published to CRAN in 2015) (persistence and support); 5) EasyABC implements several different ABC algorithms (so different algorithms can be implemented with minor changes of hyperparameters) (simplicity); 6) ABC algorithms via EasyABC are amenable to parallelization (computational speed); and 7) ABC is commonly paired with dynamical models (e.g. Toni et al., (2009) has >1000 citations and Beaumont (2010) has >900 citations) (acceptance within the field).

We note that a function similar to a likelihood can be used as a distance metric within an ABC-like framework and that specifying a likelihood with partially-observed or missing data can be non-trivial. We appreciate Approximate Bayesian Computation (ABC) is only one approach for parameterizing the POPART-IBM and have now discussed this in the new section “Model parameterization and calibration”. We welcome future pairings of the model with other inference frameworks.

***1.4 In the .pdf file provided for reviewers, the quality of the figures in the main article is very poor, with very low resolution. I can barely read the numbers on the x-axes of the graphs in Figures 3a and 3b. A duplicate of Figure 1 appears in the Supplementary Material (S1) and has better resolution. In fact, all of the figures in the Supplementary Material are of better quality, so I assume the way the .pdf file of the main paper was generated messed up the figures. That said, the authors should ensure good quality of their figures upon final submission.***

Thank you for bringing this to our attention. High quality TIF and PDF files have been included separate to the manuscript in the resubmission to avoid any unnecessary compression.

***1.5 Paragraph 2 in the Background section states, “Phylogenetic studies are starting to provide new quantitative insights into transmission dynamics” without a citation (e.g., doi: 10.1093/ve/vey037). As an aside, I would recommend removing the “are starting to” part of this sentence as even phylodynamic (joint inference of phylogenetic and epidemiological information) models have been around since 2004, (doi: 10.1126/science.1090727), and there have been many new phylodynamic models, extensions of preexisting ones, and data analyses using them since then.***

Thank you highlighting this. We have adjusted the text in the Background section as suggested and included a citation.

***1.6 Top of Page 9, in the HIV transmission section, there is the statement, “The relative transmission rates are determined from published literature on HIV transmission in serodiscordant couples in sub Saharan Africa,” with no citation.***

We have now included the relevant references in the section on HIV transmission. Thank you for raising this point.

***1.7 Why did the authors choose the distance measure of 9% for using APMC-ABC, as opposed to, for example, 5%? How does the model perform under other values? Presumably the smaller the number, the better the statistics, but also the longer the model will need to run. I urge the authors to give us an idea of how different values affect both their model performance and computation time.***

Thank you for raising this important point. The values used in this analysis have been updated. It was written incorrectly, and the stopping rule of 5% was used in the analysis.

We have updated the main text to mention the 5% stopping rule and provided an idea of the time required to run such a calibration. The speed of computation on our cluster (and most university clusters that have shared access) depends on queuing time and the scheduler (which we do not have fine control over). We would therefore caution against generalizing the compute time of the calibration process as there are a large number of variables (parameterization, variability of the data, model structure, hardware, compiler optimisation) that will affect compute time when simulations are run at scale. This is why we had previously reported single-simulation compute times. However, we have included runtimes on our cluster for different values of this parameter.

We still hold that an acceptance criterion of 0.09 is acceptable to use. The acceptance criterion of 0.09 (the stopping criterion) was originally chosen when running at scale as it provided a compromise between the goals of parameter inference and fitting, and the practicalities of running an inference framework on 21 communities as part of a clinical trial (i.e. with time constraints). Using a value of 0.09, the inference framework for a single community can be run overnight on Oxford’s BMRC cluster. The default value for this hyperparameter within the EasyABC package is 0.05. However, examples in the EasyABC documentation use values up to 0.40 and extended analyses in Lenormand et al., 2013 (the reference for the algorithm) investigate hyperparameter values between 0.01 and 0.2. Lenormand et al. (2013) conclude *that p_accmin_ < 0.1 give good results for the criterion of “number of simulations” x L^2^ (distance measure)*.

***1.8 Difficulties running the model.***

Thanks for bringing this to our attention. Parameter values were not previously included in the public version of the model and this has now been amended. The publicly-facing repository (<https://github.com/BDI-pathogens/POPART-IBM>) has now been updated with further information on how to run the model, including in the Windows operating system, parameter values to run the model, data dictionaries for parameters and output files, and units tests of the model are included.

***1.9 The Supplementary Material states in Section S6 that the code should be accessible to Mac OS X, Windows, and Ubuntu users. But it appears that either more detailed instructions or possibly some debugging is required in order to run POPART-IBM/ reproduce the authors’ results.***

As mentioned in the previous comment, we have updated the publicly-facing repository so that the model should now run. Thank you for highlighting this.

___________________________________________________________________________

Reviewer 2:	Reviews in document **“review4.docx”**

***2.1 … the authors should explain in more details how this short computational time influence the calibration process. Are the other IBMs that the authors describe in the discussion also able to estimate some of their parameters or is their computational time too long for that? If they are able, how many parameters do they estimate? In addition, it would be interesting to know how much the whole calibration process takes, and not only how much one simulation takes.***

Thank you for raising these important points. We have included additional details of the calibration process in the main text including overall runtime of the calibration process. We caution against generalizing this estimate as it will depend on a large number of model parameters (HIV prevalence impacts runtime also as highlighted in figure 2), hyperparameters of the calibration process (we have reported overall runtime for two values of the acceptance probability) and hardware upon which the calibration framework is run (we have described the high-performance cluster upon which we ran the calibration).

We have also added additional text in the Introduction to stress that this work is focused on the model itself and not the calibration framework (which has been published previously, Lenormand et al., 2013, as cited in the main text).

Several other approaches have been suggested for parameterizing complex models. However, in a systematic review of approaches to calibration of IBMs, Hazelbag et al., (2020) mention ABC is one of the most widely used methods.

The model described in Andrianakis et al., (2015), that is a “dynamic, event-driven, individual-based stochastic“ model applied to HIV in Uganda, reports a single simulation took between “10 minutes and 3 hours” which would likely preclude using ABC. As such, the authors use history matching (approximating the complex model with a computational efficient Bayesian representation, called an “emulator”). They estimate 22 parameters in their approach.

We were only able to find one other HIV IBM of those listed in Hazelbag et al., (2020) where calibration was listed: the CEPAC Dynamic Model (McCormick et al., (2014)) has a reported runtime, which was typically 3-6 hours for a single run using a month timestep to simulate over 100 years starting in 1935 with a population of 100,000. They estimate 12 parameters.

***2.2 Another strength of the model is that it reproduced the PopART intervention and its impact on the study community (patch 0) and its proximate surrounding area (patch 1). However, the authors did not use this feature of the model to compare the impact of different interventions (PopART vs no intervention) on both patches. The authors should explain why (see also my second comment in the Results section).***

Thank you for raising this point and for the interest in the CHiPs intervention as part of the HPTN 071 (PopART) trial. While the model has been used as part of the trial, we present the model as a general simulator for HIV epidemics in communities with HIV prevalence >10%. We have presented counterfactual results so that the results are applicable in a more general context.

We will present modelling results of the trial in a forthcoming paper which will cover the results for all 21 communities and projections of the PopART/CHiPs intervention in more detail.

We have, however, highlighted that the modelled CHiPs intervention uses age/sex stratified coverage that was observed in the trial and that this formulation could be applied to model other interventions that are targeted at specific age groups and sexes (section “Healthcare and prevention” of Methods).

***2.3 … the PopART intervention could be quickly described in the main manuscript as you did in the supplementary material S1.8.***

Thank you for highlighting this. We have included an additional summary of the PopART intervention in the main text, from section S1.8.

***2.4 The model does not differentiate between the different ART regimens (NNRTI-based, DTG-based). It would be good to acknowledge that and explain why such simplification might not drastically change the results (did Rwanda already start DTG?).***

The HPTN 071 (PopART) trial was primarily looking at the impact of a combination HIV prevention package, including home-based testing. The model focused on these aspects of the trial rather than different ART regimens, although the effects of different ART regimens on HIV transmission could be simulated by modifying the ART effectiveness and adherence-related parameters. Thank you for raising this point, we have highlighted this as a limitation in the Discussion in the main text.

***2.5 The authors should also clarify the research question. Does this paper aim at showing the high computational efficiency of the model or does it aim at describing model results about the impact of PopART intervention on the HIV-epidemic? Currently, it seems to me that the paper mixes a bit the two aims.***

We have now further highlighted in the main text that the focus is on the model and not the outcome of the HPTN 071 (PopART) trial or the calibration process. It is necessary to highlight the calibration process to illustrate a “realistic” deployment of such a model.

***2.6 I did not find specific information on both prior and posterior distributions of the 17 calibrated parameters. See also my second comments in the Discussion section.***

Thank you for highlighting this omission. We have now updated the main text to include a description of the parameters in the calibration framework and their prior distributions. We have included a figure of the posterior distribution of parameters in the Supplementary Materials.

***2.7 It would have been much easier to review this paper with line and page numbers.***

We have now added line numbers and page numbers.

***2.8 The quality of the pictures is quite low, making them hard to read.***

Apologies for the low-quality figures and thank you for raising this. It is not clear what has caused the poor quality. The submitted figures (and resubmitted figures) are high quality TIFF files.

***2.9 2nd paragraph: I am not sure to understand what granular exactly means here in the context of mathematical modelling. Consider rephrase it.***

We have now clarified the use of “granular” in the second paragraph of the Background section of the main text.

***2.10 You should also add the limitations of IBM models, e.g. that such complex models are difficult to parametrize/calibrate and that's why simpler model, such as compartmental models, are preferred, as they are easier to control and do not make unverifiable assumptions.***

The Discussion includes an extensive list of limitations of the model. Age- and gender/sex- stratified models of HIV transmission that include various levels of treatment and HIV disease progression can also be highly complex. There comes a point where complexity of the compartmental model makes such models unwieldly to manage and individual-based models are justified and we have included this in the Introduction (“Background” paragraph 3). We have also included figure 6, an illustration of a transmission network generated by the model and argued that the generation of such transmission networks from IBMs as an additional benefit of such models over compartmental models (see Results section “Epidemic dynamics” and Discussion paragraph 3).

***2.11 Figure 1: To me, the Figure 1 is far too simple and does not reflect the complexity of the model. I know that it is impossible to summarize all the model features in one figure but I expected more details. For example, to explain how your IBM works, the author could use the same representation as in Figure 5 (but with less individuals), and show the trajectory of a few hypothetical individuals (split by patches) and represent the following events: birth, deaths, partnership (by linking two individuals), HIV infections, care cascade, interventions (testing, PopART intervention). You could use different colors for the different features (as you currently do in Figure 1 with the 6 features), keep the "puzzle" that explains the 6 features next to the representation of your IBM.***

Thank you for raising this. We have now updated the figure to include the metapopulation structure. The schematic in figure 1 represents the modular approach to the codebase of the model. We have updated the figure caption to highlight this. We therefore believe this is a useful representation of the module components. We have adjusted the figure so that the patch structure is larger and there is text highlighting that the presented processes occur within each patch.

***2.12 The author could make a wider use of the extended supplementary material. For example, when describing how partnership is modelled, the author could add a reference to the section in the supplementary material that describes that in details. The author could add a sentence explaining how the new partnerships are formed (and which individuals are selected for each new partnership) or at least add a reference to the supplementary material.***

Thank you raising this point. Within the main text we have highlighted the sections of the Supplementary Material that provide additional details.

***2.13 The Model section could be split in two subsection: 1) Model structure, 2) Model parametrization/simulation, for more clarity.***

Thank you for this suggestion. We have not split the Methods into “Model structure” and “Model parameterisation and model deployment”.

***2.14 The authors could add a table that summarizes the 17 parameters that are estimated through the calibration process. In the current version, it is unclear which parameters are estimated and why the authors chose these parameters.***

Thank you for raising this point. We have now updated the main text to include a table of parameters estimated in the calibration process. The choice of parameters is mentioned in the main text (section “Model parameterization and calibration” paragraph 1).

***2.15 In the "Measuring computational efficiency" section, the authors explain that the simulations start in 1900 and end in 2020, corresponding to 120 years. In the Results section, it is written that they model 130 years of the epidemic. Why is it different? In addition, it is not clear why the model has to start so early in 1900. As far as I understood, the authors need some simulation time before the start of the HIV epidemic to estimate the age distribution of the population (i.e. to have a "stabilized" age distribution). Why cannot the authors simply take the known age distribution at the beginning of the epidemic and start the model at that time?***

Thank you for highlighting this. We have updated the main text so that the duration of the simulated epidemic is consistent. All simulations are over 120 years in these analyses. The main reason for simulating before the start of the HIV epidemic is for the partnership formation and dissolution processes to “stabilize”. This has been highlighted in the section entitled “Baseline population and initial conditions” in the main text.

***2.16 The authors claim that the simulation lasts about 30 seconds. In the "Calibration" subsection, they say that the ABC algorithm required 131,000 simulations. This would correspond to 131,000 times 30 seconds, i.e. more than 45 days. Does the model really takes so much time? Some clarity is needed here. Overall, it would be useful to know how much time the model takes to estimate the 17 parameters, and not only how much time it needs to run one simulation (i.e. 30 seconds).***

The focus of this paper is on the model, not the inference framework – the inference framework has already been published in Lenormand et al., 2013 and the EasyABC package has been used in numerous applications. We have therefore focused on reporting runtime for a single “typical” simulation (i.e. simulation of a generalized epidemic) as this is likely to be more consistent across different applications of the model. We have now included approximate estimates of the runtime of the complete calibration procedure, as used in this work. However, we strongly caution against using these as estimates for other researchers and still hold that quoting runtime of single simulations is more useful. This is because of the huge number of factors influencing runtime within a calibration and because the calibration process itself (in the nature of being exploratory) often chooses parameter sets that do not lead to simulations of generalized epidemics. We have also included the hardware configuration on which the calibration was run – Oxford’s BMRC cluster using 12 shared cores. The runtime for the whole calibration for 131,000 simulations is around 37 hours. This is shorter than 45 days because of 1) parallelization using 12 cores and 2) because the runtime of some simulations is shorter than 30 seconds due to choices of parameter space that lead to stochastic extinction (and therefore run in a matter of seconds). Calibrations that are initialized with a different random seed will have different runtimes for the same community. We have highlighted this in the section on “calibration”. Furthermore, the model had the ability to run in a quicker “calibration” state where only essential information needed for calibration is written to disk (writing to file is a computationally time-expensive task) – figure 3 is run with typical output being written to disk whereas the calibration experiments are always run with only the bare minimum output written to disk. We have mentioned this in the main text and the caption of figure 3.

***2.17 Figure 4 does not bring anything new, in the sense that you do not use the features of the model (i.e. considering the impact of PopART intervention). It would be much more interesting to present model estimations of the impact of the different "packages" of interventions as described in the PopART. Moreover, it would also be interesting to split the results by patches.***

Thank you for raising this point. As mentioned in review 2.2, although the model has been used as part of the trial, we present the model as a general simulator for HIV epidemics in communities with HIV prevalence >10%. We have presented counterfactual results so that the results are applicable in a more general context. As mentioned above, we will present modelling results of the trial in a forthcoming paper which will cover the results for all 21 communities and projections of the PopART/CHiPs intervention in more detail.

The metapopulation structure (different patches) allows isolation of the impact of interventions that may be applied in the surrounding area. As such, we have focused on the “inside” patch (i.e. the patch that represents the PopART community).

***2.18 The authors wrote: "In addition, model predictions for the proportion of PLHIV on ART are somewhat lower than the data, though this may be expected due to social desirability biases when individuals self-report ART status". Can the authors explain more about this social desirability bias? Do you expect that the proportion on ART is overestimated by the data, due to the fact that untreated HIV-infected individuals might falsely affirm that they are on ART (social desirability)? Moreover, as the 17 free parameters are not clearly described (at least I have not found any table/list that describe them in details), it is difficult to understand why the model underestimates the data. Intuitively, I expected that the value of the 17 free parameters were selected by the algorithm so that the model could accurately describe the data (percent on ART). Why is it not the case?***

Thank you for raising this point and we are happy to provide clarification. Yes, the data likely over-estimate the proportion on ART. This has previously been reported in the context of HIV (e.g. Simoni et al., 2006 and Wilson et al., 2009) and in medication adherence more generally (e.g. Stirratt et al., 2015). To our knowledge, the precise reasons for ART usage often being overestimated when using self-reported data are not clear.

In keeping with the design principles of the model, namely parsimony, we did not include a parameter for self-reporting bias in ART usage in the model. The model under-estimates the data because there is no mechanism in the model to account for self-reporting bias of ART usage. The inference framework is highlighting that this piece of data is inconsistent with the other data source (conditional on the structure of the model). It is not clear to us whether this should be part of data pre-processing or should be taken into account in the model. Different approaches to collecting data on ART usage (not self-reported ART usage) would likely avoid such biases (Simoni et al., 2006).

Regardless of how self-reporting bias it is taken into account, the aim of the model is to estimate the true proportion on ART, not the proportion that report being on ART (i.e. what is shown in the data). The model results show the proportion on ART that are most consistent with the other data (i.e. proportion aware and proportion virally suppressed) and therefore the model implicitly accounts for this overestimation of self-reporting bias. The data on viral suppression are from laboratory testing so represent a “ground truth”.

Furthermore, the focus of this manuscript is on the model itself. Future work will focus on the calibration process and fit to data.

In regards to thorough descriptions of the parameters, we have added a description of the calibrated parameters (Table 2). We have also updated the model repository to include a complete dictionary explaining all the parameters in the model (<https://github.com/BDI-pathogens/POPART-IBM>).

___________________________________________________________________________

Reviewer 3:	Reviews in document **Reviewer comments_01.pdf**

***3.1 Under model parameterization and calibration, the authors could possibly present some key mathematical expressions from the supplementary in the main manuscript for ease of readability and flow. In case a reader is interested in understanding further details then can easily refer to the supplementary document.***

***With the mathematical expressions the authors can then consider summarizing the Methods section again for ease of readability.***

We have now included additional details regarding the parameterization of the partnership formation algorithm, details of the HIV disease progression in the absence of ART, and more detailed description of the calibration to the main text.

***3.2 Figure S4.1 can also be included in the main manuscript.***

Thank you for this suggestion. As suggested, we have included this figure in the main text now.

***3.3 Figure S7.3 does not seem to be in the supplementary document.***

Thank you for pointing this out. We have amended the main text.

***3.4 The supplementary document is huge (about 84) pages. Can the authors consider reducing it to what is extremely necessary for reference?***

Thank you raising this. As outlined in the response to the other reviews we have moved some of the Supplementary Material (SM) into the main text. This has not reduced the size of the SM substantially and we hope to keep the SM as comprehensive as possible.

**References**

Andrianakis I, Vernon IR, McCreesh N, McKinley TJ, Oakley JE, Nsubuga RN, et al. (2015) Bayesian History Matching of Complex Infectious Disease Models Using Emulation: A Tutorial and a Case Study on HIV in Uganda. PLoS Comput Biol 11(1): e1003968. <https://doi.org/10.1371/journal.pcbi.1003968>

Beaumont MA (2010) Approximate Bayesian Computation in Evolution and Ecology

Annual Review of Ecology, Evolution, and Systematics. 41:1, 379-406

Hazelbag CM, Dushoff J, Dominic EM, Mthombothi ZE, Delva W (2020) Calibration of individual-based models to epidemiological data: A systematic review. PLoS Comput Biol 16(5): e1007893. https://doi.org/10.1371/journal.pcbi.1007893

M. Lenormand, F. Jabot, G. Deffuant. Adaptive approximate Bayesian computation for complex models. Computational Statistics, Springer Verlag, 2013, 28 (6), pp.2777-2796. 10.1007/s00180-013- 0428-3.

McCormick AW, Abuelezam NN, Rhode ER, Hou T, Walensky RP, Pei PP, et al. Development, Calibration and Performance of an HIV Transmission Model Incorporating Natural History and Behavioral Patterns: Application in South Africa. PLOS ONE. 2014;9(5):e98272.

Saulnier E, Gascuel O, Alizon S (2017) Inferring epidemiological parameters from phylogenies using regression-ABC: A comparative study. PLoS Comput Biol 13(3): e1005416. https://doi.org/10.1371/journal.pcbi.1005416

Simoni JM, Kurth AE, Pearson CR, Pantalone DW, Merrill JO, Frick PA. Self-report measures of antiretroviral therapy adherence: A review with recommendations for HIV research and clinical management. AIDS Behav. 2006 May;10(3):227-45. doi: 10.1007/s10461-006-9078-6.

Stirratt MJ, Dunbar-Jacob J, Crane HM, Simoni JM, Czajkowski S, Hilliard ME, Aikens JE, Hunter CM, Velligan DI, Huntley K, Ogedegbe G, Rand CS, Schron E, Nilsen WJ. Self-report measures of medication adherence behavior: recommendations on optimal use. Transl Behav Med. 2015 Dec;5(4):470-82. doi: 10.1007/s13142-015-0315-2.

Toni Tina, Welch David, Strelkowa Natalja, Ipsen Andreas and Stumpf Michael P.H (2009) Approximate Bayesian computation scheme for parameter inference and model selection in dynamical systemsJ. R. Soc. Interface.6187–202 http://doi.org/10.1098/rsif.2008.0172

Wilson IB, Carter AE, Berg KM. Improving the self-report of HIV antiretroviral medication adherence: is the glass half full or half empty? Curr HIV/AIDS Rep. 2009 Nov;6(4):177-86. doi: 10.1007/s11904-009-0024-x
