# Supplementary material for: PopART-IBM, a highly efficient stochastic individual-based simulation model of generalised HIV epidemics developed in the context of the HPTN 071 (PopART) trial
Source: PLoS Comput Biol. 2021 Sep 2;17(9):e1009301. doi: 10.1371/journal.pcbi.1009301 (PMC8478209; doi:10.1371/journal.pcbi.1009301)
Supplement: Attachment — Submitted filename: ploscb reviewer responses_final_July2021.docx [file pcbi.1009301.s006.docx]

**Reviewer #1:**

***The manuscript is much improved, readable and flowing.
Comment:
Is it necessary to have both the abstract and author summary?***

We have now modified the author summary to make it more distinct from the abstract.

**Reviewer #2:**

***The manuscript has been greatly improved since the first submission and is ripe for publication!***

We thank the reviewer for their kind comment!

**I have already noted this to the editor, but for the relief of the authors I'll note this here, too (especially if it is brought up again by my fellow reviewers): Upon review of the second submission, I noticed that downloading the individual figure files produced high quality images, which the authors were quite certain they had submitted from the start. However, within the compiled pdf of the whole paper, as provided for the reviewers, the figures again appear very poor and with low resolution. Thus, it seems that the compilation process used by PLOS Computational Biology to yield a pdf for review is messing with the quality of the figures within the manuscript. This should be noted for future submissions and of course not held against the authors.**

Thank you for checking this – we are glad that the figures are now of the requisite quality.

**Reviewer #3: Main comment:
The authors replied with full details to almost all the concerns/questions that were raised. Only one concern is remaining, which is related to the comment 2.17. To me, the Figure 5 (which was Figure 4 in the previous version) still does not bring anything that justifies keeping it in the main text. Moreover, as the focus of this manuscript is on the model (and not the interpretation of the results), describing the dynamic of HIV as predicted by the model for a single counterfactual scenario (lines 493-502) do not really help the reader understand the features of the model.
Therefore, I would consider either presenting different counterfactual scenarios (e.g. the 3 arms of the study, in order to show what the model is able to compare, even if I understand that the exhaustive comparison of these arms will be done in a subsequent paper) or removing Figure 5 and the related paragraphs describing the results.**

We thank you for this comment. As you say, it can certainly be argued that the focus of the manuscript should be on the model. However, we feel that showing a real-world application of the model will provide the reader with a better idea of how such a model can be used in practice, while showing calibrated prevalence trends is important as part of validating the model. As we stated in reply to your previous comment 2.17, we will provide more detailed scenarios in future publications, but we feel that the current result shown in Figure 5 is also of interest from an epidemiological point of view, providing a simple but in our opinion important insight – namely that while HIV prevalence and incidence may stabilise, the number of people on ART can still grow over time in a growing population, and that this could potentially occur in the modelled community in Zambia. Finally, given that we hope that the description of the model in this manuscript will be of use to other modellers looking to develop or refine their own individual-based models, we believe that it is important to showcase this result here as it highlights the importance of including realistic population growth in HIV models.

***Minor comments:***

***There is a small typo (Figure55 should be Figure5).***

We could not find this typo in the manuscript. However, on line 464 the figure caption was “Figure5”, and this has now been changed to “Figure 5”.

***There are two Tables 2. The first one should be Table 1.***

We thank the reviewer for pointing this out. There was an error with the field code which did not show up at first on our manuscript, but has now been corrected.

***The Table 2 (the second table describing the parameters) really helps understand which parameters are involved in the model. However, I would remove the “technical” names of the parameters (first row), as these names are only used in the model script.***

Thank you for this suggestion – we have removed the first column as suggested, and edited one parameter description in the table to clarify.
